# Supplementary material for: Automated assembly of molecular mechanisms at scale from text mining and curated databases
Source: Mol Syst Biol. 2023 Mar 20;19(5):e11325. doi: 10.15252/msb.202211325 (PMC10167483; doi:10.15252/msb.202211325)
Supplement: Supplementary file 1 — Expanded View Figures PDF [file MSB-19-e11325-s005.pdf]

# Expanded View Figures

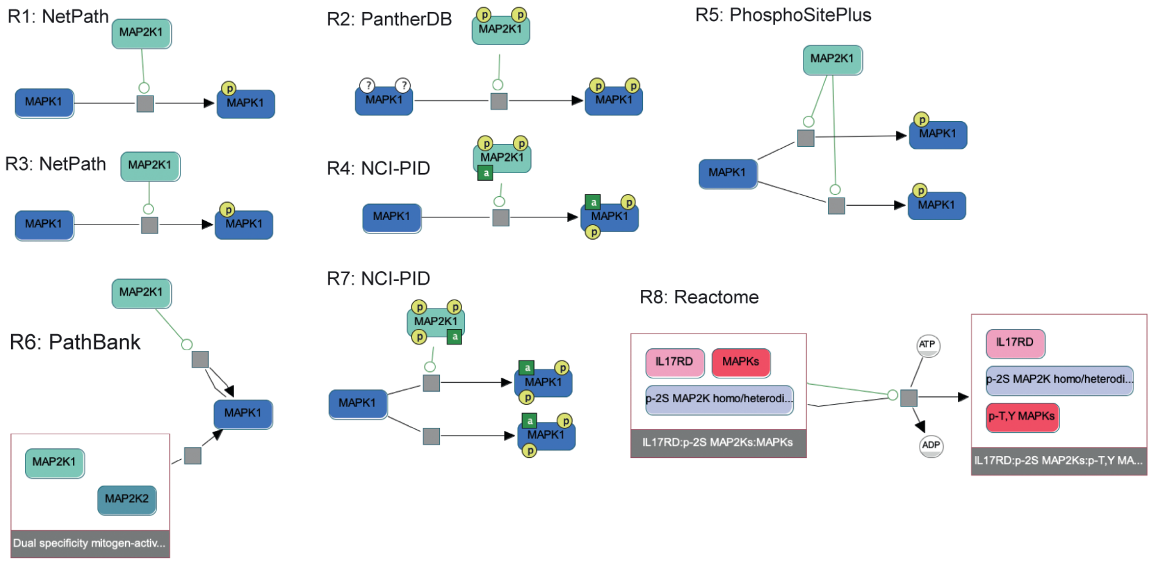


**Figure EV1. Differences in curation practices across databases integrated by Pathway Commons.**

A subgraph of the "paths-from-to" query between MAP2K1 and MAPK1 obtained from Pathway Commons and visualized using the ChiBE software (Babur *et al*, 2009). Each biochemical reaction (R1–R8) depicts a different curation of the same reaction in which MAP2K1 phosphorylates MAPK1. The original source database (e.g., NetPath) is shown next to each reaction. Inconsistencies include (i) the reaction structure itself, with some specifying a single step phosphorylation of two sites (e.g., R4) while others specify single-site phosphorylation (e.g., R1), or the explicit representation of ADP and ATP as part of the reaction (R8); (ii) the phosphorylation status of MAP2K1, with no phosphorylation status given in R1, R3, R5, R6, and R8, two phosphorylation sites indicated in R2, R4, and three phosphorylation sites in R7; (iii) the initial state of MAPK1, with R2 explicitly indicating unphosphorylated status, while other reactions do not make this explicit; (iv) the final state of MAPK1, with some reactions representing MAPK1 phosphorylation on an unspecified site (R1 and R3), and others providing specific phosphorylation sites (e.g., R2); (v) the specification of active states, with R4 being the only reaction representing MAP2K1 explicitly as active, while R4 and R7 are the only reactions specifying that MAPK1 is active after phosphorylation; and (vi) the presence of other co-factors such as IL17RD (R8) as part of the reaction.

© 2023 The Authors 

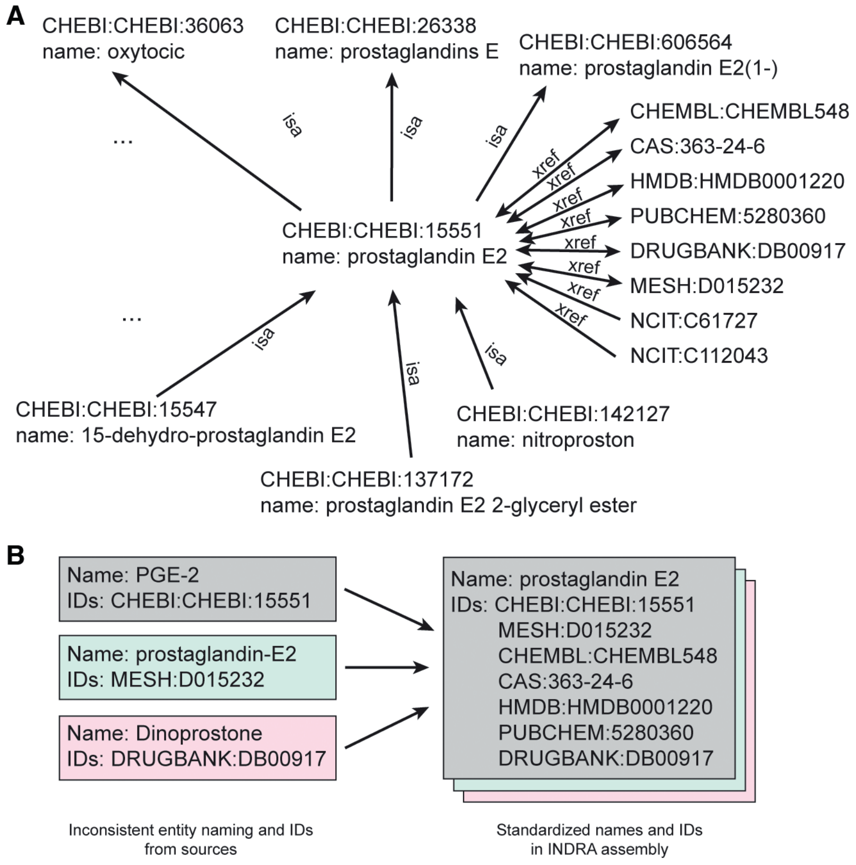


**Figure EV2. Ontology graph guiding INDRA knowledge assembly.**

A A subgraph of the INDRA ontology graph showing the neighborhood of the node representing “prostaglandin E2” in the ChEBI database (CHEBI: 15551). Edges represent “isa” relationships to more general terms (and from more specific terms), and “xref” edges represent identifier equivalence to nodes representing entries in other databases including MeSH, DrugBank, ChEMBL, CAS, PubChem, and NCIT. Each ontology graph node also provides a name that can be used for standardization and display purposes.

B Example of three entities with inconsistent names and identifiers which, when standardized by INDRA using the ontology graph, are normalized to consistent entities with identical names and sets of identifiers.

 © 2023 The Authors

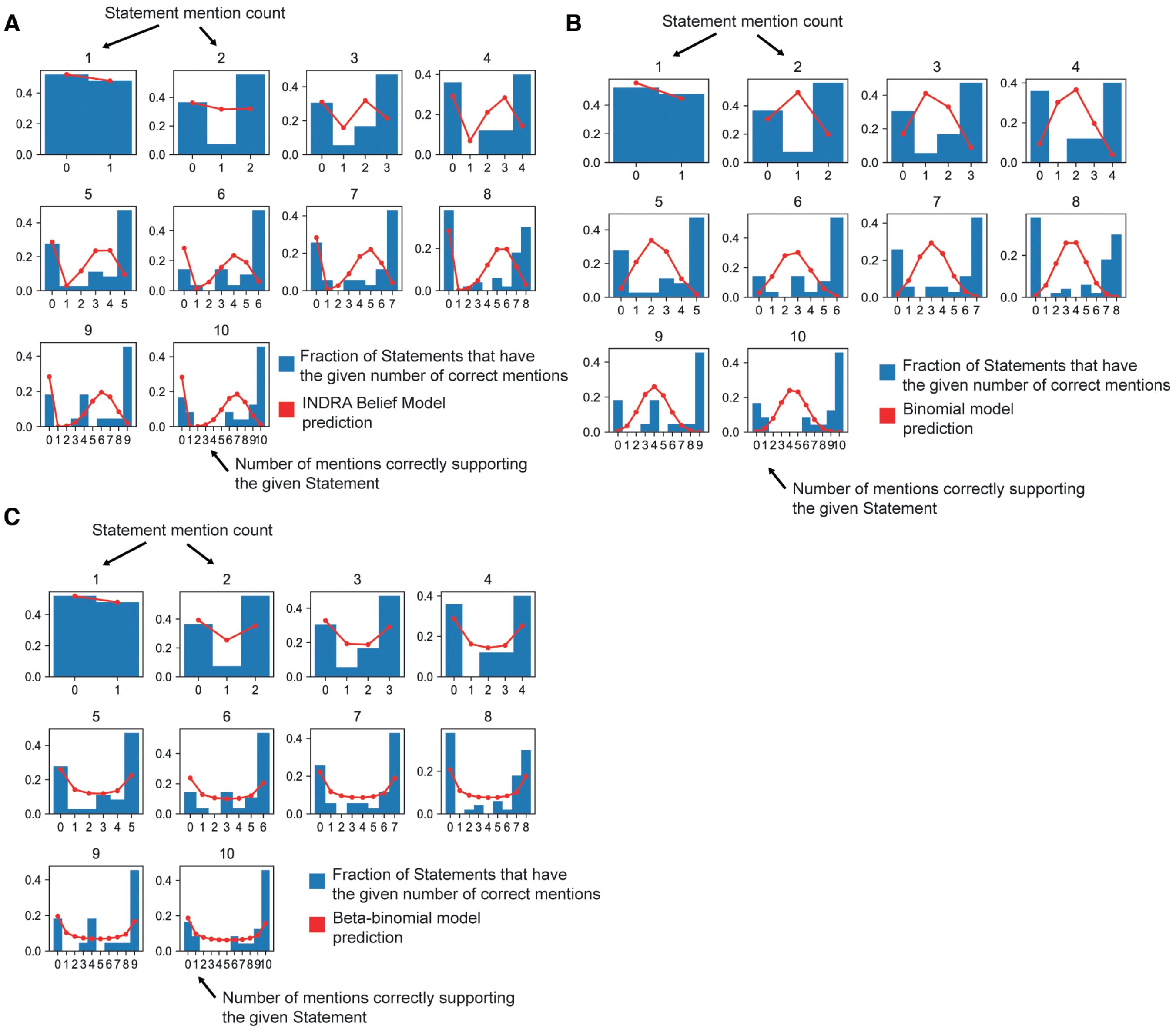


**Figure EV3. Observed and predicted distributions of mentions correctly extracted by Reach for Statements supported by up to 10 Reach mentions.**

A Frequencies of correct mentions predicted by the INDRA Belief Model. The blue bars in each subplot show the frequencies of statements with *k* correctly extracted mentions for *n* total mentions for the Statement (considering mentions from the Reach reader only). The red line in each subplot shows the frequencies of correct mentions expected by the INDRA Belief Model. The INDRA Belief Model expects a substantial proportion of Statements to have an intermediate number of correctly extracted mentions, whereas the empirical data suggests that Statements are more likely to be associated with mentions that are either all correct or incorrect.

B Frequencies of correct mentions expected by the Binomial model. Blue bars are identical to (A).

C Frequencies of correct mentions expected by the Beta-binomial model. Blue bars are identical to (A) and (B). The Beta-binomial model differs from the INDRA Belief Model and Binomial models in that it predicts relatively greater proportions of Statements with mentions that are either all correct or incorrect.

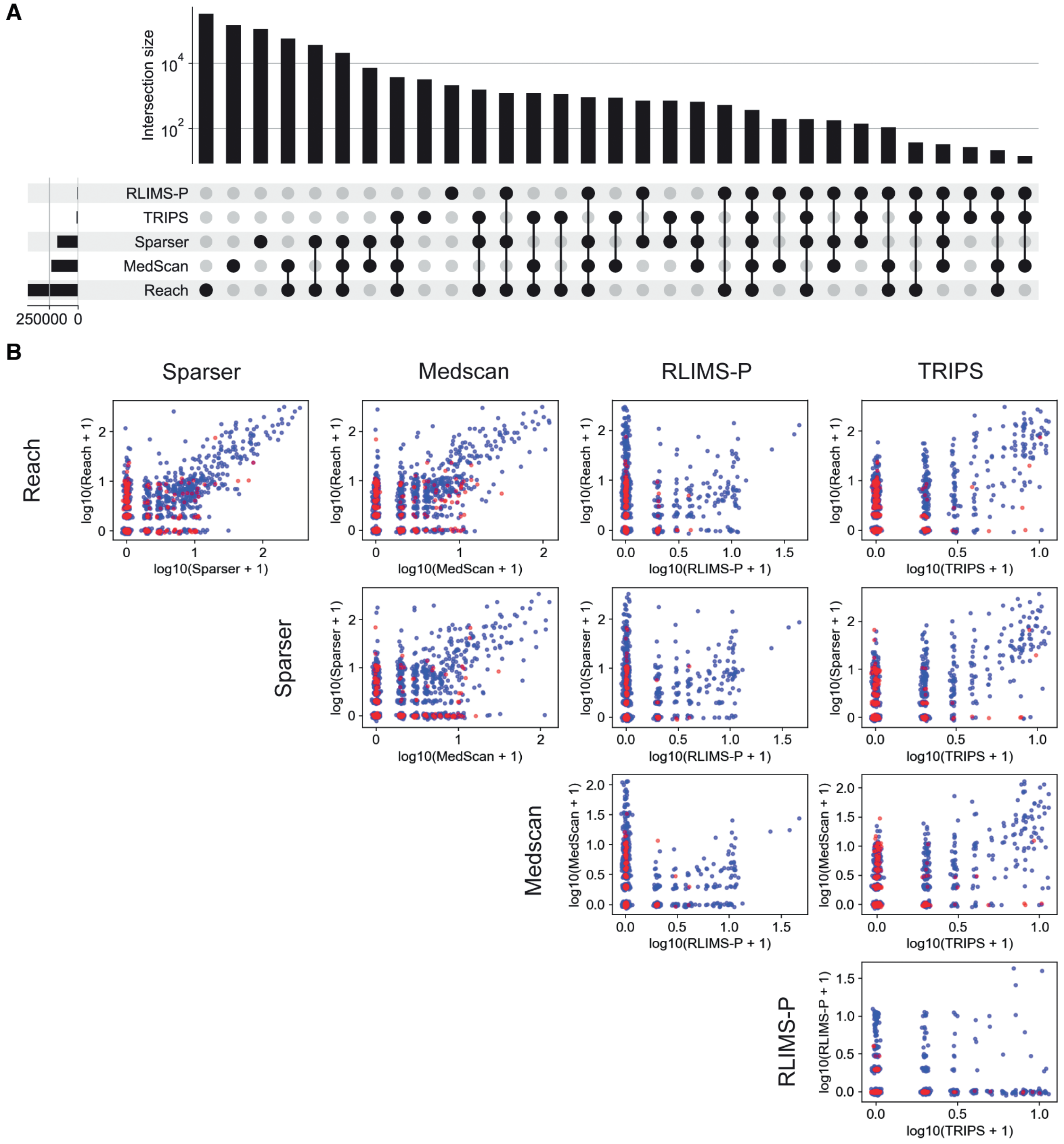


**Figure EV4. Reader overlap and Statement correctness.**

A Upset plot (equivalent to a Venn diagram with more than three sets) of Statement support for five machine reading systems integrated by INDRA. Data are identical to Fig 5A but intersection sizes are plotted on a log scale and all 32 possible reader combinations are shown.

B Multi-reader mention counts and Statement correctness. Each subplot shows the relationship between mention counts from a combination of two readers for manually curated Statements. Blue points represent Statements that were curated as correct; red points were curated as incorrect. A small amount of random jitter has been added to each point to indicate the density of points with fewer mention counts.

 © 2023 The Authors
