# Supplementary material for: Automated assembly of molecular mechanisms at scale from text mining and curated databases
Source: Mol Syst Biol. 2023 Mar 20;19(5):e11325. doi: 10.15252/msb.202211325 (PMC10167483; doi:10.15252/msb.202211325)
Supplement: Supplementary file 2 — Table EV1 [file MSB-19-e11325-s004.docx]

*Expanded View Table EV1. Number of INDRA Statements by type extracted by each reading system from the Benchmark Corpus document set, before filtering or normalization.*

| **Statement type** | **Reach** | **Sparser** | **MedScan** | **TRIPS** | **RLIMS-P** | **ISI/AMR** |
| --- | --- | --- | --- | --- | --- | --- |
| Acetylation | 8100 | 35108 | 490 | 1156 | 0 | 0 |
| Activation | 1374408 | 92852 | 456835 | 8007 | 0 | 0 |
| ActiveForm | 0 | 0 | 0 | 208 | 0 | 0 |
| Autophosphorylation | 0 | 7111 | 0 | 377 | 3825 | 0 |
| Complex | 432602 | 501062 | 173245 | 15117 | 0 | 20272 |
| Conversion | 0 | 0 | 0 | 585 | 0 | 0 |
| Deacetylation | 4080 | 0 | 581 | 379 | 0 | 0 |
| DecreaseAmount | 123038 | 0 | 93996 | 4755 | 0 | 0 |
| Defarnesylation | 7 | 0 | 0 | 0 | 0 | 0 |
| Deglycosylation | 226 | 0 | 8 | 1 | 0 | 0 |
| Dehydroxylation | 93 | 0 | 0 | 0 | 0 | 0 |
| Demethylation | 1975 | 0 | 156 | 10 | 0 | 0 |
| Depalmitoylation | 0 | 92 | 0 | 4 | 0 | 0 |
| Dephosphorylation | 31910 | 19310 | 1261 | 1167 | 0 | 0 |
| Deribosylation | 47 | 0 | 0 | 0 | 0 | 0 |
| Desumoylation | 396 | 435 | 12 | 3 | 0 | 0 |
| Deubiquitination | 4687 | 0 | 0 | 119 | 0 | 0 |
| Farnesylation | 32 | 379 | 2 | 36 | 0 | 0 |
| Geranylgeranylation | 0 | 0 | 5 | 18 | 0 | 0 |
| Glycosylation | 687 | 7596 | 19 | 1 | 0 | 0 |
| Hydroxylation | 924 | 2618 | 80 | 23 | 0 | 0 |
| IncreaseAmount | 215782 | 0 | 279292 | 12935 | 0 | 0 |
| Inhibition | 626221 | 92824 | 305941 | 8651 | 0 | 0 |
| Methylation | 6568 | 67615 | 513 | 1 | 0 | 0 |
| Myristoylation | 0 | 870 | 1 | 23 | 0 | 0 |
| Palmitoylation | 0 | 2131 | 39 | 50 | 0 | 0 |
| Phosphorylation | 122711 | 430612 | 13509 | 22781 | 162343 | 0 |
| Ribosylation | 744 | 147 | 24 | 34 | 0 | 0 |
| Sumoylation | 1324 | 9386 | 35 | 1 | 0 | 0 |
| Translocation | 16815 | 27602 | 0 | 325 | 0 | 0 |
| Ubiquitination | 14815 | 42731 | 1598 | 1933 | 0 | 0 |
