# Supplementary material for: Automated assembly of molecular mechanisms at scale from text mining and curated databases
Source: Mol Syst Biol. 2023 Mar 20;19(5):e11325. doi: 10.15252/msb.202211325 (PMC10167483; doi:10.15252/msb.202211325)
Supplement: Supplementary file 3 — Table EV2 [file MSB-19-e11325-s003.docx]

*Expanded View Table EV2. Comparison of belief models (using AUPRC as the metric) depending on model type, sources included, additional features taken into account, and whether more specific evidences are taken into account based on statement refinement relations. (Note: Statement is abbreviated as Stmt in the table).*

| **Group** | **Row** | **Model** | **Sources** | **Additional Features** | **More specific evidences** | **AUPRC** |
| --- | --- | --- | --- | --- | --- | --- |
| Reader counts only | 1 | **Belief Model** | **Readers** | **N/A** | **No** | **0.917** |
|  | 2 | Random Forest | Readers | None | No | 0.893 |
|  | 3 | Logistic Regression | Readers | None | No | 0.910 |
|  | 4 | k-NN | Readers | None | No | 0.900 |
|  | 5 | SVC | Readers | None | No | 0.891 |
| More features | 6 | Random Forest | Readers | Stmt type, # PMIDs | No | 0.920 |
|  | 7 | Random Forest | Readers | Stmt type, # PMIDs, Avg. evidence len. | No | 0.922 |
|  | 8 | Random Forest | Readers | Stmt type, # PMIDs, "promoter" | No | 0.930 |
|  | 9 | **Random Forest** | **Readers** | **Stmt type, # PMIDs, "promoter", Avg. evidence len.** | **No** | **0.932** |
|  | 10 | Logistic Regression | Readers | Stmt type, # PMIDs | No | 0.923 |
|  | 11 | Logistic Regression | Readers | Stmt type, # PMIDs, Avg. evidence len. | No | 0.924 |
|  | 12 | Logistic Regression | Readers | Stmt type, # PMIDs, "promoter" | No | 0.926 |
|  | 13 | Logistic Regression | Readers | Stmt type, # PMIDs, "promoter", Avg. evidence len. | No | 0.926 |
|  | 14 | k-NN | Readers | Stmt type, # PMIDs | No | 0.908 |
|  | 15 | SVC | Readers | Stmt type, # PMIDs | No | 0.901 |
| Reader counts only, specific evidences | 16 | **Belief Model** | **Readers** | **N/A** | **Yes** | **0.917** |
|  | 17 | Random Forest | Readers | None | Yes | 0.913 |
|  | 18 | Logistic Regression | Readers | None | Yes | 0.912 |
|  | 19 | k-NN | Readers | None | Yes | 0.892 |
|  | 20 | SVC | Readers | None | Yes | 0.885 |
| More features, specific evidences | 21 | Random Forest | Readers | Stmt type, # PMIDs | Yes | 0.929 |
|  | 22 | Random Forest | Readers | Stmt type, # PMIDs, Avg. evidence len. | Yes | 0.930 |
|  | 23 | Random Forest | Readers | Stmt type, # PMIDs, "promoter" | Yes | 0.937 |
|  | 24 | **Random Forest** | **Readers** | **Stmt type, # PMIDs, "promoter", Avg. evidence len.** | **Yes** | **0.937** |
|  | 25 | Logistic Regression | Readers | Stmt type, # PMIDs | Yes | 0.927 |
|  | 26 | Logistic Regression | Readers | Stmt type, # PMIDs, Avg. evidence len. | Yes | 0.927 |
|  | 27 | Logistic Regression | Readers | Stmt type, # PMIDs, "promoter" | Yes | 0.928 |
|  | 28 | Logistic Regression | Readers | Stmt type, # PMIDs, "promoter", Avg. evidence len. | Yes | 0.929 |
|  | 29 | k-NN | Readers | Stmt type, # PMIDs | Yes | 0.901 |
|  | 30 | SVC | Readers | Stmt type, # PMIDs | Yes | 0.895 |
| All sources, more features, specific evidences | 31 | Belief Model | Readers, DBs | N/A | Yes | 0.923 |
|  | 32 | Random Forest | Readers, DBs | None | Yes | 0.928 |
|  | 33 | Random Forest | Readers, DBs | Stmt type, # PMIDs | Yes | 0.937 |
|  | 34 | Random Forest | Readers, DBs | Stmt type, # PMIDs, Avg. evidence len. | Yes | 0.936 |
|  | 35 | Random Forest | Readers, DBs | Stmt type, # PMIDs, "promoter" | Yes | 0.942 |
|  | 36 | **Random Forest** | **Readers, DBs** | **Stmt type, # PMIDs, "promoter", Avg. evidence len.** | **Yes** | **0.942** |
|  | 37 | Logistic Regression | Readers, DBs | None | Yes | 0.924 |
|  | 38 | Logistic Regression | Readers, DBs | Stmt type, # PMIDs | Yes | 0.935 |
|  | 39 | Logistic Regression | Readers, DBs | Stmt type, # PMIDs, Avg. evidence len. | Yes | 0.935 |
|  | 40 | Logistic Regression | Readers, DBs | Stmt type, # PMIDs, "promoter" | Yes | 0.936 |
|  | 41 | Logistic Regression | Readers, DBs | Stmt type, # PMIDs, "promoter", Avg. evidence len. | Yes | 0.937 |
