# Supplementary material for: Automated assembly of molecular mechanisms at scale from text mining and curated databases
Source: Mol Syst Biol. 2023 Mar 20;19(5):e11325. doi: 10.15252/msb.202211325 (PMC10167483; doi:10.15252/msb.202211325)
Supplement: Supplementary file 4 — PDF+ [file MSB-19-e11325-s002.pdf]

Method

TRANSPARENT PROCESS OPEN ACCESS

molecular systems biology

Automated assembly of molecular mechanisms at scale from text mining and curated databases

John A Bachman[1,†], Benjamin M Gyori[1,*,†] & Peter K Sorger[1,2,**]

Abstract

The analysis of omic data depends on machine-readable information about protein interactions, modifications, and activities as found in protein interaction networks, databases of post-translational modifications, and curated models of gene and protein function. These resources typically depend heavily on human curation. Natural language processing systems that read the primary literature have the potential to substantially extend knowledge resources while reducing the burden on human curators. However, machine-reading systems are limited by high error rates and commonly generate fragmentary and redundant information. Here, we describe an approach to precisely assemble molecular mechanisms at scale using multiple natural language processing systems and the Integrated Network and Dynamical Reasoning Assembler (INDRA). INDRA identifies full and partial overlaps in information extracted from published papers and pathway databases, uses predictive models to improve the reliability of machine reading, and thereby assembles individual pieces of information into non-redundant and broadly usable mechanistic knowledge. Using INDRA to create high-quality corpora of causal knowledge we show it is possible to extend protein–protein interaction databases and explain co-dependencies in the Cancer Dependency Map.

Keywords curation; databases; modeling; networks; text mining
Subject Categories Computational Biology; Methods & Resources
DOI 10.15252/msb.202211325 | Received 31 August 2022 | Revised 24 February 2023 | Accepted 27 February 2023 | Published online 20 March 2023
Mol Syst Biol. (2023) 19: e11325

Introduction

Molecular biology is characterized by a sustained effort to acquire and organize mechanistic information about the molecules governing the behavior of cells, tissues, and organisms (Craver & Darden, 2013). "Mechanism" is used rather loosely in this context since it operates on multiple scales from the structural transitions of individual molecules to the myriad interactions mediating signal transduction or tissue morphology, but it is generally understood to involve a description of the properties, modifications, and behaviors of biomolecules in terms of physical and chemical principles. Individual mechanistic discoveries are reported in the biomedical literature, which, with over $3{\cdot}10^7$ articles indexed in PubMed as of 2022, constitutes a substantial public investment in science and an essential source of knowledge. However, results in research papers are generally described in natural language designed for human—not machine—consumption. As the literature has grown, and methods of experimental data collection become more diverse, it has become increasingly difficult for any individual scientist to acquire all of the background knowledge necessary to be an expert in a particular problem and fully interpret complex experimental results (Forscher, 1963). Biomedicine is therefore faced with a substantial problem of knowledge aggregation, harmonization, and assembly.

The bioinformatics community has actively worked to make knowledge more accessible by curating information about molecular mechanisms in a machine-readable form suitable for computational data analysis (Ashburner *et al*, 2000; Schaefer *et al*, 2009; Perfetto *et al*, 2016; Fabregat *et al*, 2018). This has led to the creation of standard representation languages (Hucka *et al*, 2003; Demir *et al*, 2010), and databases that aggregate curated knowledge from multiple primary sources (Jensen *et al*, 2009; Cerami *et al*, 2011; Türei *et al*, 2016). Curated databases form the backbone of many widely used methods of high-throughput data analysis, including gene set and pathway enrichment, and prior knowledge-guided network inference (Babur *et al*, 2021; Dugourd *et al*, 2021). However, the creation of these databases has largely involved human curation of the literature, which is costly and difficult to sustain (Bourne *et al*, 2015). As a result, most databases and online resources are incomplete; for example, the creators of Pathway Commons (which aggregates pathway knowledge from 22 primary human-curated databases) have estimated that their resource covers only 1–3% of the available literature (Valenzuela-Escárcega *et al*, 2018). At the same time, databases such as Pathway Commons contain redundant or conflicting information about the same sets of mechanisms because assembling knowledge into a coherent whole is difficult and is dependent on human expertise and curation. Compounding these difficulties is the increasing volume of published scientific articles, which makes ongoing maintenance of a previously created resource necessary to prevent obsolescence; the fact that curation standards

1 Laboratory of Systems Pharmacology, Harvard Medical School, Boston, MA, USA
2 Department of Systems Biology, Harvard Medical School, Boston, MA, USA
*Corresponding author. Tel: +1 617 432 5118; E-mail: benjamin_gyori@hms.harvard.edu
**Corresponding author. Tel: +1 617 432 6901; E-mail: peter_sorger@hms.harvard.edu
[†]These authors contributed equally to this work

© 2023 The Authors. Published under the terms of the CC BY 4.0 license

and languages evolve along with methods of data collection and analysis further complicates the task of knowledge assembly.

Automated extraction of mechanistic information through literature mining (using natural language processing) has the potential to address many of the challenges associated with manual curation (Ananiadou *et al*, 2015). However, the precision of machine reading systems remains lower than that of human curators, particularly for complex relationships and the subtle language in specific statements about the mechanism (Allen *et al*, 2015; Islamaj Doğan *et al*, 2019; Madan *et al*, 2019). Nevertheless, at the current state of the art, machine reading can extract simple relations (e.g., post-translational modifications and binding and regulatory events) at the literature scale (i.e., from a substantial fraction of the body of $3{\cdot}10^7$ biomedical publications currently available). To accomplish this, a variety of text mining systems have been developed, each with different designs, strengths, and weaknesses. Common steps in these systems include grammatical parsing of sentences, named entity recognition and normalization, also called grounding (i.e., associating entities with a standardized identifier in controlled vocabularies such as HGNC), and event extraction (identifying interactions, transformations, or regulation involving grounded entities). Much of the research in text mining for biology to date has focused on small-scale studies for method validation, but a handful of efforts have aimed to create large-scale resources available for use in data analysis by the broader computational biology community (Yuryev *et al*, 2006; Van Landeghem *et al*, 2013).

A key requirement for the broader use of text mining in biological data analysis is overcoming the relatively low technical precision of current systems. One way to mitigate the effect of text mining errors is to filter out low-confidence extractions based on reliability estimates. General reliability estimates can be derived *a priori* from the published precision scores for specific text mining systems (e.g., Torii *et al*, 2015; Valenzuela-Escárcega *et al*, 2018), but these figures do not account for the fact that error rates can differ substantially for different types of information or sentence structures. An alternative approach is to cross-reference text-mined information against previously curated databases (Holtzapple *et al*, 2020) which yields high-confidence interactions at the expense of the breadth provided by text mining. For single reading systems, redundancy among extractions (i.e., extracting the same information repeatedly from different spans of text) has been shown to associate positively with reliability (Valenzuela-Escárcega *et al*, 2018) but this has not as yet been quantitatively characterized or used to derive reliability scores. In principle, the integration of multiple distinct reading systems with different types and rates of error could provide the information needed to estimate interaction reliability but this has not been previously explored.

Overall, what is still needed are computational tools for the large-scale assembly of both text-mined and curated mechanisms in databases to generate knowledge resources with mechanistic detail and genome scale. Human-generated resources such as Reactome (Fabregat *et al*, 2018) aspire to this but would benefit in scope and currency from human-in-the-loop collaboration with machines. To accomplish this, machine assembly must overcome challenges associated with combining noisy information about mechanisms at different levels of specificity in the face of the technical errors in grounding and event extraction mentioned above. Users of the resulting knowledge will often have different end goals but still need reliable networks and models. Particularly challenging is the assembly of information that can be used to investigate specific mechanisms at the level of the individual reactions, mutations, or drug-binding events—something currently possible on a smaller scale using dynamical systems analysis (Lopez *et al*, 2013) and logic-based modeling (Saez-Rodriguez *et al*, 2009). These more mechanistic networks and models contrast with existing genome-scale networks that commonly involve unsigned node-edge graphs that aggregate diverse types of interactions (genetic, physical, co-localization, etc.) using the simplest possible abstraction.

We previously described a software system, the Integrated Network and Dynamical Reasoning Assembler (INDRA), able to read simplified declarative language and create relatively small mechanistic models that could be executed using dynamical, logic-based, or causal formalisms (Gyori *et al*, 2017). This version of INDRA could, for example, convert “word models” such as “Active ATM activates p53. Active p53 transcribes MDM2, etc.” into dynamical ODE-based models. INDRA accomplishes this using an intermediate representation to decouple the process of knowledge collection from the construction of specific models. More specifically, INDRA normalizes mechanistic information expressed in natural (English) language into a high-level intermediate machine representation called Statements. Statements can then be used directly to create executable models, for example in rule-based languages such as BioNetGen or PySB. The current taxonomy of INDRA Statements accounts for the types of biomolecular processes most commonly involved in intracellular biological networks and signal transduction (e.g., post-translational modifications, positive and negative regulation, binding, and transcriptional regulation) but is extensible to other domains of natural science.

Here, we describe a major extension of the INDRA architecture that allows it to tackle the harder and more generally applicable problem of assembling mechanistic information extracted from the primary research literature at scale (hundreds of thousands of publications). This task presents a set of challenges that are very different from those encountered when converting declarative language into ODE models (Gyori *et al*, 2017). We accomplished reading at scale by combining the results of multiple reading systems with curated mechanisms from a wide range of databases and structured knowledge sources. Used in this way, INDRA identifies duplicate and partially overlapping Statements, allowing for the automated assembly of mechanistic fragments into a nonredundant and coherent set of interactions and subsequently into large-scale knowledge assemblies for use in biocuration and data analysis. We illustrate these capabilities of end-to-end assembly in INDRA by processing publications and databases relevant to human genomics to create a corpus of ~900,000 unique and specified interactions and regulations among human proteins. We found that the overlap between different machine reading systems was surprisingly small (highlighting both the readers’ complementarity and their limitations), but for a given INDRA Statement, the existence of supportive evidence from multiple reading systems was informative of reliability. We used manual curation to quantify the technical error and overlap characteristics of different machine reading systems and then developed predictive models that estimate the reliability of text-mined extractions in the form of a “belief score.” Finally, to evaluate the utility of machine-extracted mechanisms, we used the INDRA-assembled corpus of Statements to prioritize the curation of protein–protein interactions

 © 2023 The Authors

(PPIs) that are not yet captured in the widely used structured knowledgebase, BioGRID (Oughtred *et al*, 2019). We then used the same assembled corpus to identify and explain gene dependency relationships in the Cancer Dependency Map (DepMap) dataset (Meyers *et al*, 2017; Tsherniak *et al*, 2017). In this case, an INDRA-assembled network helped determine statistically significant codependencies between genes, thus allowing for the detection of new codependencies in cancer. INDRA also provided possible mechanistic explanations rooted in the scientific literature for observed DepMap codependencies.

Results

Automated assembly of large knowledgebases from curated databases and machine reading systems raises a series of interconnected, conceptual issues not arising in the conversion of simple declarative natural language into machine readable mechanisms, a problem we previously tackled using the INDRA architecture shown in Fig 1A (Gyori *et al*, 2017). In particular, each source of information yields many mechanistic fragments that capture only a subset of the underlying process, often at different levels of abstraction. For example, one source might describe the MEK1 (HUGO name *MAP2K1*) phosphorylation of ERK2 (*MAPK1*) on a specific threonine residue (T185), whereas another source might describe the same process at the protein family level, stating that MEK phosphorylates ERK, without mentioning a specific isoform, residue or site position (Fig 1B). Individual mechanisms obtained from machine reading are not only fragmented but they also include different types of technical errors that must be overcome (Fig 1B, red font). One familiar analogy to the process of assembling mechanistic fragments into useful knowledge is the assembly of a genome sequence from many noisy, overlapping sequencing reads (Fig 1B). The goal of knowledge assembly is similarly to achieve the best "consensus" representation of the underlying processes, incorporating as much mechanistic detail as possible while minimizing errors. Ultimately, the process is expected to yield computational approaches for finding truly missing or discrepant information, by analogy with variant calling.

When attempting to scale the process of assembly from curated natural language to scientific publications, we identified multiple technical and conceptual problems that needed to be addressed to assemble coherent knowledge at scale. These included (i) inconsistent use of identifiers for biological entities among different sources, (ii) full or partial redundancy between representations of the same mechanisms, and (iii) technical errors in named entity recognition and relation extraction. Such problems are particularly salient when integrating literature-mined interactions, but they also exist when aggregating interactions from multiple curated databases, due to differences in curation practices. For example, in Pathway Commons v12 there are at least eight different curated representations of the process by which *MAP2K1* phosphorylates *MAPK1*, each at a different level of detail (Fig EV1A). We developed a set of INDRA algorithms for addressing each of these assembly challenges. These algorithms are general-purpose and can be configured into custom assembly pipelines (Fig 1C) to support a wide range of modeling applications, as illustrated in the following examples of machine reading, assembly, and data analysis (Box 1).

INDRA integrates mechanisms from pathway databases and machine reading

We used six machine reading systems, Reach (Valenzuela-Escárcega *et al*, 2018), Sparser (McDonald *et al*, 2016), MedScan (Novichkova *et al*, 2003), TRIPS/DRUM (Allen *et al*, 2015), RLIMS-P (Torii *et al*, 2015), and the ISI/AMR system (Garg *et al*, 2016) to process 567,507 articles (using full-text content when available, and allowed by copyright restrictions, and abstracts otherwise; Table 1) curated as having relevance to human protein function (see the "Article corpus for event extraction" section of Materials and Methods). Reader output was normalized to INDRA Statements (see "INDRA Statement representation" in Materials and Methods), yielding ~$5.9{\cdot}10^6$ unassembled or "raw" Statements (Fig 2A). Readers differed in the types of relations they extracted: Reach, Sparser, MedScan, and TRIPS/DRUM produced a multitude of different INDRA Statement types (each between 19 and 28 different types, depending on the reader) while RLIMS-P is limited to extracting Phosphorylation Statements, and ISI/AMR to Complex Statements. Overall, readers extracted 31 different Statement types (Table EV1). These were combined with approximately $7.3{\cdot}10^5$ INDRA Statements extracted from structured sources such as Pathway Commons and the BEL Large Corpus; this used previously described extraction logic (a means of converting structured information of different types into INDRA Statements; Gyori *et al*, 2017) but extended to multiple additional sources including SIGNOR (Perfetto *et al*, 2016). In combination, reading and databases yielded a total of ~$6.7{\cdot}10^6$ raw Statements. We then processed these raw Statements using an assembly process as described below and illustrated schematically in Fig 2A. In what follows, we refer to the resulting set of assembled INDRA Statements as the *INDRA Benchmark Corpus*.

After collecting information from each source, a pipeline involving a series of normalization and filtering procedures was applied (green and red boxes, respectively, in Fig 2A). These processing steps are also available as individual and reusable software modules in INDRA. First, we removed Statements that were supported by mentions indicative of a hypothesis rather than an assertion (for instance, including sentences phrased as "we tested whether…"). Next, "grounding mapping" was performed to correct systematic errors in named entity normalization, which often arise due to the ambiguity of biomedical naming conventions. INDRA integrates a manually curated mapping table that fixes those entities frequently misidentified by reading systems (described in detail in Bachman *et al* (2018)) and a set of machine-learned models that perform disambiguation based on text context (by integrating the Adeft (Steppi *et al*, 2020) and Gilda (Gyori *et al*, 2022) systems). "ER" is an example of a common but ambiguous entity: it can stand for endoplasmic reticulum, estrogen receptor, estradiol receptor, emergency room, and a variety of other entities and concepts depending on context. As currently implemented, Reach, Sparser, and other reading systems ground "ER" deterministically to a single identifier (e.g., estrogen receptor) irrespective of context. In contrast, the machine-learned disambiguation models integrated into INDRA predict the most likely meaning of entities such as ER based on surrounding text; this is then used to correct the results of text reading systems.

The next step of the grounding mapping process normalizes identifiers for individual entities using a network of cross-references between equivalent identifiers in different namespaces (Fig EV2A).

© 2023 The Authors

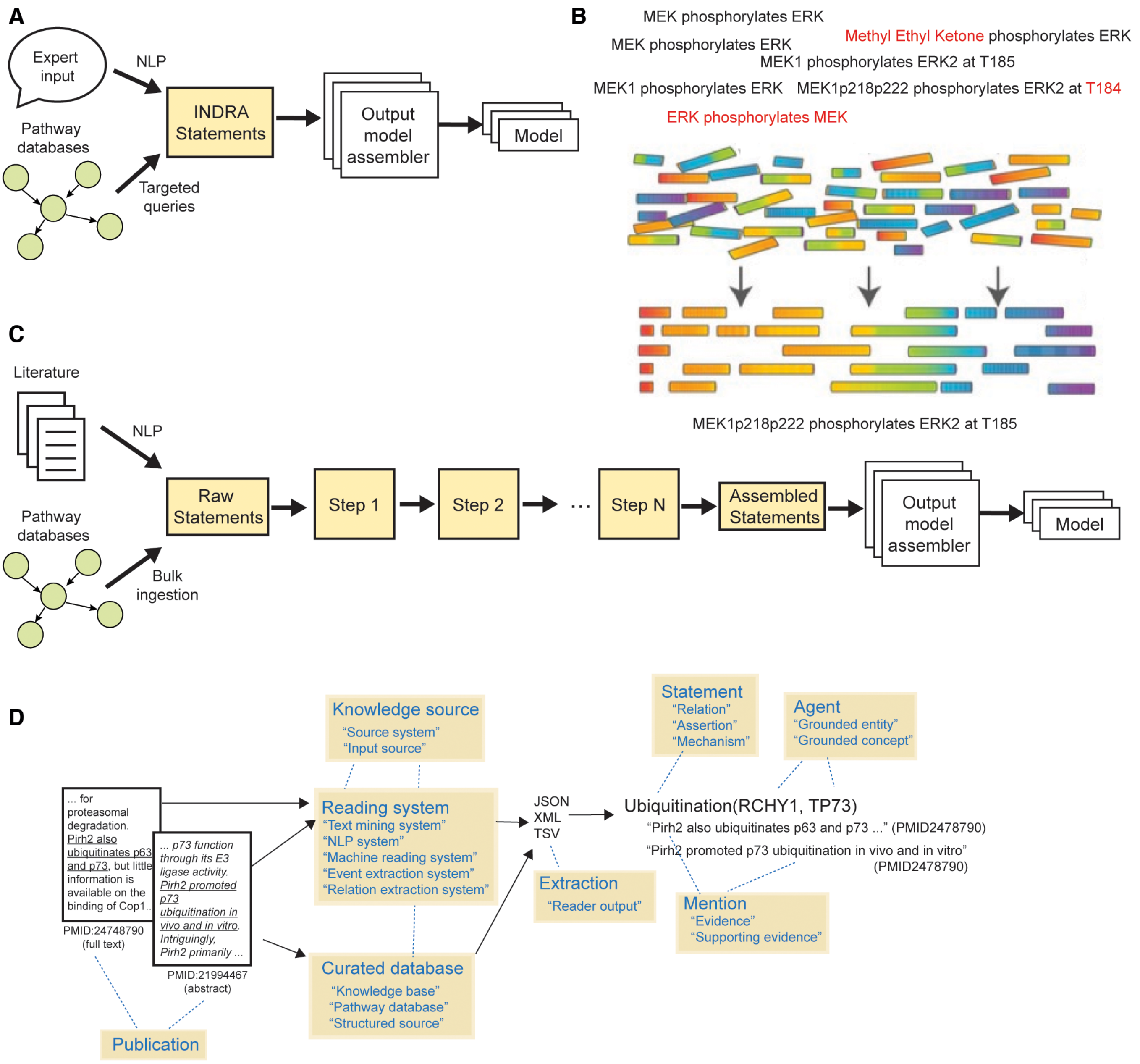

Figure 1. Conceptual overview of knowledge assembly.

A Assembly of models from diverse knowledge sources. Structured (pathway databases) and unstructured (literature, expert input in natural language) biological knowledge is converted into machine-readable, mechanistic fragments. These fragments must be assembled into a coherent corpus before the generation of specific models for data analysis.

B Mechanistic "fragments" capture incomplete but overlapping aspects of an underlying molecular mechanism (here, the phosphorylation of ERK by MEK). Fragments may also contain errors (highlighted in red). Assembly involves identifying relationships between fragments to arrive at a consensus representation that captures available information.

C Artifacts involved in the collection of mechanisms from knowledge sources by INDRA, and their representation as INDRA Statements. Yellow boxes show key terminology used to refer to different artifacts with additional synonyms provided in quotes.

D INDRA knowledge assembly transforms raw statements into assembled statements from which models can be generated. The individual steps of the assembly pipeline (Steps 1 to *N*, yellow background) operate on INDRA Statements and are configurable from a library of built-in or user-defined functions.

This addresses the opposite problem from the one described above (i.e., one name corresponding to multiple entities), namely that a single entity can have multiple identifiers in different namespaces, and these identifiers can be assigned inconsistently across machine reading systems and curated database sources. For example, a metabolite such as prostaglandin E-2 identified using a Chemical

 © 2023 The Authors

Box 1. Representing knowledge captured from multiple sources in INDRA (Fig 1D).

Scientific *publications* contain descriptions of mechanisms (interaction, regulation, etc.) involving biological entities. These descriptions can be extracted either by human experts and stored in *curated databases* or by automated *reading systems* using natural language processing. Collectively, reading systems and curated databases serve as *knowledge sources* for INDRA. *Extractions* from knowledge sources are made available to INDRA in a variety of custom machine-readable formats such as JSON, XML, and TSV. INDRA processes such extractions into a standardized representation, a set of INDRA *Statements*. Each *Statement* represents a type of mechanism (e.g., Ubiquitination), and has multiple elements, including *Agents* representing biological entities such as proteins or small molecules, and potentially also mechanistic detail such as an amino acid residue for a modification. Each *Statement* can be supported by one or more *mentions*, each representing a single curated database entry or a single extraction by a reading system from a sentence in a given publication. Mentions are represented by INDRA as Evidence objects that have a multitude of properties representing rich provenance for each mention, including the source sentence and the identifiers of the source publication.

Table 1. Distribution of content types for literature corpus.

Content type	Count	Percentage
PubMed abstract	384,628	67.8%
Elsevier	81,567	14.4%
PMC open access	74,654	13.2%
PMC author's manuscript	25,950	4.6%
Missing	707	0.1%

Entities of Biological Interest identifier (ChEBI; Hastings *et al*, 2016) will be assigned additional equivalent identifiers and a standard name so that it has the same canonical form as an equivalent metabolite identified using an NCBI Medical Subject Heading identifier (MESH; Fig EV2A and B). This procedure ensures that Agents in INDRA Statements take on canonical identifiers in multiple namespaces, irrespective of the identifier used in the original source of knowledge.

After these filtering and correction steps were performed, 38% of Statements contained Agents with ungrounded entities; these were filtered out. In examining entity texts corresponding to Agents that were ungrounded and therefore filtered out, we found that the most commonly occurring unnormalized Agent texts were generic, high-level terms such as "expression," "activity," "cytokine," "growth," and "signaling" consistent with our previous finding that ungrounded entities often correspond to generic concepts lacking context (Bachman *et al*, 2018). Because the current study focuses on biology involving human genes, we also filtered Statements to include only those containing human genes and their families or multi-protein complexes. Each of these processing and filtering steps operates at the level of individual Statements and changes both the overall number of Statements and the proportion of Statements in the corpus from each input source, as shown in Fig 2B.

The final normalization procedure we performed was sequence position normalization. This accounts for inconsistencies in attributed sequence positions of post-translational modifications, some of which involve outright errors in residue numbers, while others involve the implicit, interchangeable use of residue numbers between human and model organism reference sequences (preprint: Bachman *et al*, 2022). Human and mouse residue numbers are also used interchangeably in many papers even though residue numbering in orthologous proteins frequently differs. In all cases, a method for sequence normalization is necessary for accurate knowledge assembly. After normalization, we filtered out Statements containing non-canonical sequence positions (about 1% of all Statements) as these likely arose from machine reading errors. This yielded a final corpus of ~$2.9 \cdot 10^6$ filtered and normalized Statements.

We then used INDRA to combine Statements representing equivalent mechanisms from different sources into a single unique Statement; each unique Statement was associated with the supporting mentions from all contributing knowledge sources including curated databases and reading systems (Fig 2C). In some cases, multiple readers will have extracted the same mechanisms from the same sentence, but different reading systems often generated mentions supporting a specific Statement from different sentences in given publications or even from different publications (Fig 2C). This highlights the substantial differences between reading systems and the benefits of the multi-reader approach used in this paper. For the Benchmark Corpus, ~$2.9 \cdot 10^6$ filtered Statements yielded ~$9 \cdot 10^5$ unique Statements after combining duplicates (Fig 2A), with an average of ~3 supporting mentions per Statement. However, the distribution of mentions per Statement was highly non-uniform, with a large number of Statements (63%) attributable to a single sentence or database entry, and a small number of Statements (82 in total) having > 1,000 supporting mentions (Fig 2D). For example, the Statement that "TP53 binds MDM2" has 2,494 distinct pieces of evidence. Although the data are noisy for high mention counts, the distribution of mentions per Statement appears linear on a log–log plot (Fig 2D) implying a long-tailed distribution potentially following a power law. To confirm this, we fitted the observed mention distribution using two approaches: (i) linear regression of the complement cumulative distribution of mention counts on a log scale, which showed a strong linear relationship ($r^2 = 0.999$, $P < 10^{-17}$), and implied a power law exponent of $\alpha = 2.33$ and (ii) fitting directly to a power law using the *powerlaw* software package (Alstott *et al*, 2014), which showed that the distribution was fit by a power law with exponent $\alpha = 2.38$ (standard error $\sigma = 0.008$; Fig 2E) and was more likely than alternative distributions such as exponential ($P < 10^{-38}$) or positive log-normal ($P < 10^{-30}$). Thus, the distribution of Statements having a given number of supporting mentions is similar to long-tailed distributions observed in a variety of domains including linguistics, computer networking, and demographics (Clauset *et al*, 2009).

A significant benefit of jointly assembling mechanisms from both databases and literature is that curated interactions from databases become linked to textual evidence that supports the interaction (Fig 2F). For example, the fact that RCHY1 ubiquitinates TP73 appears as a curated interaction in the NCI-PID database (Schaefer *et al*, 2009) with reference to PMID20615966 (Sayan *et al*, 2010), but without providing specific supporting text within that publication. In the Benchmark Corpus, INDRA aligns seven mentions

© 2023 The Authors

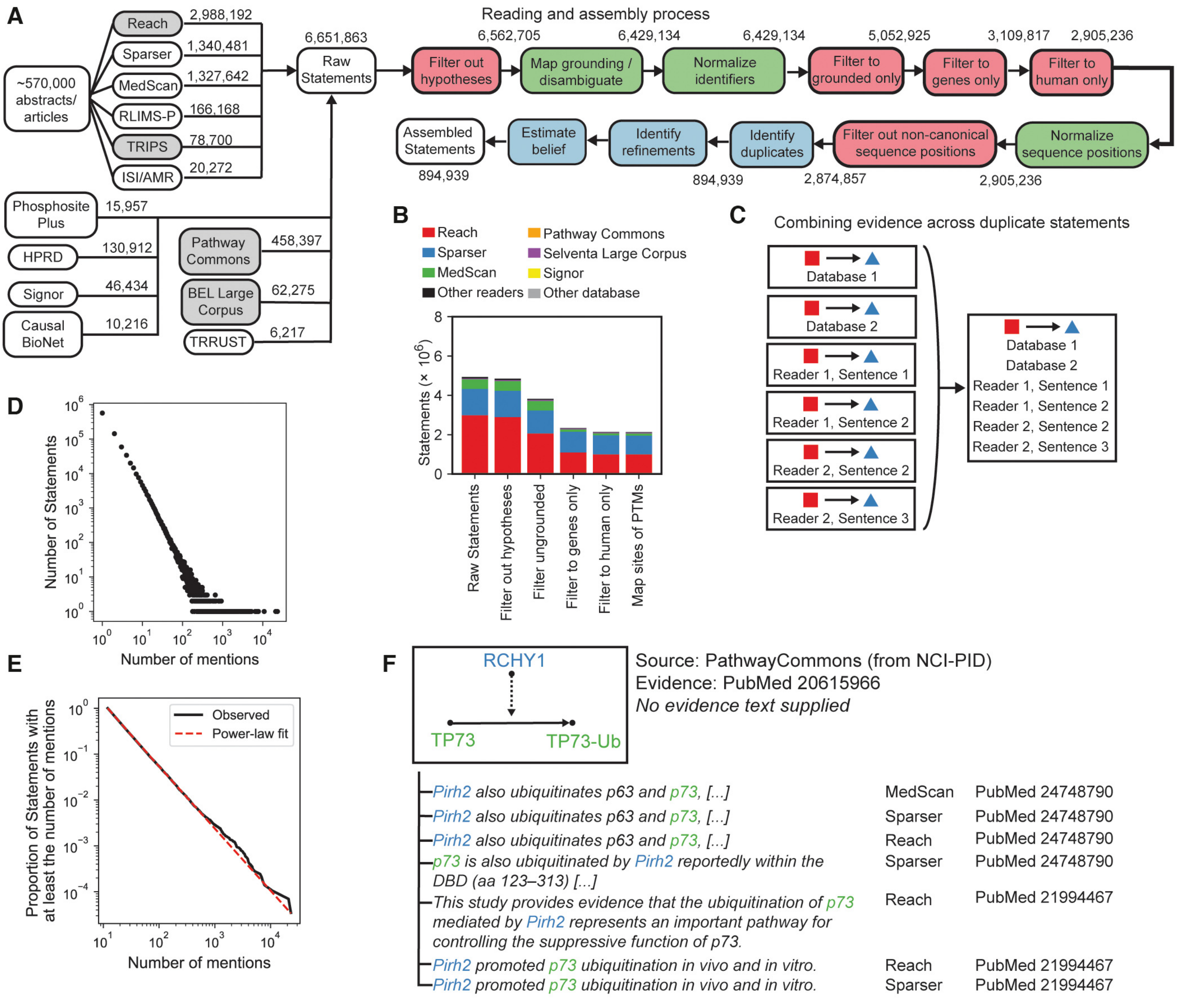

Figure 2. The INDRA knowledge assembly pipeline was used to create a Benchmark Corpus.

A The INDRA assembly pipeline for the Benchmark Corpus. The pipeline starts with ~570 thousand publications processed by multiple reading systems, as well as structured database sources including Pathway Commons and SIGNOR. Raw Statements extracted from these sources proceed through filtering (red), normalization (green), and assembly (blue) steps. Gray shading on input modules indicates modules that were originally introduced in Gyori *et al* (2017).

B Number of INDRA Statements, by source, at key stages of the assembly pipeline shown in panel (A).

C Combining duplicate Statements. INDRA identifies raw Statements that are identical and creates a single unique Statement with all of the associated mentions.

D Distribution of mention counts (including both mentions in text and database entries) across all Statements in the Benchmark Corpus. Each point in the scatterplot represents the number of Statements with a given number of mentions.

E Complement cumulative distribution of Statements as a function of the number of mentions supporting them (black) and the maximum likelihood estimate of a power-law fit to the distribution (red).

F Assembly of Statements enriches curated mechanisms in pathway databases with literature evidence from text mining. Here, a reaction in Pathway Commons represents the ubiquitination of TP73 (p73) by the ubiquitin ligase RCHY1 (Pirh2). Reach, Sparser, and MedScan each extract statements matching the one from Pathway Commons and provide references to PubMed identifiers and specific evidence sentences as provenance.

obtained from text mining with the ubiquitination of TP73 by RCHY1 and these are derived from four sentences found in two more recent publications (Wu *et al*, 2011, 2; Coppari *et al*, 2014; Fig 2F). Such aggregation of evidence across curated databases and text-mining systems is highly beneficial because it increases our confidence in the accuracy and relevance of a mechanism (Kemper *et al*, 2010). In these cases, INDRA, due to its automated nature, provides a substantial advantage for linking literature sources to specific interactions compared to comparable manual curation, which would be laborious and time consuming.

 © 2023 The Authors

Detecting hierarchical relationships between mechanisms

Following processing, filtering, and the identification of duplicate Statements it is necessary to identify relationships among "overlapping" Statements (Fig 3A). A pair of Statements is considered to be overlapping when one functions as a refinement (i.e., adds additional mechanistic detail) to the other. Although the analogy in this case is not perfect, something similar is required in

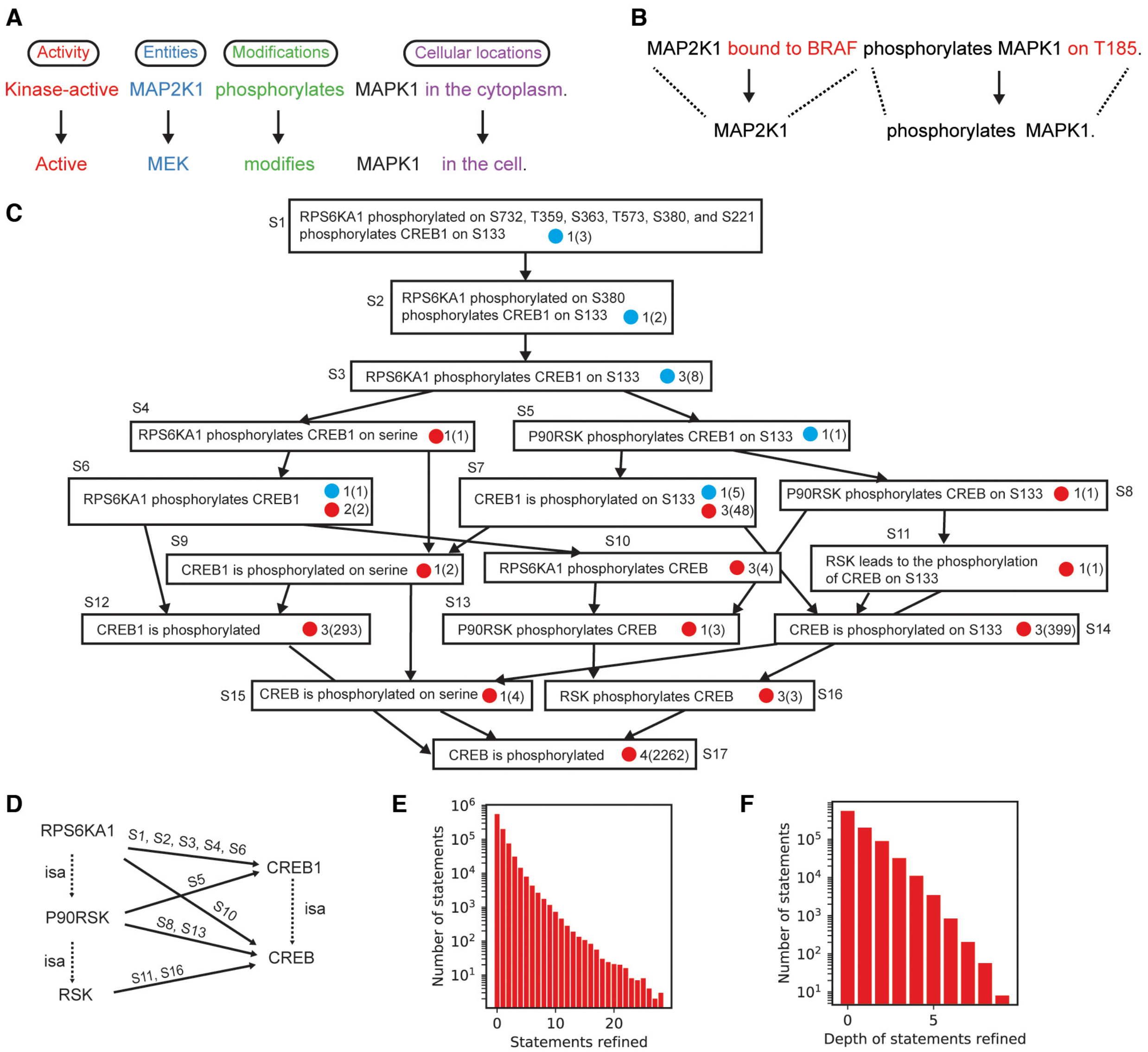

Figure 3. Identifying refinement relationships among Statements.

A Refinement by hierarchies of Statement elements as defined by INDRA. The two Statements shown contain the same number and types of information but all elements in the top Statement are refinements of the corresponding elements in the bottom Statement according to the INDRA Statement hierarchies.

B Refinement by additional context. The upper Statement contains all information in the lower one but also provides additional detail, making it a refinement of the one below.

C Example refinement graph for a Statement from the example corpus. For clarity, the transitive reduction of the hierarchy is shown, and each Statement object is displayed via its English language equivalent. Each node in the graph represents a statement with blue or red circles representing evidence from pathway databases or mentions extracted by machine reading systems, respectively. Next to each blue or red circle, the number of different sources is shown with the overall number of mentions from these sources in parentheses. For example, the statement "CREB1 is phosphorylated on S133" has five pieces of evidence from one pathway database source, and 48 mentions extracted by three reading systems. Edges represent refinement relationships and point from more specific to less specific Statements.

D Graph of family relationships (dotted *isa* edges) and Statements representing phosphorylation (solid edges, annotated with Statement identifiers from panel C), between different levels of specificity of the RSK and CREB protein families.

E Number of Statements based on the total number of other Statements that they refine.

F Number of Statements with different depths of Statements that they refine (i.e., the length of the longest path in the graph of refinement relations starting with the given Statement).

© 2023 The Authors

genome assembly—if a shorter sequence is fully contained in a longer sequence, the shorter one is redundant. When such a relationship exists between two Statements, we say that the more detailed one "refines" the less detailed one. Refinement can happen at the level of entities (e.g., one Agent representing a protein family and another Agent a specific member of that family), or molecular states and context (e.g., an explicit reference to a site of post-translational modification in one Statement and its omission in another). The refinement relationship between Statements is determined using a partial ordering logic that compares pairs of Statements based on their individual elements (where elements include the Agents involved in the Statement, and, depending on the type of Statement, post-translational modifications, cellular locations, types of molecular activity, etc.) and determines whether each element is either equivalent to or a refinement of the other (Fig 3A). To accomplish this, INDRA makes use of hierarchies of each relevant type of element, including proteins and their families and complexes drawn from FamPlex (Bachman *et al*, 2018), combined with chemical and bioprocess taxonomies from ChEBI and the Gene Ontology (Ashburner *et al*, 2000; e.g., *MAP2K1* is a specific gene in the MEK family, Fig 3A, blue), protein activity types (e.g., kinase activity is a specific type of molecular activity, Fig 3A, red), post-translational modifications (e.g., phosphorylation is a type of modification, Fig 3A, green), and cellular locations (also obtained from the Gene Ontology; e.g., that the cytoplasm is a compartment of the cell, Fig 3A, purple). A Statement is also considered a refinement of another if it contains additional contextual details but is otherwise a match across corresponding elements. One example of such a refinement relationship is shown in Fig 3B, in which the first Statement (Fig 3B, top) describes an additional molecular state (*MAP2K1* being bound to *BRAF*) and mechanistic detail (T185 as the specific site of modification of *MAPK1*) over another Statement (Fig 3B, bottom) which omits these contextual details.

Pairwise refinement of relationships among Statements is most easily represented using a graph in which nodes represent Statements and directed edges point from a node representing a Statement to another node representing the Statement that it refines. Such Statement refinement graphs can be quite deep (i.e., the length of a directed path starting from a Statement can consist of a large number of edges going through many refined Statements). For example, the refinement subgraph for *RPS6KA1 phosphorylated on S732*, *T359*, *S363*, *T573*, *S380*, and *S221 phosphorylates CREB1 on S133* (Fig 3C, where *RPS6KA1* encodes the ribosomal S6 kinase and *CREB1* a transcription factor) has nine levels. The refinement relationships for this Statement reveal the varying levels of specificity at which a given mechanism is described in sources: *CREB is phosphorylated* has 2,268 mentions in the literature collected by four reading systems, *RPS6KA1 phosphorylates CREB1* has three mentions in total from both literature and curated databases, and *CREB1 is phosphorylated on S133* has 399 mentions. It is also worth noting that support from curated databases for these Statements (Fig 3C, blue circles) is not attributable to a single database source. For example, the Statement labeled S1 in Fig 3C is derived only from Pathway Commons, S5 only from SIGNOR, and S7 only from HPRD (Mishra, 2006). Thus, human-curated databases are individually incomplete and mutually inconsistent with respect to the way they report specific mechanisms and the literature they cite as supporting evidence.

Organizing Statements hierarchically helps to ensure that an assembled model does not contain information that is mechanistically redundant. For instance, when the Statements in Fig 3C are viewed as a graph with nodes representing entities (RPS6KA1, CREB1, etc.) and edges representing phosphorylation reactions (Fig 3D, solid arrows), five partially redundant edges can be identified (e.g., RPS6KA1 → CREB1, P90RSK → CREB1, and P90RKS → CREB) connecting members of the RSK and CREB protein families at different levels of specificity (e.g., P90RSK is a member of the RSK family, Fig 3D, dashed arrows). A key feature of INDRA is that it can recover Statement refinement relationships, enabling principled resolution of complex redundancies, for example, by retaining only Statements that are not refined by any other Statements (in the case of Fig 3C, the Statement labeled as S1 at the top of the graph). The refinement graph in Fig 3C also reveals how a highly specific Statement can serve as evidence for all the other Statements it subsumes, a relationship that is exploited when estimating Statement reliability.

We found that refinement relationships were common in the Benchmark Corpus: 38% of Statements refined at least one other Statement, and some Statements refined a large number of other Statements, including 89 Statements that refined at least 20 other Statements (Fig 3E). These Statements are typically ones that represent a canonical (i.e., often described) mechanism (e.g., the mechanism by which members of the AKT protein family phosphorylate GSK3 proteins) at a high level of detail and subsume multiple variants of the same mechanism described at a lower level of detail. We also found that the Benchmark Corpus contained tens of thousands of refinements involving three or more levels (Fig 3F), emphasizing that many mechanisms across databases and literature are described at many levels of specificity. INDRA assembly can reconstruct these relationships and allow resolving the corresponding redundancy.

Modeling the reliability of INDRA Statements with the help of a curated corpus

One of the most challenging problems in using mechanisms generated by text mining is the unknown reliability of the extracted information. While the notion of "reliability" includes conventional scientific concerns, such as the strength of the evidence supporting a particular finding or study (Fig 4A, upper left quadrant), in practice the overwhelming majority of incorrect assertions result from *technical* errors in machine reading (Fig 4A, lower left quadrant). Common reading errors include systematic misidentification of named entities, incorrect polarity assignment (e.g., classifying activation as inhibition), failure to recognize negative evidence (e.g., "A *does not* cause B"), and difficulty distinguishing hypotheses from assertions and conclusions (e.g., "*we tested whether* A causes B" as opposed to "A causes B"; Valenzuela-Escárcega *et al*, 2018; Noriega-Atala *et al*, 2019). These errors arise primarily because scientific texts use a wide range of non-standard naming conventions to refer to genes, proteins, and other entities, as well as complex grammatical structures to convey the confidence associated with a result or data point. Indeed, much of the art in scientific writing is to generate text that appears to progress inexorably from a hypothesis to the description of supporting evidence to a conclusion and its caveats. This type of writing can be difficult even for humans to fully

 © 2023 The Authors

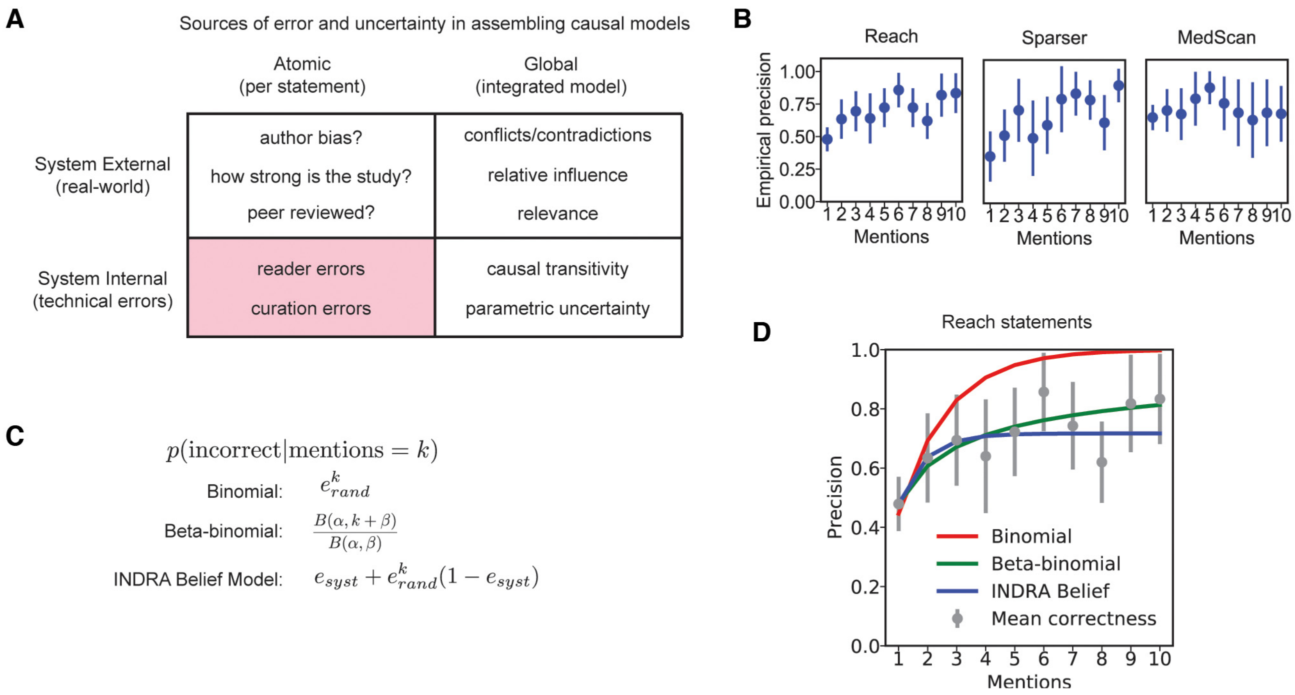

Figure 4. Estimating statement belief for a single machine reader.

A A classification of sources of error and uncertainty in assembling causal models. Sources are classified according to whether they are external or internal to the INDRA system, and whether they arise at the level of individual Statements (atomic) or an integrated network or model (global).

B Empirical precision of three reading systems based on the number of mentions supporting a given Statement extracted by that reader.

C Mathematical formulas for Statement correctness for three different Belief Models. Each model specifies the probability that a Statement is incorrect overall given that a specific number k of mentions support it from a given source. e_{rand}: random error for the source; e_{syst}: systematic error for the source; $B(\alpha, \beta)$: Beta function.

D Fits of the three belief models in (C) plotted against the empirical precision of Reach-extracted Statements.

understand. However, addressing the technical errors of reading systems at the level of individual Statements is a prerequisite for addressing the additional issues that arise when Statements are combined into causal models (Fig 4A, right quadrants). Additional challenges with integrated models include dealing with contradictions between Statements, assessing the relative influence or relevance of multiple Statements in a given context, as well as issues surrounding causal transitivity across multiple Statements.

To study the reliability of our assembled Statements, we sampled a set of Statements from the Benchmark Corpus. The sampled Statements had between 1 and 10 mentions per Statement and arose from five reading systems (Reach, Sparser, MedScan, RLIMS-P, and TRIPS; we excluded the ISI/AMR system from this analysis due to the low number of extractions it produced). Two of the authors, both of whom are PhD biomedical research scientists, used this to develop a Curated Corpus from the sampled Statements. Curation involved determining whether a given mention correctly supported a specific Statement based on human understanding of the sentence containing the mention and the overall context of the publication. Statements were sampled by mention count in a stratified manner to establish the relationship between mention count and reliability; high mention-count Statements are therefore overrepresented relative to their baseline frequency in the Benchmark Corpus (see "Statement Curation" section of Materials and Methods). The resulting data set covers 1,800 Statements with a combined total of 6,022 unique mentions a subset of which (see Table 2 and "Statement Curation" section of Materials and Methods) was used to assess the individual technical reliability of reading systems.

For a single reading system, the reliability of an extracted Statement has been observed to increase with the number of different supporting mentions (Valenzuela-Escárcega *et al*, 2018). We hypothesized that a Statement with multiple mentions would be more reliable if the mentions had been independently extracted by more than one reading system. To test this idea, we used two complementary approaches to create models of Statement reliability: (i) structured probability models that build on empirical error characteristics of individual reading systems based on the Curated Corpus and (ii) machine learning (ML) models trained on the Curated Corpus. Structured probability models require much less training data, but, given sufficient training data, machine-learned models are generally more expressive and likely to be more accurate in predicting Statement reliability.

Modeling the reliability of Statements from individual reading systems

We first examined the error characteristics of *individual* reading systems. For individual readers, analysis of the Curated Corpus showed that while Statements with more mentions are generally more reliable, in many cases Statements supported by many sentences were

Table 2. Summary of Statement curation dataset for the purposes of single reader belief assessment.

Reader	Mention curation	1	2	3	4	5	6	7	8	9	10
Reach	Complete	57 (119)	26 (41)	25 (36)	16 (25)	26 (36)	24 (28)	26 (35)	31 (50)	18 (22)	20 (24)
	Incomplete	57 (119)	26 (41)	25 (36)	16 (25)	26 (36)	24 (28)	26 (36)	31 (50)	18 (22)	20 (24)
RLIMS-P	Complete	87 (109)	24 (26)	23 (25)	10 (10)	6 (6)	6 (6)	6 (6)	6 (6)	7 (7)	25 (25)
	Incomplete	87 (109)	24 (26)	23 (25)	10 (10)	10 (10)	11 (11)	12 (12)	11 (11)	12 (12)	25 (25)
TRIPS	Complete	158 (199)	46 (51)	28 (29)	3 (3)	7 (7)	12 (12)	24 (26)	12 (13)	9 (11)	9 (9)
	Incomplete	158 (199)	46 (51)	28 (29)	9 (11)	10 (10)	12 (12)	24 (26)	12 (13)	9 (11)	9 (10)
Sparser	Complete	9 (25)	13 (25)	9 (13)	6 (12)	11 (19)	6 (8)	16 (19)	2 (3)	3 (7)	11 (12)
	Incomplete	9 (25)	13 (25)	10 (14)	6 (12)	12 (20)	8 (10)	16 (19)	23 (29)	13 (21)	19 (21)
MedScan	Complete	63 (96)	22 (31)	4 (6)	0 (0)	2 (2)	0 (0)	0 (0)	0 (0)	0 (0)	13 (19)
	Incomplete	63 (96)	22 (31)	15 (22)	12 (15)	23 (26)	13 (17)	9 (13)	7 (11)	9 (13)	13 (19)

Entries are formatted as "number correct (total curated)." Each column shows the number of mentions (between 1 and 10) supporting a given Statement in the curation dataset. Rows marked as "Complete" show counts only for Statements for which all mentions were curated while "Incomplete" also includes Statements where less than the total number of mentions was curated. Counts across readers are not unique; if a Statement has mentions from multiple readers, it is counted in multiple rows.

still incorrect due to the presence of systematic errors (Fig 4B). For example, the Sparser reading system extracted the Statement *MAOA binds MAOB* with 10 mentions from 10 different publications, but all extractions were incorrect because the system incorrectly interpreted "association" as referring to a physical interaction rather than a statistical association between MAOA and MAOB (monoamine oxidase A and B), which is what the original publications described. We compared three alternative probability models for their ability to capture the dependence of sentence reliability on mention count: (i) a simple binomial model, (ii) a beta-binomial model (a binomial model in which the probability of success at each trial follows a beta distribution), and (iii) a two-parameter model that captures both random and systematic errors—we termed this latter model the *INDRA Belief Model* (Fig 4C; see "The INDRA Belief Model" section of Materials and Methods). Parameters for each of the three models were estimated from the data from the Curated Corpus using Markov chain Monte–Carlo (MCMC; see "Parameter estimation for INDRA Belief, Binomial, and Beta-Binomial Models" section of Materials and Methods). Both the beta-binomial model and the *INDRA Belief Model* outperformed the binomial model at predicting Statement correctness from mention counts, primarily due to their ability to capture the empirical observation that even high-mention Statements do not approach an accuracy of 100% (a phenomenon accounted for by modeling systematic reader errors; Fig 4D, Table 3). The *INDRA Belief Model* performed slightly better than the beta-binomial model at predicting Statement correctness for both the Reach and Sparser reading systems (Table 3) due to its better fit to low mention-count Statements (Fig 4D, mentions 1, 2, and 3). An additional advantage of the *INDRA Belief Model* is that the random and systematic error rates e_{rand} and e_{syst} are interpretable and can be estimated heuristically by examining a small number of high-mention Statements (with precision approximately equal to e_{syst}) and 1-mention Statements (with precision equal to $e_{syst} + (1 - e_{syst})e_{rand}$). This makes it possible to set reasonable parameters for the INDRA Belief Model based on prior intuition or examination of a small number of exemplary Statements. Since the *INDRA Belief Model* performed the best overall, it is used as the default model in INDRA when no curation data are available. However, the beta-binomial model more accurately fit the underlying distribution of correct mentions for each Statement, suggesting that further research is needed on such error models (Fig EV3).

Table 3. Maximum likelihood values for alternative belief models using best-fit parameters (lower values indicate a better fit).

Model	Reach, −log (Max likelihood)	Sparser, −log (Max likelihood)	MedScan, −log(Max likelihood)
Binomial (1 param)	375.4	104.7	150.1
Beta-binomial (2 params)	260.2	90.7	97.4
INDRA Belief Model (2 params)	259.5	91.2	96.9

Multi-reader overlap is associated with higher Statement frequency and reliability

To better understand the potential for *multi*-reader reliability assessment, we characterized the extent of reader overlap in the Benchmark Corpus (i.e., when two or more readers produce mentions supporting the same Statement). We found that 19% of assembled Statements had supporting mentions from two or more reading systems (Table 4; Figs 5A and EV4A), but the bulk of Statements was

Table 4. Frequencies of relations in the corpus by the total number of sources.

Num. readers	Freq. (%)
1	81.3%
2	14.42%
3	3.55%
4	0.67%
5	0.05%

 © 2023 The Authors

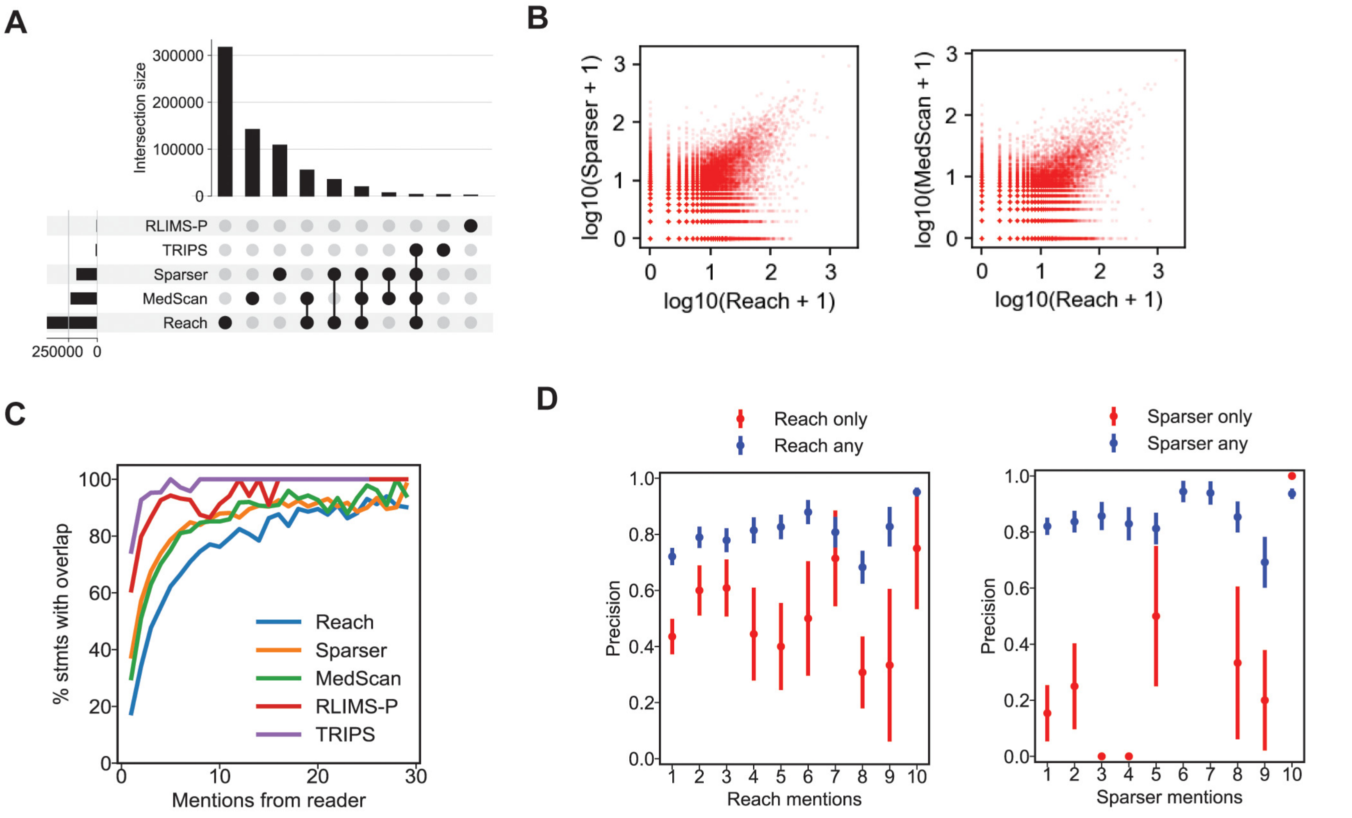

Figure 5. Estimating Statement belief with multiple machine readers combined.

A Upset plot (equivalent to a Venn diagram with more than three sets) of Statement support for five machine reading systems integrated by INDRA. For a given Statement, two or more readers intersect if they each provide supporting mentions for it. The top 10 subsets are shown; for a full upset plot of all subsets, see Fig EV4A.

B Number of mentions from Reach and Sparser (left) and Reach and MedScan (right) for a given Statement, each Statement being represented by a red dot. Mention counts are plotted on a logarithmic scale.

C The percentage of Statements for which an intersection (i.e., any overlap) between reading systems is observed as a function of the number mentions from a given reader; the data are plotted separately for each of the five reading systems.

D Empirical Statement precision as a function of the number of mentions from Reach (left) and Sparser (right), plotting the cases for which *only* Reach or Sparser provides supporting mentions for a Statement (red) and the case where all Statements are taken into account (blue).

supported exclusively by either Reach, Sparser, or MedScan (Fig 5A). The low overlap between readers is attributable to differences in their design, including their approaches to grammatical parsing, named entity recognition, associated resources (i.e., which lexical sources each reader incorporates), and the types of grammatical or semantic patterns that can be recognized. Low overlap among readers implies that using multiple reading systems in an integrated fashion via INDRA can increase coverage relative to any single reading system.

Despite the relatively small overlap among readers, the number of mentions from each reader supporting a Statement showed substantial correlation, with both $\rho(Reach, Sparser)$ and $\rho(Reach, MedScan) > 0.6$ (Table 5). We found, however, that these correlations in mention counts among reading systems were primarily driven by a subset of relations with very high numbers of mentions (Fig 5B). More generally, we found that reader overlaps for a Statement increases as a function of the number of supporting mentions an *individual* reader extracted for the Statement (Fig 5C). Overall, these data support the observation that, if a mechanism represented by a Statement is described in many different sentences across input documents, multiple systems are likely to extract supporting mentions, and these will often come from different sentences and publications (as we showed in Fig 2C and E).

Table 5. Correlations between reader mention counts.

	Sparser	MedScan	RLIMS-P	TRIPS
Reach	0.611	0.633	0.072	0.374
Sparser		0.454	0.114	0.420
MedScan			0.034	0.338
RLIMS-P				0.096

When we examined the relationship between reader overlap and Statement correctness using the Curated Corpus, we found that Statements supported by many mentions were more likely to

overlap with other readers and be correct (Fig EV4B, blue points along diagonals). Notably, in the case of Reach, the reader for which the most extensive subset of curated Statements was generated, we found that the probability of Statement correctness increased with the overall number of Reach mentions, but only for high-mention Statements that also included support from other readers (Fig 5D, blue points). For relations with support *only* from Reach, empirical correctness increased from 1 to 2 mentions (an observation consistent with the findings regarding the Reach system's precision (Valenzuela-Escárcega *et al*, 2018)), but additional Reach-only mentions were not associated with substantial further increases in precision (Fig 5D, red points). Thus, in a multi-reader setting, the *absence* of reader overlap also plays a key role in assessing Statement reliability. These observations imply that combining multiple reading systems can be highly valuable when assessing Statement correctness based on supporting mentions. It also provides information that can be used by developers of reading systems to increase recall and precision.

To characterize systematic issues affecting multiple readers, we also examined sentences associated with Statements that were incorrectly extracted by more than one reader. Recurring errors included misgrounding due to overlapping aliases (e.g., grounding —by four readers—of "TPP1" to gene TPP1 rather than gene ACD for which "TPP1" is an alias), incorrect extraction of negative results (e.g., "our preliminary attempts have not identified direct phosphorylation of PPARγ by MST2," extracted by three readers as a Phosphorylation statement in which MST2 modified PPARγ), unrelated subclauses being causally linked (e.g., "quiescent cells attenuate eIF2α phosphorylation and induction of the ER stress proapoptotic gene GADD153" incorrectly extracted by three readers as a phosphorylation of GADD153 by eIF2α), incomplete named entity recognition (e.g., "Shc associates with epidermal growth factor (EGF) receptor," incorrectly extracted by two readers as binding between Shc and EGF, not EGFR), and extraction of protein–DNA binding as protein–protein binding (phrases similar to "c-Jun binds to AP-1 sites to regulate gene expression" incorrectly extracted by four readers as binding between c-Jun and the AP-1 complex, which includes c-Jun as a component). In many of these cases, human readers are able to recognize subtleties in the language that are difficult for machines to parse correctly.

Two approaches to modeling the reliability of Statements from multiple readers

We evaluated two strategies for assessing the reliability of Statements using mention counts from multiple readers: (i) extending the INDRA Belief Model and (ii) training machine learning models on the Curated Corpus. Even though reader errors were not fully independent of each other (Fig EV4B), we assumed independence between different reading systems (Zhang, 2004) to extend the INDRA Belief Model while adding the fewest additional model parameters. Specifically, we adjusted how the model formulated error estimates to express the probability that all mentions extracted by the readers were *jointly* incorrect (see "The INDRA Belief Model" section of Materials and Methods). We also assessed how well the extended INDRA Belief Model could predict Statement correctness based on mention counts per reading system compared to several different machine-learned classifiers. These classifiers included Logistic Regression on log-transformed mention counts, k-Nearest Neighbors, support vector classifiers, and Random Forests (see the "Machine-learned models of Statement reliability" of Materials and Methods). Models were compared based on the area under the precision-recall curve (AUPRC), which is a more robust metric for class-imbalanced data (~73% of Statements in our curated corpus were correct) than the area under the receiver-operator curve (AUROC). In interpreting the AUPRC values, note that the curated corpus is, by construction, biased toward Statements with higher mention counts and, therefore, greater reader overlap. For example, Statements supported by only a single reader constitute 81% of the Benchmark corpus (Table 4) but only 35% of the curated corpus (see "Statement Curation" section of Materials and Methods). As such, reported AUPRCs should be interpreted as a measure of the *relative* performance of each model across Statements supported by different combinations of readers rather than measures of general performance.

We found that, when mention counts were the only input feature, the INDRA Belief Model yielded the greatest AUPRC, followed by the Logistic Regression and Random Forest models (Table EV2, rows 1, 3, and 2, respectively). However, machine learning models outperformed the INDRA Belief Model when they were extended to use additional Statement features, such as the Statement type, the number of supporting articles (i.e., the number of distinct publications from which mentions were extracted), the average length of the mention texts (longer sentences were more likely to be incorrectly interpreted), and the presence of the word "promoter" in the sentence (a frequent indicator that a sentence describing a protein to DNA promoter interaction had been mis-extracted as a PPI; Table EV2, rows 8–13; see the "Encoding of features for Statement belief prediction" section of Materials and Methods). This implies that—as long as sufficient training data are available—machine-learned classifiers can use additional Statement-associated features to boost performance relative to the INDRA Belief Model which relies solely on mention counts.

Additionally, since INDRA can identify refinement relationships among Statements (Fig 3), mentions can be combined across different levels of detail for use in reliability estimation. For example, evidence supporting the specific Statement, "MAP2K1 phosphorylates MAPK1 on T185," also supports the more generic Statement, "MEK phosphorylates ERK." Combining these refining mentions improved precision and recall: the AUPRC of the Random Forest model increased from 0.893 to 0.913 when using only mention counts (Table EV2, row 2 vs. 17), and from 0.932 to 0.937 when using all features (Table EV2, row 9 vs. 24). Further when we incorporated overlapping mentions from curated databases as features alongside reader mentions, we found that the Random Forest model's AUPRC increased to 0.942—the highest AUPRC reached across all models and conditions. Because mentions from more specific Statements flow to more general ones but not the reverse, the belief estimates for the most specific Statements are determined only by their *directly* supporting evidence. This leads to an overall inverse relationship between specificity and belief that allows Statements to be filtered to the most specific statement lying above a certain threshold of belief, thereby excluding potentially unreliable and highly specific Statements in which extracted details may reflect technical errors rather than meaningful additional context.

Because readers perform differently on the same input text, Statements supported by multiple readers are less common than

 © 2023 The Authors

Statements supported by a single reader, but our analysis showed that both the existence of reader overlap as well as lack of overlap for a given Statement can be informative in predicting Statement correctness. Moreover, in the absence of human-curated data across multiple Statement features—a type of data that is laborious to generate—a parametric model (such as the INDRA Belief Model) based on the error profiles of individual readers can perform well from a precision-recall perspective. When sufficient curated training data are available, machine learning models such as Random Forests can achieve greater performance, obtaining the highest AUPRCs in several different configurations. These findings provide empirical support for INDRA's approach to assembling sets of Statements from multiple text mining and curated database sources with principled estimates of correctness. Both the INDRA Belief Model and the machine-learned classifier models are available in the *belief* submodule of INDRA and allow parameters to be either manually set or estimated from curation data.

Validation of assembled mechanisms and comparison against curated resources

To test INDRA on a prototypical biocuration task, we compared the subset of Statements representing human PPIs in the Benchmark Corpus to the BioGRID database (Oughtred *et al*, 2019). BioGRID is a curated public database containing structured information on protein–protein and protein–small molecule interactions, as well as genetic interactions obtained from multiple organisms. These interactions were extracted by expert curators from a combination of high-throughput datasets and focused studies. As a measure of the utility of INDRA for biocuration, we determined (i) the number of previously-uncurated PPIs that the INDRA Benchmark Corpus could add to BioGRID and (ii) the amount of new literature evidence that it could add to PPIs currently in BioGRID. We used our best-performing Random Forest model to assign a belief to each INDRA Statement in the Benchmark Corpus.

The Benchmark Corpus contained ~26,000 Statements representing PPIs already in BioGRID and ~101,000 PPIs that were absent (Fig 6A); the latter potentially represent known but previously uncurated interactions. By grouping all PPIs in bins defined by belief score, we found that belief score was highly correlated with whether a PPI was curated in BioGRID (Fig 6B). This provides a quantitative corroboration of the belief scores and, by extension, suggests that a substantial number of the PPIs absent from BioGRID involve reading errors that are associated with low belief scores. The belief scores obtained from the Random Forest model can be interpreted as calibrated probabilities of correctness, allowing belief scores to estimate the *number* of correct Statements in each bin. The proportion of Statements in BioGRID was consistently below the belief score for the bin, suggesting that each bin contained correctly extracted but uncurated PPIs (Fig 6B, blue line below diagonal). Assuming that all Statements found in BioGRID were correctly extracted, we estimated a lower bound of 28,600 correct but uncurated PPIs in the Benchmark Corpus, a 6% increase over the ~480,000 unique human PPIs in BioGRID.

As a practical matter, extending a curated resource such as BioGRID would logically involve focusing first on Statements with the highest belief scores. The ~2,200 uncurated Statements with belief scores > 0.9 would be expected to yield > 1,870 PPIs or roughly six correct for every seven reviewed. Statements with lower belief scores are more numerous but also have a lower expected yield: 18,700 correct Statements would be expected among the 41,600 uncurated Statements with belief scores between 0.4 and 0.9, with the curation yield starting at 67% (for Statements with belief between 0.8 and 0.9) to 29% (for Statements with belief between 0.4 and 0.5) (Fig 6C). To illustrate this, we examined one PPI not currently in BioGRID that involved binding of the KIF1C kinesin to RAB6A, a GTPase and regulator of membrane trafficking. INDRA assembled a total of 40 mentions supporting this PPI, extracted by two machine reading systems (Reach and

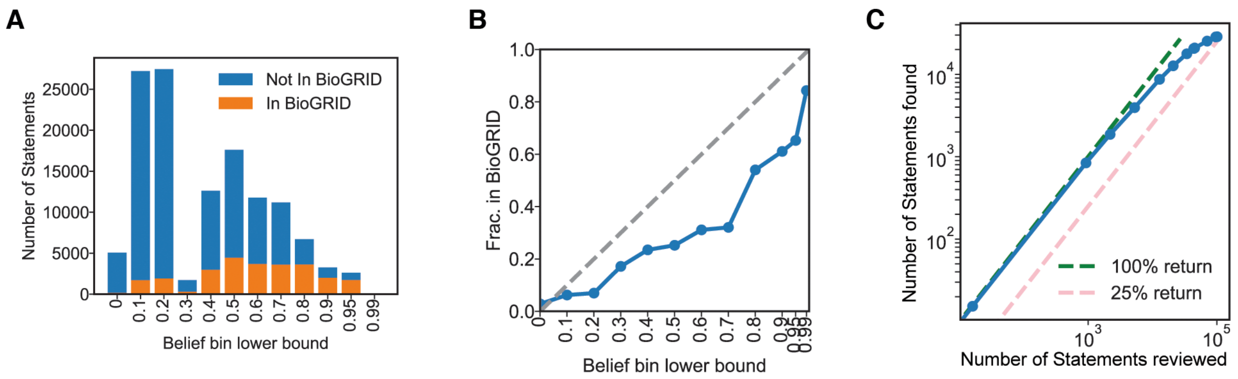

Figure 6. Comparison of INDRA-assembled mechanisms with a curated resource, BioGRID.

A Number of INDRA Statements representing PPIs (i.e., complex formation between two human proteins) grouped into bins by their belief score (as determined by a random forest belief model), differentiating whether the PPI represented by the Statement appears in BioGRID (orange) or not (blue).

B Fraction of INDRA Statements representing PPIs that appear in BioGRID grouped into bins by their belief score. A gray dashed line shows the expected fraction of correct Statements in each belief bin. The space between the gray and blue lines (i.e., between the expected fraction of correct Statements in each bin and the fraction of Statements that appear in BioGRID) represents an estimate of the set of correct Statements missing from BioGRID.

C Plot showing estimated curation yield if Statements were reviewed by decreasing belief score for inclusion into a curated resource. The blue line plots the number of correct Statements expected to be found as a function of the number of Statements reviewed, with green and pink dashed lines serving as guides showing 100% return (i.e., every reviewed Statement is correct) and 25% return (i.e., 1 out of 4 reviewed Statements is correct).

© 2023 The Authors

Sparser), into a Statement with a belief score of 0.82. Human curation confirmed that the interaction had been reliably demonstrated using both co-immunoprecipitation and reconstitution experiments (Lee *et al*, 2015).

A second application of INDRA is to add evidence for PPIs already in BioGRID and thereby (i) provide different types of evidence for an existing PPI (e.g., mass spectrometry vs. 2-hybrid interaction), (ii) reveal additional biological settings or cell types in which a PPI might occur, and (iii) provide additional mechanistic detail about a particular PPI. As an example of (i) and (ii), BioGRID lists only three publications as a reference for the interaction between brain-derived neurotrophic factor (BDNF) and the NTRK2 receptor tyrosine kinase, whereas the INDRA Benchmark Corpus contains 168 mentions of this interaction from a total of 94 publications. Some of these additional publications provide primary experimental evidence for this interaction (e.g., Vermehren-Schmaedick *et al*, 2014) whereas Wang *et al* (2009a) discuss the role of the BDNF-NTRK2 interaction in important clinical settings. As an example of (iii), the interaction between paxillin (PXN) and the tyrosine kinase PTK2B is supported by six references in BioGRID; INDRA not only identified 49 mentions from 18 different publications supporting this PPI but assembled a Statement with substantially more mechanistic information than BioGRID: namely that PTK2B, when phosphorylated on Y402, phosphorylates PXN on Y118 (Park *et al*, 2006; Moody *et al*, 2012, 2). This example shows that for a PPI lacking mechanistic detail, INDRA can illuminate the directionality and type of regulation, as well as the amino acids involved in post-translational modifications.

Detecting and explaining gene dependency correlations with an assembled causal network

To study how networks that incorporate text-mined information can aid in the interpretation of functional genomic datasets, we used INDRA to detect and explain significant gene dependencies in the Cancer Dependency Map (https://depmap.org; Meyers *et al*, 2017; Tsherniak *et al*, 2017). The DepMap reports the effects of RNAi or CRISPR-Cas9 mediated gene inactivation on cell viability and growth in > 700 cancer cell lines using a competition assay. In this assay, the effect of gene inactivation is assessed by determining the rate at which a specific knockout (or knockdown) disappears from a co-culture comprising cells transfected with a genome-scale RNAi or CRISPR-Cas9 library. It has previously been observed that genes whose knockouts have similar effects on viability across a large number of cell lines—a phenomenon known as codependency—frequently participate in the same protein complex or pathway (Meyers *et al*, 2017; Tsherniak *et al*, 2017; Pan *et al*, 2018; Doherty *et al*, 2021; Rahman *et al*, 2021; Shimada *et al*, 2021). For example, CHEK2 and CDKN1A have a correlation coefficient of 0.359 and 0.375 in DepMap CRISPR and RNAi data, respectively (Fig 7A), and this codependency can be explained by the fact that the CHEK2 kinase is an activator of CDKN1A (also known as p21) and that the two genes jointly regulate cell cycle progression. To obtain robust measures of gene co-dependencies, we combined the CRISPR and RNAi perturbation data by converting the Pearson correlation coefficients for each gene pair into signed z-scores and computing the combined z-score between the two datasets using Stouffer's method (Fig 7A). In analyzing the data, we first accounted for a bias also observed by others (Dempster *et al*, 2019; Rahman *et al*, 2021), namely that many of the strongest correlations are between mitochondrial genes (Fig 7B). These correlations have been described as an artifact of the screening method (such as the timepoint of the viability measurements relative to cell doubling time) rather than reflecting true co-dependencies (Rahman *et al*, 2021). We considered the correlations among these genes to be "explained" *a priori* due to their shared mitochondrial function. Using the mitochondrial gene database MitoCarta as a reference (Rath *et al*, 2021), we excluded correlations among them from subsequent analysis.

From the Benchmark Corpus of assembled INDRA Statements, we generated a network model in which each node represents a human gene and each directed edge corresponds to an INDRA Statement (such as Phosphorylation, Activation, etc.) connecting two nodes. We used the resulting network for two tasks: first, to constrain the number of hypotheses tested when determining the statistical significance of codependency correlations and second, to find mechanistic explanations for the observed codependencies.

For the first task, we calculated the number of codependencies that were significant at a false discovery rate (FDR) of 0.05 using three methods for controlling FDR with and without the use of the network to limit the number of hypotheses tested (Table 6). Overall, fewer codependencies were significant when we restricted comparisons to relationships in the INDRA-assembled network, both because the network is incomplete and because many codependencies reflect indirect functional relationships (which are not captured by a single direct edge in the network). However, many codependencies (4,007 using Benjamini–Yekutieli FDR correction) were detected as significant *only* when using the network (Table 6, "INDRA only") due to the smaller number of hypotheses tested. Moreover, the majority of these (2,729) were based on interactions obtained only from machine reading, of which > 60% were supported by a Statement with a belief score greater than 0.5.

Conversely, the existence of a codependency added context to text-mined mechanisms. For example, the negative correlation between ERBB2 and STMN1 ($\rho = -0.146$ in DepMap CRISPR data) was associated with a single INDRA phosphorylation Statement in the Benchmark Corpus; the fact that the codependency correlation is *negative* indicates that ERBB2 phosphorylation of STMN1 inhibits it (a finding corroborated by Benseddik *et al* (2013)). Similarly, the negative correlation between GRB10 and IRS2 ($\rho = -0.137$ in CRISPR) is consistent with reports that the binding of GRB10 to IRS2 is inhibitory. This provides context for the INDRA Statement derived from (Mori *et al*, 2005; Keegan *et al*, 2018, 1) that "GRB10 binds IRS2" and is particularly interesting because the effect of GRB10 binding to IRS2 has been reported as both inhibitory (Wick *et al*, 2003) and activating (Deng *et al*, 2003, 10). The negative DepMap correlation suggests that the inhibitory effect is more relevant in the context of the two genes' co-regulation of cell viability. Overall, these findings suggest that an INDRA-assembled network can lead to the detection of codependencies that would otherwise be missed, and—as the previous two examples show—the combined information from data and assembled knowledge provides deeper mechanistic insight into each interaction than data alone.

We next tested whether the Benchmark Corpus could provide mechanistic explanations of DepMap codependencies beyond what can be explained by curated pathway databases. We considered

 © 2023 The Authors

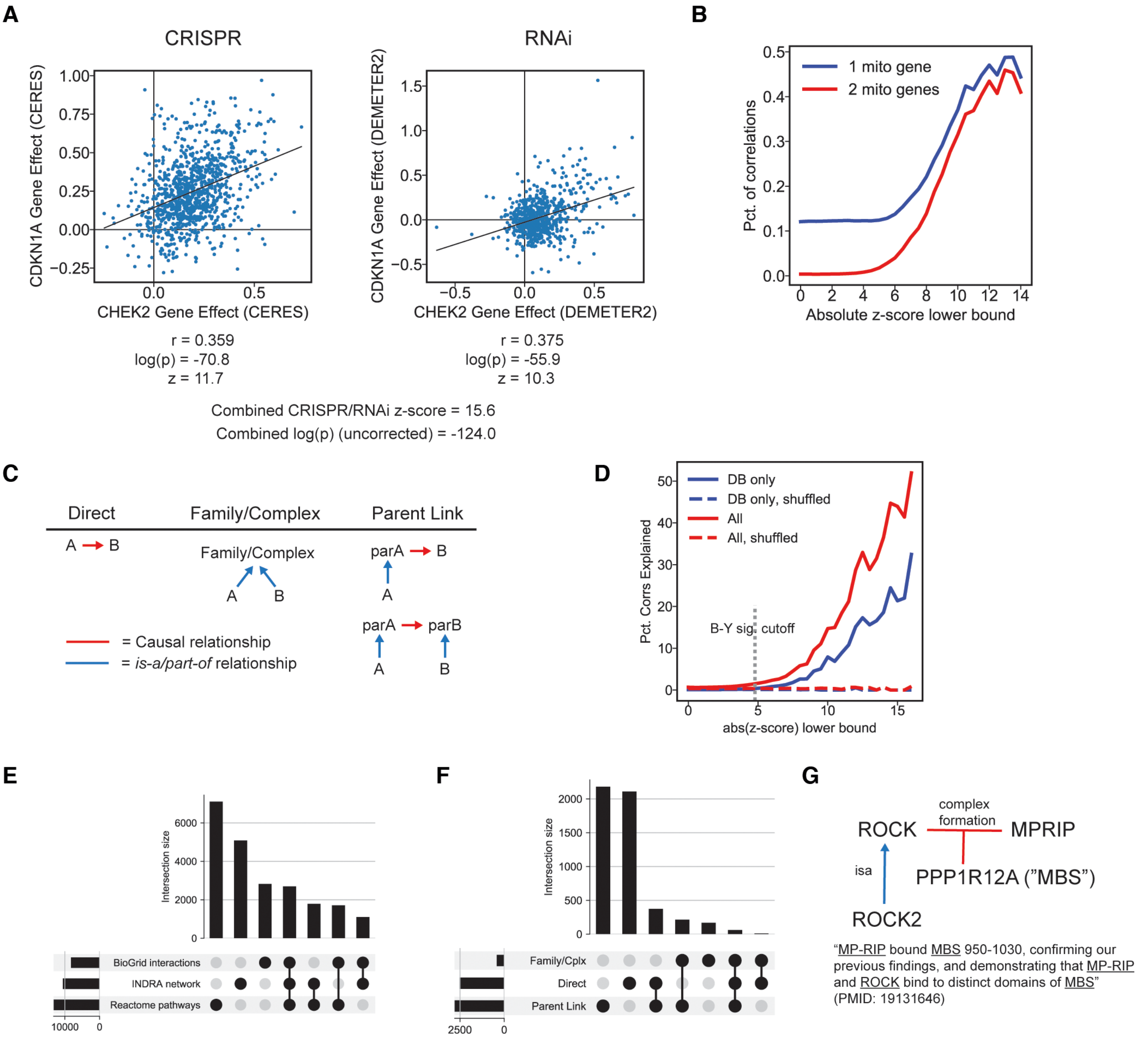

Figure 7. Detecting and explaining gene codependency in cancer cell lines using an INDRA-assembled network.

A CRISPR (left) and RNAi (right) data from DepMap showing the codependency of the CHEK2 and CDKN1A genes across a panel of cancer cell lines (each blue dot represents a cell line, placed according to normalized cell viability change upon gene perturbation). Black lines show the linear regression plot over the cell line viability values.

B Percent of gene codependencies (i.e., correlations) involving one or two mitochondrial genes as a function of the absolute z-score corresponding to the codependency.

C Patterns of network nodes and edges that constitute an "explanation" for an observed DepMap codependency, including "Direct" (a direct edge between two specific genes A and B), "Family/Complex" (two genes A and B are part of the same family or complex), and "Parent Link" (where one or both of the specific genes A and B are related via a parent family/complex they are part of).

D Percent of codependencies/correlations explained using the INDRA network when considering all edges (red) or only edges supported by curated databases, excluding text mining (blue), with randomly shuffled controls shown.

E Upset plot showing the intersection of explanations for DepMap codependencies provided by three networks: BioGRID interactions, the INDRA network, and Reactome pathways.

F Upset plot showing the intersection of three types of explanation for DepMap codependencies provided by the INDRA Network, corresponding to explanation patterns shown in panel (C).

G An example explanation for the codependency between ROCK2 and MPRIP derived from the INDRA network. INDRA provides evidence for a complex in which ROCK (the protein family of which ROCK2 is a member) binds MPRIP in a three-way complex with PPP1R12A (also called MBS) through the mention shown at the bottom (extracted from Wang *et al*, 2009a; Wang *et al*, 2009b).

Table 6. Number of codependencies detected at a significance cutoff of $P < 0.05$ without multiple hypothesis correction or after one of three methods for multiple hypothesis testing correction (Bonferroni, Benjamini–Hochberg, and Benjamini–Yekutieli).

		INDRA prior	
	No prior	Total	INDRA only
Number of comparisons (non-mitochondrial)	121,778,711	265,874	N/A[a]
Correlations with uncorrected $P < 0.05$	21,526,511	63,926[a]	N/A[a]
Significant corrs after Bonferroni	99,544	4,982	1,836
Significant corrs after Benjamini–Hochberg	5,025,535	30,127	7,506
Significant corrs after Benjamini–Yekutieli	972,831	12,812	4,007

Results are shown for a case in which no prior is used and data are analyzed directly ("No prior"), or when an INDRA prior is used ("INDRA prior/Total"). The rightmost column shows the number of novel codependencies recovered exclusively when an INDRA prior was used along with correction for multiple testing ("INDRA prior/INDRA only").
[a]Figures for uncorrected *P*-values do not apply to the "INDRA prior/INDRA-only" case because without correction for multiple testing, the prior does not play a role in determining significance. Figures are shown for the "INDRA prior/Total" case to establish the number of codependencies with uncorrected *P*-values > 0.05 that fall within the scope of the INDRA network; this serves as an upper bound for the number of correlations determined to be significant with the different approaches to multiple testing shown in the bottom three rows.

three types of explanatory relationships: (i) direct causal relationships where one gene was reported to regulate, modify or interact with another, for example, the inhibition of TP53 by MDM2 (Fig 7C, "Direct"), (ii) information that the two correlated genes were members of the same protein family or complex, as indicated by FamPlex relations (Bachman *et al*, 2018; Fig 7C, "Family/Complex"), or (iii) a link between the parent family/complex of a gene and another gene or its parent family/complex (Fig 7C, "Parent Link"). For the purpose of this analysis, we did not consider multi-step causal paths between genes in the network to be explanatory. This is due to the challenge of ensuring that sequences of edges in the network represent causally linked biochemical events rather than unrelated associations, which would lead to false-positive explanations. Capturing and representing information about causal transitivity in biological networks is the subject of ongoing research (Fig 4A, "causal transitivity" in the lower-right quadrant).

To measure the impact of text mining, we derived a smaller, "database-only" network from the Benchmark Corpus by excluding edges that were supported *only* by text mining evidence from the "full" network. As a control, we permuted the node labels of both the full and database-only networks and repeated our analysis. We found that the full network explained a greater proportion of codependencies than the database-only network (22 vs. 11% for codependencies with |z-score| > 6), with similar improvements at all significance levels (Fig 7D). This improvement is striking considering the text mining results were drawn from a corpus that constitutes only a fraction of what is currently available in PubMed. We also found that for either network, stronger codependencies were more likely to be explainable than weaker ones (Fig 7D), highlighting that the curated and published mechanistic knowledge (that is likely to be picked up by INDRA) is generally biased toward the most robust functional relationships.

To better characterize how INDRA-assembled networks provide mechanistic context for relationships in DepMap, we compared codependencies explainable via the full INDRA network to those explainable via interactions in BioGRID or by co-membership in a Reactome pathway. Of the 345,077 non-mitochondrial gene pairs with DepMap codependency correlations above the Benjamini–Yekutieli significance cutoff, only 21,475, or 6.2%, could be explained by BioGRID interactions, a common Reactome pathway, or the INDRA network, highlighting the many potential functional relationships in DepMap without a literature precedent. The largest number of explanations were based on co-occurrence in a common Reactome pathway, the least specific type of explanation; 6,952 codependencies were explainable only via this information (Fig 7E). The INDRA network accounted for the next-highest number of unique explanations with 4,819 (Fig 7E). Interestingly, a majority of these were regulatory relationships mediated by protein families and complexes, to which the codependent genes belong (Fig 7F, "Parent Link" explanations). While less stringent than explicit gene–gene relationships, family-mediated connections provide compelling explanations for genes commonly described at the family or complex level (Bachman *et al*, 2018). For example, the strong negative correlation between MPRIP and ROCK2 (ρ = −0.291) is explained by multiple text-mined Statements that link MPRIP to the ROCK protein family (referred to generically as "ROCK" or "Rho kinase") via their joint binding to the myosin-binding subunit of the myosin light chain phosphatase (gene PPP1R12A, Fig 7G; Surks *et al*, 2003; Wang *et al*, 2009b; Nunes *et al*, 2010).

The remainder of the INDRA-dependent explanations were derived from Statements involving two specific codependent genes (Fig 7F, "A– > B"). While these explanations are "direct" in the sense that two genes are linked by an edge in the INDRA network, the relationships may not involve physical binding and might therefore have intermediaries (a mechanistically indirect connection). Such indirect mechanisms can be an advantage in many systematic explanation tasks. For example, the strong correlation between BRAF and MITF (ρ = 0.456) cannot be explained by a common Reactome pathway, a physical interaction in BioGRID, or interactions in any of the pathway databases incorporated in the INDRA network. However, BRAF and MITF are linked by an INDRA network edge derived from 20 text-mined Statements (supported by 59 distinct mentions), which characterize their complex mutual regulatory relationship. The Statements correctly capture the evidence that *oncogenic* BRAF *activates* the expression of MITF through the transcription factor BRN2 (Kumar *et al*, 2014) whereas *wild-type* BRAF in melanocytes *inhibits* MITF expression due to the lack of expression of BRN2 (Wellbrock *et al*, 2008). Because INDRA can represent molecular states on Agents (in this case BRAF vs. its mutated form BRAF-V600E), these extracted Statements can provide machine-readable information that differentiates the two distinct contexts. Finally, we noted that interactions obtained exclusively from text mining were not restricted to well-characterized or indirect relationships: for example, the INDRA network also incorporates a Statement extracted from a single sentence explaining the correlation between DOCK5 and BCAR1 (better known as p130Cas) as arising from their joint interaction with the scaffold protein CRK (Frank

 © 2023 The Authors

et al, 2017, 5). Despite their robust correlation (ρ = 0.361), DOCK5 and BCAR1/p130Cas have only been co-mentioned in a total of three publications in PubMed.

Discussion

In this paper, we described a method, implemented in the INDRA software system, for robust, automated assembly of mechanistic causal knowledge about biological interactions. The method normalizes information from heterogeneous sources, including both curated databases and text mining systems, identifies relationships between Statements, and uses statistical models to estimate the reliability of each Statement based on the compendium of supporting evidence. The corpus used in this paper (~570,000 articles) focuses on human gene function and covers only a fraction of the published biomedical literature (> 30 million articles). Nevertheless, we demonstrate that it is possible to meaningfully extend curated interaction databases and provide explanations for gene dependency correlations in the Cancer Dependency Map. INDRA enriches biocuration and data analysis efforts in three ways, (i) by aggregating and normalizing new, previously uncurated mechanisms directly from the literature in machine-readable form, (ii) by adding mechanistic detail (activation, modification, binding, etc.) to generic PPIs or empirical relationships, and (iii) by supplying supporting evidence and context from the literature. Others can use INDRA tools since they are open-source (https://github.com/sorgerlab/indra) and well-documented (https://indra.readthedocs.io). INDRA has already been used for diverse knowledge assembly, curation, and analysis tasks—using custom pipelines similar to the one used in the current paper—including network-based gene function enrichment (Ietswaart et al, 2021), causal analysis of viral pathogenesis (Zucker et al, 2021), drug target prioritization for acute myeloid leukemia (Wooten et al, 2021), assembling knowledge about protein kinases (preprint: Moret et al, 2021), assisting manual biocuration efforts (Hoyt et al, 2019a; Glavaški & Velicki, 2021; Ostaszewski et al, 2021), and helping authors capture mechanistic findings in computable form (Wong et al, 2021).

The approaches to knowledge assembly described here are related to prior work on the integration of biological databases (Türei et al, 2016; Rodchenkov et al, 2020; Szklarczyk et al, 2021), assembly of biological knowledge graphs (Himmelstein et al, 2017; preprint: Hoyt et al, 2019b), large-scale biomedical event extraction (Van Landeghem et al, 2011), and estimation of the reliability of individual interactions in knowledge graphs (preprint: Neil et al, 2018; Jia et al, 2019). However, the current work goes beyond the straightforward aggregation of interactions from multiple sources by (i) systematically normalizing named entities, (ii) organizing Statements by specificity, and (iii) exploiting information about Statement sources, frequency, and specificity to predict Statement reliability. Others have introduced innovative methods for using machine reading and curated databases for automated model learning and extension, while also using INDRA to process reader output (Holtzapple et al, 2020) and estimate Statement reliability (Ahmed et al, 2021). However, we believe our work to be the first demonstration of a method that automatically assembles reliable, non-redundant mechanistic knowledge from both curated resources and multiple biomedical text mining systems at scale.

As used in this work INDRA focuses on capturing the types of information typically represented in biological pathway databases: post-translational modifications and physical and regulatory interactions among proteins, chemicals, and biological processes. It does not currently represent genetic interactions, gene–disease relationships, biomarkers, or other types of statistical associations. However, given suitable data sources and text extraction systems, INDRA could be used for named entity linking, hierarchical assembly, and reliability assessment for a wide range of other types of knowledge. Indeed, the core methodology described here has been used to generate probabilistic causal models from a reading system that extracts causal relations from open-domain text (Sharp et al, 2019).

Automatically assembled knowledge bases have many uses in computational biology beyond the biocuration and functional genomics use cases we described here. For example, methods have been described that use pathway information for regularization in machine learning (Sokolov et al, 2016), to control false discovery in hypothesis testing (Babur et al, 2015), and to generate causal hypotheses from -omics data (Tuncbag et al, 2016; Dugourd et al, 2021). Most current methods for prior-guided data analysis require information about mechanisms to be "flattened" into directed (in some cases signed) networks (as we ourselves did for DepMap gene codependency analysis). However, INDRA has the ability to assemble information from multiple sources while preserving much more detailed information about mutations, modifications, and activity states. This supports the further development of analytical methods that exploit prior knowledge that is both broad and mechanistically detailed. INDRA facilitates this because it assembles information from sources in terms of knowledge-level assertions rather than model-specific implementations. Thus, different types of causal models can be generated from the same assembled knowledge depending on the downstream application, including signed directed graphs, Boolean networks, or other types of executable models. In our previous work, we described a method for automatically assembling declarative natural language into detailed ODE-based models of biological pathways (Gyori et al, 2017). In principle, the methods described here also allow mechanistically detailed signaling models to be initialized from systematically compiled knowledge bases with a quality suitable for static causal analysis (Gyori et al, 2021, see https://emmaa.indra.bio). However, manual curation is generally still required to produce dynamical simulation models, due to the need to supply reverse rates and guarantee detailed balance; making this process more efficient is an area of ongoing research (Gyori & Bachman, 2021).

One of the striking conclusions from this work is that different reading systems extract different types of information from the same text corpus. Moreover, even in the case of a single INDRA Statement, different reading systems extract different mentions from the same text. This points to the value of using multiple readers in parallel, something that has not been previously explored, and suggests that direct comparison of reading system errors has the potential to improve these systems. To make use of multiple readers we developed an approach for estimating the technical reliability of Statements based on the number of mentions, the characteristics of their supporting evidence, and the properties of individual reading systems. While addressing this purely technical source of

© 2023 The Authors

uncertainty is a prerequisite for the practical use of text-mined information in downstream applications, addressing additional types of uncertainty in assembled knowledge and models is a worthwhile area of future research. In particular, there is a need for systematic approaches to managing conflicts and contradictions among assembled Statements (Fig 4A, upper right), which often take the form of polarity conflicts ("A activates B" vs. "A inhibits B"). While polarity conflicts can arise due to systematic errors in machine reading (Noriega-Atala *et al*, 2019), many represent inconsistent reports in the underlying scientific literature. These conflicts can potentially be addressed by more thorough incorporation of biological context alongside causal information (Noriega-Atala *et al*, 2020), for example, through the use of functional data such as the DepMap, or potentially by ensemble modeling procedures that capture polarity uncertainty in downstream analysis. Another concern in the use of text-mined information is the unreliability of many scientific studies (Baker, 2016). Recent efforts in meta-scientific analysis have examined features such as journal impact factors, article citations, and collaboration networks among researchers to determine whether these can predict the likelihood of the future replication of a study (Danchev *et al*, 2019). Large-scale assembly of causal information from the literature using INDRA has the potential to facilitate the study of the experimental, computational, and meta-scientific factors that promote scientific reproducibility and its absence.

It is interesting to speculate what might be possible were all of PubMed to be made fully machine readable. The corpus of 570,000 papers used in this study was chosen in part to focus on human genes and their functions. Because it is not a randomly selected subset of all 30 million PubMed articles, comprehensive machine reading followed by assembly in INDRA is unlikely to generate 60-fold more mechanistic information than the current study. To obtain a rough estimate of what could be expected, we determined the increase in the number of unique Statements and total mentions for a single gene of interest, BRAF, obtainable by processing all machine-readable abstracts and full-text articles in PubMed with two readers, Reach and Sparser. We found that, relative to the Benchmark Corpus, unique Statements roughly doubled (from ~1,500 to ~3,300), while total mentions tripled (~4,000 to ~12,000) and the total number of supporting articles quadrupled (~1,000 to ~4,000). These numbers highlight the potential value of applying knowledge extraction and assembly methods more broadly, although accomplishing this will require overcoming legal restrictions to scale reading the literature (Westergaard *et al*, 2018), which extends even to papers in PubMed Central.

The availability of INDRA tools for combining the outputs of multiple text reading systems, assigning belief scores, and assembling fragmentary information into coherent and useful mechanistic knowledge has the potential to substantially impact biocuration and functional genomics. However, substantial technical issues must still be overcome for this promise to be realized. In particular, additional work is required to improve the precision and recall of text reading systems, address issues with grounding multi-component, misspelled, or ambiguous biological entities, extending Statements to additional types of mechanisms, and capturing biological context in a principled manner. INDRA represents a flexible and performative software environment to undertake this additional research.

Materials and Methods

Reagents and Tools table

Software	Reference or source
INDRA	https://github.com/sorgerlab/indra
ProtMapper	https://github.com/indralab/protmapper
Adeft	https://github.com/indralab/adeft
Gilda	https://github.com/indralab/gilda
FamPlex	https://github.com/sorgerlab/famplex
Reach	https://github.com/clulab/reach
Sparser	https://github.com/ddmcdonald/sparser
TRIPS/DRUM	https://github.com/wdebeaum/drum
RLIMS-P	https://research.bioinformatics.udel.edu/rlimsp/
MedScan	https://doi.org/10.1093/bioinformatics/btg207
ISI/AMR	https://hub.docker.com/r/sahilgar/bigmechisi
powerlaw	https://github.com/jeffalstott/powerlaw
emcee	https://github.com/dfm/emcee

Methods and Protocols

Article corpus for event extraction

The Entrez gene database was queried with the official gene symbols for all human genes in the HUGO database for MEDLINE articles curated as having relevance to the function of each gene. The resulting list of PubMed identifiers (PMIDs) is included in the code and data associated with the paper at https://github.com/sorgerlab/indra_assembly_paper. For these PMIDs, we obtained full-text content when available from three sources: The PubMed Central open access and author's manuscript collections, and the Elsevier Text and Data mining API (https://dev.elsevier.com). For the remaining PMIDs, we obtained abstracts from PubMed. Table 1 shows the distribution of text content sources.

Text mining of article corpus

We used multiple text mining systems integrated with INDRA to process all or part of the corpus of interest described in the previous section. INDRA implements an input API and processor dedicated to each reading system. Each such processor takes as input the reading system's extractions for one or more articles, and constructs INDRA Statements from these extractions. With the exception of the ISI/AMR system, each system performs its own named entity recognition and normalization; consequently, processors assign identifiers to Agents based on reader outputs. In the case of the ISI/AMR system, INDRA's processor integrates Gilda (Gyori *et al*, 2022) to assign identifiers for Agents processed from the output of the system.

Reach version 1.3.3 was downloaded from https://github.com/clulab/reach and used to process all text content for the collected corpus described in the previous section. Reach reading output was processed into INDRA Statements using the indra.sources.reach module.

 © 2023 The Authors

Sparser was obtained as an executable image from its developers and was used to process all text content for the collected corpus described in the previous section. The Sparser source code is available at https://github.com/ddmcdonald/sparser and the Sparser executable is available as part of the INDRA Docker image which can be obtained from https://hub.docker.com/r/labsyspharm/indra. Sparser reading output was processed into INDRA Statements using the indra.sources.sparser module.

MedScan reader output for the collected corpus described in the previous section was obtained from Elsevier and processed into INDRA Statements using the indra.sources.medscan module.

TRIPS/DRUM was obtained from https://github.com/wdebeaum/drum and used to process part of the text content for the collected corpus, as follows. First, we selected all papers for which only an abstract was available, then selected those papers from which Reach, Sparser, and MedScan extracted at least one Statement about any of 227 genes relevant to a key cancer signaling pathway, the Ras pathway. This resulted in a total of 42,158 abstracts which were processed with TRIPS/DRUM. The outputs were then processed into INDRA Statements using the indra.sources.trips module.

RLIMS-P reader output for PubMed abstracts and PubMedCentral full-text articles was obtained from https://hershey.dbi.udel.edu/textmining/export/ (accessed June 2019), and then filtered to the corpus of interest described in the previous section. The outputs were then processed into INDRA Statements using the indra.sources.rlimsp module.

ISI/AMR (Docker image available at https://hub.docker.com/r/sahilgar/bigmechisi) reader output was provided by the system's creators for 10,433 articles which were filtered to the corpus of interest resulting in a total of 1,878 reader outputs. These were then processed into INDRA Statements using the indra.sources.isi module.

Structured sources

In addition to text mining, we processed multiple pathway databases with INDRA to obtain INDRA Statements.

TRRUST release 4/16/2018 with human transcription factor-target relationships was obtained from https://www.grnpedia.org/trrust/data/trrust_rawdata.human.tsv and processed into INDRA Statements using the indra.sources.trrust module.

Signor content was processed through the Signor web service (https://signor.uniroma2.it/download_entity.php) in June 2019 and processed into INDRA Statements using the indra.sources.signor module.

HPRD content was obtained from http://www.hprd.org/RELEASE9/HPRD_FLAT_FILES_041310.tar.gz and processed into INDRA Statements using the indra.sources.hprd module.

BEL content was obtained from the Selventa Large Corpus available at https://raw.githubusercontent.com/cthoyt/selventa-knowledge/master/selventa_knowledge/large_corpus.bel and processed using PyBEL and the indra.sources.bel module into INDRA Statements.

CausalBioNet content was processed from JGF files from http://causalbionet.com/Content/jgf_bulk_files/Human-2.0.zip and processed into INDRA Statements using PyBEL and the indra.sources.bel module.

BioGRID content was obtained from https://downloads.thebiogrid.org/Download/BioGRID/Release-Archive/BIOGRID-4.2.192/BIOGRID-ALL-4.2.192.tab3.zip and processed into INDRA Statements using the indra.sources.biogrid module.

PhosphoSitePlus content was downloaded from https://www.phosphosite.org/staticDownloads in June 2019 via the "BioPAX: Kinase-substrate information" link, in BioPAX format, and processed into INDRA Statements using the indra.sourecs.biopax module.

Pathway Commons content was obtained from https://www.pathwaycommons.org/archives/PC2/v12/PathwayCommons12.Detailed.BIOPAX.owl.gz and processed using PyBioPAX and the indra.sources.biopax module into INDRA Statements. To account for the fact that BioGRID, PhosphoSitePlus, and HPRD content were obtained separately (and these are also available as part of Pathway Commons), we filtered out interactions from these sources when processing Pathway Commons.

The scripts to process each source as described above are available at: https://github.com/sorgerlab/indra_assembly_paper/blob/master/run_assembly/process_sources.py.

INDRA statement representation

INDRA aggregates content from reading systems and structured databases as INDRA Statements, a knowledge representation meant to capture biochemical interactions and regulation. Statements are implemented as Python classes and can be serialized into a JSON format (see schema at: https://raw.githubusercontent.com/sorgerlab/indra/master/indra/resources/statements_schema.json). Each Statement has a type (e.g., Complex, Phosphorylation, Inhibition, or IncreaseAmount; see Table EV1 for a summary of Statement types obtained from reading systems for the Benchmark Corpus) and takes one or more Agents as arguments. Agents can represent entities such as proteins, small molecules, or higher-level processes. Agents can represent a variety of molecular states important for capturing protein function: modification state, mutational state, cellular location, activity, and bound conditions. Some Statement types have additional arguments such as modification site or residue. Each Statement carries a list of Evidence objects. Evidence objects carry metadata on supporting evidence for the Statement which can include a mention extracted by a reading system from a given sentence in a publication, or an entry in a structured database. In both cases, Evidence maintains a reference to the source publication from which it was extracted. Evidence objects also contain information on context (disease, cell line, etc.) when available from the source. The Statement object model is introduced in detail in (Gyori *et al*, 2017) and is documented at: https://indra.readthedocs.io/en/latest/modules/statements.html#indra-statements-indra-statements. Documentation specific to Agent objects is at: https://indra.readthedocs.io/en/latest/modules/statements.html#indra.statements.statements.Agent and Evidence objects at: https://indra.readthedocs.io/en/latest/modules/statements.html#indra.statements.statements.Evidence.

Normalization and filtering of INDRA statements

INDRA contains a number of modules that implement normalization and filtering operations on Statements. These can be put together into custom assembly pipelines appropriate for a given application. INDRA exposes the assemble_corpus module which provides a functional interface to these functions, documented at: https://indra.readthedocs.io/en/latest/modules/tools/index.html#module-indra.tools.assemble_corpus. Here, we describe the normalization and filtering steps used in the pipeline to assemble the Benchmark Corpus.

Filtering out hypotheses

Descriptions of mechanisms in the literature are often made hypothetically, that is, rather than asserting that, for example, "A phosphorylates B," a sentence might describe "we tested whether A could phosphorylate B." Reading systems can recognize such differences in modality and set flags in their output to differentiate such Statements. These flags are then propagated by INDRA as part of the Statement representation and can be used to filter Statements. First, we removed Statements that were supported by mentions indicative of a hypothesis rather than an assertion (for instance, including sentences phrased as "we tested whether…"). Documentation: https://indra.readthedocs.io/en/latest/modules/tools/index.html#indra.tools.assemble_corpus.filter_no_hypothesis.

Mapping grounding/disambiguation

INDRA implements a GroundingMapper class that performs a number of operations to improve and normalize the identifiers associated with Agents appearing in Statements. When an Agent is extracted by a reading system, the span of text corresponding to the entity represented by the Agent is also captured. This allows INDRA to override identifiers assigned to the Agent originally by a reading system.

First, the GroundingMapper checks if there is a disambiguation model made available by Adeft (Steppi *et al*, 2020) for the text associated with the Agent. As of version 0.11.1, Adeft contains machine-learned disambiguation models for 179 acronyms including "ER," "IR," "CF," etc. These models take text surrounding the given entity text (full article text when available, otherwise an abstract) as input and return scores associated with different possible resolutions of the acronym. When Adeft is invoked and produces a prediction, INDRA takes the top-scoring identifier and sets it, overriding prior identifiers assigned to the Agent. Next, the GroundingMapper checks if Gilda (Gyori *et al*, 2022) contains a machine-learned disambiguation model for the Agent text. Gilda uses an approach similar to Adeft to train disambiguation models, however, it provides models not only for acronyms but also other ambiguous synonyms such as "PDK1" and "p44." When a Gilda model is available, it is invoked with surrounding article text as input, and the top identifier returned by Gilda is set on the Agent. If neither Adeft, nor Gilda contains an adequate disambiguation model, the GroundingMapper checks if there is an entry in its curated grounding map for the given Agent text. INDRA's grounding map is imported from FamPlex (Bachman *et al*, 2018) and was curated based on a systematic analysis of the most frequently incorrectly grounding or ungrounded entity texts by reading systems. The grounding map has around 3,500 entries and includes (but is not limited to) the most frequent synonyms for protein families and complexes, for example "Erk" and "NF-kappaB". The grounding map also contains entries for other entities such as mapping "cPARP" to the PARP1 gene. An evaluation of the improvement attributable to these mappings when overriding groundings from reading systems was done in Bachman *et al* (2018).

Once all grounding overrides have been done, the GroundingMapper performs *identifier normalization* on Agents using the INDRA ontology graph. The INDRA ontology graph combines entries across multiple ontologies and represents each entry as a graph node with a set of properties (namespace, identifier, standard name). There are three types of edges in the graph: xref (cross-reference meaning that the source node and the target node, often from different namespaces, are equivalent), isa (the source node is one of a set of entities represented by the parent node), and partof (the source node is part of a complex represented by the parent node). Each INDRA Agent has zero or more namespace/identifier pairs associated with it which constitute its grounding.

When standardizing the grounding of INDRA Agents, the xref edges of the ontology graph are traversed following all directed paths starting from each available grounding for the Agent. The namespaces and identifiers of nodes visited along these paths are then added as grounding for the Agent. Finally, a standardized name is chosen for the Agent based on its canonical identifier. Documentation: https://indra.readthedocs.io/en/latest/modules/preassembler/grounding_mapper.html.

Filtering to grounded statements

Each Statement has one or more Agent arguments, and an Agent is ungrounded if it does not have any identifiers associated with it beyond a text name. This filter removes and Statements that have any ungrounded Agents as arguments. Documentation: https://indra.readthedocs.io/en/latest/modules/tools/index.html#indra.tools.assemble_corpus.filter_grounded_only.

Filtering to genes

This filter checks each Statement's Agent arguments and determines whether each Agent represents a gene or protein (based on whether it contains an HGNC, UniProt, or FamPlex identifier). Statements that contain any Agents that are not genes are removed.

Filtering to human

This filter checks each Statement's Agent arguments and determines whether each Agent that represents a gene is a human or non-human gene. Statements that contain any Agents representing non-human genes are removed.

Normalizing sequence positions and filtering out non-canonical positions

Statements can refer to sequence residue positions on proteins in two ways: (i) via Agent arguments that have modification site conditions or (ii) directly, if the Statement is of a type that references a residue and position such as Phosphorylation Statements. Assertions in literature as well as curated pathway databases often refer to protein site positions using non-canonical position numbering, that is, numbering that does not match the reference sequence of the given protein. To normalize across these variants, INDRA uses the ProtMapper (preprint: Bachman *et al*, 2022) which draws on UniProt (The UniProt Consortium, 2019) for reference sequence data for proteins and PhosphoSitePlus (Hornbeck *et al*, 2012) for data on known phosphorylation site positions. The ProtMapper incorporates a manually curated site position map and applies a number of automated mapping rules that account for frequent patterns of position variations. Based on the results of mapping, the Agent modification condition residue positions or the Statements residue position argument is overwritten. ProtMapper also reports if it detects a position that does not match the reference sequence but cannot map it to a canonical position. INDRA uses this information to filter out Statements that refer to any such sequence positions which are usually a sign of reading errors.

 © 2023 The Authors

Procedure for identifying duplicates and refinements

When determining whether two Statements are duplicates, we require that (i) the two Statements' types are the same, (ii) all the Agent arguments of the two Statements are matching in their canonical grounding; this is determined using a built-in (but configurable) priority order of namespaces to choose a single canonical grounding for an Agent, or, if an Agent has no groundings available, its name is used as canonical grounding, (iii) all states (activity, modifications, bound conditions, location, mutations) of the matching Agents of the two Statements are equivalent, and (iv) all additional Statement arguments are equivalent (e.g., residue and position for a Modification Statement). To avoid making pairwise comparisons, we construct an equivalence key from properties (i–iv) needed to determine equivalence for each individual Statement, and then use a hash map data structure to group Statements efficiently by equivalence key. Groups of Statements having the same equivalence key are collapsed into a single Statement and their Evidences are concatenated.

For finding refinements among Statements, we make use of the INDRA ontology graph's *is a* and *part of* edges. For determining a refinement, we require that the two Statements have the same type, and that one Statement is a refinement of the other with respect to at least one of the properties (ii–iv) described above, and that the other Statement does not refine the first one based on any of these properties. In other words, if one Statement is more specific than the other according to one property but less specific according to another property, there is no refinement relationship between them at the Statement level.

Statement curation

In this paper, we created a dataset consisting of 1,689 curated Statements with 5,386 mentions for analysis of reader errors and to train and evaluate belief models. The full Curated Corpus dataset (which we call the *Extended Multi-reader Curation Dataset* below) was built up through a process of reader-specific stratified curation to create the *Reader-specific Curation Dataset* and the *Multi-reader Curation Dataset* which were used for some of the intermediate results, as described below.

Reader-specific curation dataset

Random samples of Statements were drawn with replacements from the Benchmark Corpus for curation, stratified by reader and number of mentions. For each reader, a sample of Statements was taken with different numbers of mentions from that reader, starting with one mention and continuing up to 10 mentions. Statements were curated by authors JAB and BMG by evaluating mentions from the given reader to determine whether the Statement, as extracted, was fully supported by the text from the mention. In many cases (e.g., ambiguous grounding) this required inspection of the context of the source document. From the perspective of a single reader (i.e., when fitting reader-specific models), a Statement was considered to be completely curated if all the mentions for that given reader were curated. Given correctness curations for each mention supporting a Statement, the Statement was determined to be overall correct if it was supported by at least one correctly extracted mention. The number of fully curated Statements by reader and mention count are listed in Table 2, rows marked "Complete" (note that counts in the table reflect sampled instances of Statements, in some cases the same Statement having been sampled multiple times). When fitting models at the mention level (Figs 4D and EV3, Table 3), only these complete curations are used. When showing the empirical trend of correctness at different mention counts for Reach, Sparser, and MedScan (Fig 4B), we include "Incomplete" curations (Table 2 "Incomplete") where if at least one *correctly* extracted mention was identified (even if not all mentions were explicitly curated), it was sufficient to establish the overall correctness of the Statement. Similarly, for Statements where all mentions in a manually reviewed subset were *incorrect*, the assumption was made that this subset implied the overall incorrectness of the Statement. This was justified by the fact that overall incorrectness could generally be inferred from patterns of systematic errors (e.g., misgrounding) evident in the reviewed mentions.

Multi-reader curation dataset

For assessing multi-reader overlap and training multi-reader belief models (Figs 5 and EV4), we used the union of curations for all readers from the *Reader-specific Curation Dataset*, allowing for incomplete mention curations as described above.

Curated corpus (extended multi-reader curation dataset)

In the course of the analysis of the Benchmark Corpus, additional Statements were curated to increase coverage of PPIs, particularly *Complex* and *Phosphorylation* Statements. These Statements were drawn from the Benchmark Corpus but were not stratified by the reader or mention count. This set was used for multi-reader model training and analysis for Table EV2 as well as the applications presented in Figs 6 and 7. The resulting dataset included 1,800 curated Statements with a total of 5,709 unique curated mentions (6,022 total curated mentions with some mentions curated more than once by different curators). The list of curations is available as a resource file at https://github.com/sorgerlab/indra_assembly_paper/blob/master/data/curation/indra_assembly_curations.json and on Zenodo.

Binomial, beta-binomial, and INDRA Belief models of Statement reliability

The INDRA belief model

The "INDRA belief model" represents the probability of a Statement being correct as the result of a two-step random process (Fig 4C). The first process considers the probability that a Statement is drawn from the pool of Statements that are *always* incorrect, regardless of the number of mentions they have. This probability is based on the *systematic error* parameter for each reading system. If the Statement is *not* from this pool, then its reliability is alternatively modeled to follow a binomial distribution assuming a particular *random error* rate for that source. Like the beta-binomial model, the INDRA belief model captures the plateau in Statement reliability (Fig 4D), though the predicted distributions for mention correctness do not correspond well to the empirical U-shaped distribution (Fig EV3A).

The INDRA Belief Model is calculated based on the Evidence objects belonging to a Statement, each Evidence corresponding to a mention produced by a source such as a text mining system or a pathway database integrated with INDRA. In the simple case of a single knowledge source, we define the belief of a Statement with n Evidences as

$$1-\left(e_{syst}+e_{rand}^{n}\left(1-e_{syst}\right)\right)$$

where e_{syst} and e_{rand} are the systematic and random error parameters for the given source, respectively.

© 2023 The Authors

This model can also be generalized to multiple sources as follows. Assume there are a total of K known sources $S = \{S_1, S_2, \ldots, S_K\}$, each associated with a random and systematic error rate. For source S_k, $_e$ will denote the systematic error rate, and $_e$ the random error rate. Given a set of N Evidences for Statement T $E_T = \{E_{T,1}, \ldots, E_{T,N}\}$, with Source$(_E) \in S$ corresponding to the source of evidence of $E_{T,i}$, we introduce $N_{T,k}$, the number of Evidences for Statement T from source S_k:

$$N_{T,k} = \sum_{i=1}^{N} I\left(\text{Source}\left(E_{T,i}\right), S_k\right)$$

where $I(X, Y)$ stands for the indicator function which evaluates to 1 if $X = Y$, and 0 otherwise. We then define the belief of Statement T as follows:

$$B(T) = 1 - \prod_{k=1}^{K} \left(e_{k,syst} \cdot \min\left(1, N_{T,k}\right) + e_{k,rand}^{N_{T,k}} \left(1 - e_{k,syst} \cdot \min\left(1, \mathrm{N}_{T,K}\right)\right)\right).$$

For the calculation of beliefs for a Statement that is refined by other Statements, we introduce the extended Evidence set denoted as $E'(T)$ which is defined as

$$E'_T = \bigcup\left(E_T, \bigcup_{X \in P_T} E'_X\right).$$

Here, $X \in P_T$ if and only if X refines T. In other words, we take the union of all Evidences for the Statement itself and all the Statements by which it is refined, recursively. We then apply the equation for $N_{T,k}$ and $B(T)$ to $E'(T)$ instead of $E(T)$ in the obvious way.

When the quality of fit of the three different models was compared using maximum likelihood parameter values, the original belief model performed very slightly better than the beta-binomial model for both the Reach and Sparser reading systems (Table 3).

The Binomial and Beta-binomial belief models

The binomial model treats every individual mention as a Bernoulli trial, where the probability of a single reading system being jointly incorrect for all sentences decreases according to a binomial distribution (e.g., the probability of incorrectly processing 10 sentences is analogous to flipping a coin 10 times and getting 10 tails). The binomial model substantially overestimates the reliability of Statements with three or more Evidences from Reach, due to the fact that it does not account for systematic errors (Fig 4D). In addition, the binomial model predicts that for a Statement with n Evidences, the mode of the distribution of the number of correct Evidences is close to $n/2$ (bell-shaped red curves in Fig EV3B), whereas the curation data shows that Evidences are more likely to be either all incorrect (zero bars) or all correct (right-most bars).

The binomial belief model has a single random error rate parameter e_{rand} for each source, and—making use of definitions from the previous section—the belief for a Statement T with Evidences from K sources can be calculated as

$$B(T) = 1 - \prod_{k=1}^{K} e_{k,rand}^{N_{T,k}}.$$

The beta-binomial model is based on a binomial model where the probability of each mention being correctly extracted is itself drawn from a beta distribution (Wilcox, 1979). The beta-binomial model better captures the tendency of Statement reliability to plateau below 100% (Fig 4D) as well as the U-shaped distributions of the numbers of underlying correct evidence (Fig EV3C).

The beta-binomial belief model has two parameters for each source, α and β, and for a Statement with Evidences from K sources, it can be calculated as

$$B(T) = 1 - \prod_{k=1}^{K} \frac{Beta\left(\alpha_k, N_{T,k} + \beta_k\right)}{Beta\left(\alpha_k, \beta_k\right)}.$$

where *Beta* is the standard beta-function.

Parameter estimation for INDRA belief, binomial, and Beta-Binomial models

Parameter estimation for the belief models was performed by affine-invariant MCMC as implemented by the *emcee* software package (Foreman-Mackey *et al*, 2013). The likelihood function for each model was derived from the functions $B(T)$ as described above. MCMC was performed with 100 walkers running for 100 burn-in steps followed by 100 sampling steps. Python code implementing the MCMC runs is in the GitHub repository for the paper in modules bioexp/curation/process_curations.py and bioexp/curation/model_fits.py.

Machine-learned models of statement reliability

Model types and evaluation

Classification models evaluated for their ability to predict Statement correctness were obtained from the Python package *sklearn*. Evaluated models included Support Vector Classification (*sklearn.svm.SVC* with probability estimation enabled), k-Nearest Neighbors (*sklearn.neighbors.KNeighborsClassifier*, used with default parameters), logistic regression with log-transformed mention counts (*sklearn.linear_model.LogisticRegression*), and Random Forests (*sklearn.ensemble.RandomForestClassifier* with *n_estimators = 2000* and *max_depth = 13*, obtained by manual hyper-parameter optimization). Model performance was evaluated by 10-fold cross-validation; each fold was used to calculate the AUPRC for the held-out data. Values in Table EV2 reflect the mean AUPRC values across the 10 folds.

Encoding of features for Statement belief prediction

Reader mention counts. Mention counts for each reader were included as distinct features (columns) for each Statement. When incorporating evidence from more specific Statements ("specific evidences" in Table EV2) these were added in a separate set of columns for each reader; a Statement could thus have two columns with Reach mention counts, one for mentions directly supporting the Statement, and another for mentions obtained from more specific Statements.

Number of unique PMIDs. Unique PMIDs supporting each Statement were obtained from its mentions and added as a single feature. When incorporating evidence from more specific Statements, an

© 2023 The Authors

additional feature was added for the number of unique PMIDs supporting these Statements.

Statement type. Statement types were one-hot encoded (one binary feature for each type, Activation, Inhibition, Phosphorylation, etc.)

Average evidence length. Mention texts directly supporting the Statement were split by whitespace; the number of resulting substrings were counted and averaged across all mentions and included as a feature.

"Promoter" frequency. The number of mention texts containing the term "promoter" were counted and the resulting value was divided by the total number of mentions to obtain a frequency of the occurrence of this keyword.

Data availability

INDRA is available at https://github.com/sorgerlab/indra under an open-source BSD 2-clause license. The source code used to generate results in this paper is available at https://github.com/sorgerlab/indra_assembly_paper. The INDRA Benchmark Corpus and the Curation Corpus used to train belief models are available on Zenodo at https://doi.org/10.5281/zenodo.7559353.

Expanded View for this article is available online.

Acknowledgements
This work was supported by DARPA grants W911NF-15-1-0544 and W911NF-20-1-0255 and by NCI grant U54-CA225088. We are grateful to the developers of Reach, Sparser, MedScan, RLIMS-P, TRIPS, and ISI/AMR for their support in integrating their reading systems; to Lynette Hirschman, Matthew Peterson, Tonia Korves, Christopher Garay, and Robyn Kozierok for their work on system evaluation and useful suggestions; to members of the Laboratory of Systems Pharmacology Klas Karis, Albert Steppi, Charles Hoyt, Patrick Greene, Diana Kolusheva, and Daniel Milstein for their contributions to INDRA; and to Juliann Tefft for her helpful comments on the manuscript.

Author contributions
John A Bachman: Conceptualization; data curation; software; methodology; writing – original draft; writing – review and editing. **Benjamin M Gyori:** Conceptualization; data curation; software; funding acquisition; methodology; writing – original draft; writing – review and editing. **Peter K Sorger:** Conceptualization; funding acquisition; writing – original draft; writing – review and editing.

Disclosure and competing interests statement
PKS is a co-founder and member of the BOD of Glencoe Software, a member of the BOD for Applied Biomath, and a member of the SAB for RareCyte, NanoString, and Montai Health; he holds equity in Glencoe, Applied Biomath, and RareCyte. PKS is a consultant for Merck and the Sorger lab has received research funding from Novartis and Merck in the past 5 years. PKS declares that none of these activities have influenced the content of this manuscript. JAB is currently an employee of Google, LLC. BMG declares no outside interests. PKS is an editorial advisory board member. This has no bearing on the editorial consideration of this article for publication.

References

Ahmed Y, Telmer CA, Miskov-Zivanov N (2021) CLARINET: efficient learning of dynamic network models from literature. *Bioinform Adv* 1: vbab006

Allen J, de Beaumont W, Galescu L, Teng CM (2015) Complex event extraction using DRUM. *ACL-IJCNLP* 2015: 1–11

Alstott J, Bullmore E, Plenz D (2014) Powerlaw: a python package for analysis of heavy-tailed distributions. *PLoS ONE* 9: e85777

Ananiadou S, Thompson P, Nawaz R, McNaught J, Kell DB (2015) Event-based text mining for biology and functional genomics. *Brief Funct Genomics* 14: 213–230

Ashburner M, Ball CA, Blake JA, Botstein D, Butler H, Cherry JM, Davis AP, Dolinski K, Dwight SS, Eppig JT *et al* (2000) Gene ontology: tool for the unification of biology. The Gene Ontology Consortium. *Nat Genet* 25: 25–29

Babur O, Dogrusoz U, Demir E, Sander C (2009) ChiBE: interactive visualization and manipulation of BioPAX pathway models. *Bioinformatics* 26: 429–431

Babur Ö, Gönen M, Aksoy BA, Schultz N, Ciriello G, Sander C, Demir E (2015) Systematic identification of cancer driving signaling pathways based on mutual exclusivity of genomic alterations. *Genome Biol* 16: 45

Babur Ö, Luna A, Korkut A, Durupinar F, Siper MC, Dogrusoz U, Vaca Jacome AS, Peckner R, Christianson KE, Jaffe JD *et al* (2021) Causal interactions from proteomic profiles: molecular data meet pathway knowledge. *Patterns (N Y)* 2: 100257

Bachman JA, Gyori BM, Sorger PK (2018) FamPlex: a resource for entity recognition and relationship resolution of human protein families and complexes in biomedical text mining. *BMC Bioinformatics* 19: 248

Bachman JA, Sorger PK, Gyori BM (2022) Assembling a corpus of phosphoproteomic annotations using ProtMapper to normalize site information from databases and text mining. *bioRxiv* https://doi.org/10.1101/822668 [PREPRINT]

Baker M (2016) 1,500 scientists lift the lid on reproducibility. *Nature* 533: 452–454

Benseddik K, Sen Nkwe N, Daou P, Verdier-Pinard P, Badache A (2013) ErbB2-dependent chemotaxis requires microtubule capture and stabilization coordinated by distinct signaling pathways. *PLoS ONE* 8: e55211

Bourne PE, Lorsch JR, Green ED (2015) Perspective: sustaining the big-data ecosystem. *Nature* 527: S16–S17

Cerami EG, Gross BE, Demir E, Rodchenkov I, Babur O, Anwar N, Schultz N, Bader GD, Sander C (2011) Pathway commons, a web resource for biological pathway data. *Nucleic Acids Res* 39: D685–D690

Clauset A, Shalizi CR, Newman MEJ (2009) Power-law distributions in empirical data. *SIAM Rev* 51: 661–703

Coppari E, Yamada T, Bizzarri AR, Beattie CW, Cannistraro S (2014) A nanotechnological, molecular-modeling, and immunological approach to study the interaction of the anti-tumorigenic peptide p28 with the p53 family of proteins. *Int J Nanomedicine* 9: 1799–1813

Craver CF, Darden L (2013) *In search of mechanisms: discoveries across the life sciences*. Chicago, IL: University of Chicago Press

Danchev V, Rzhetsky A, Evans JA (2019) Centralized scientific communities are less likely to generate replicable results. *Elife* 8: e43094

Demir E, Cary MP, Paley S, Fukuda K, Lemer C, Vastrik I, Wu G, D'Eustachio P, Schaefer C, Luciano J *et al* (2010) BioPAX – a community standard for pathway data sharing. *Nat Biotechnol* 28: 935–942

Dempster JM, Pacini C, Pantel S, Behan FM, Green T, Krill-Burger J, Beaver CM, Younger ST, Zhivich V, Najgebauer H *et al* (2019) Agreement between two large pan-cancer CRISPR-Cas9 gene dependency data sets. *Nat Commun* 10: 5817

Deng Y, Bhattacharya S, Swamy OR, Tandon R, Wang Y, Janda R, Riedel H (2003) Growth factor receptor-binding protein 10 (Grb10) as a partner of phosphatidylinositol 3-kinase in metabolic insulin action. *J Biol Chem* 278: 39311–39322

© 2023 The Authors

Doherty LM, Mills CE, Boswell SA, Liu X, Hoyt CT, Gyori BM, Buhrlage SJ, Sorger PK (2021) Integrating multi-omics data reveals function and therapeutic potential of deubiquitinating enzymes. *Elife* 11: e72879

Dugourd A, Kuppe C, Sciacovelli M, Gjerga E, Gabor A, Emdal KB, Vieira V, Bekker-Jensen DB, Kranz J, Bindels EMJ *et al* (2021) Causal integration of multi-omics data with prior knowledge to generate mechanistic hypotheses. *Mol Syst Biol* 17: e9730

Fabregat A, Jupe S, Matthews L, Sidiropoulos K, Gillespie M, Garapati P, Haw R, Jassal B, Korninger F, May B *et al* (2018) The Reactome pathway knowledgebase. *Nucleic Acids Res* 46: D649–D655

Foreman-Mackey D, Hogg DW, Lang D, Goodman J (2013) Emcee: the MCMC hammer. *Publ Astron Soc Pac* 125: 306–312

Forscher BK (1963) Chaos in the brickyard. *Science* 142: 339

Frank SR, Köllmann CP, van Lidth de Jeude JF, Thiagarajah JR, Engelholm LH, Frödin M, Hansen SH (2017) The focal adhesion-associated proteins DOCK5 and GIT2 comprise a rheostat in control of epithelial invasion. *Oncogene* 36: 1816–1828

Garg S, Galstyan A, Hermjakob U, Marcu D (2016) Extracting biomolecular interactions using semantic parsing of biomedical text. In *Thirtieth AAAI Conference On Artificial Intelligence*, pp 2718–2726. Phoenix, AZ: AAAI Press

Glavaški M, Velicki L (2021) Humans and machines in biomedical knowledge curation: hypertrophic cardiomyopathy molecular mechanisms' representation. *BioData Min* 14: 45

Gyori BM, Bachman JA (2021) From knowledge to models: automated modeling in systems and synthetic biology. *Curr Opin Syst Biol* 28: 100362

Gyori BM, Bachman JA, Subramanian K, Muhlich JL, Galescu L, Sorger PK (2017) From word models to executable models of signaling networks using automated assembly. *Mol Syst Biol* 13: 954

Gyori BM, Bachman JA, Kolusheva D (2021) A self-updating causal model of COVID-19 mechanisms built from the scientific literature. In *BioCreative VII Challenge Evaluation Workshop* p 249

Gyori BM, Hoyt CT, Steppi A (2022) Gilda: biomedical entity text normalization with machine-learned disambiguation as a service. *Bioinform Adv* 2: vbac034

Hastings J, Owen G, Dekker A, Ennis M, Kale N, Muthukrishnan V, Turner S, Swainston N, Mendes P, Steinbeck C (2016) ChEBI in 2016: improved services and an expanding collection of metabolites. *Nucleic Acids Res* 44: D1214–D1219

Himmelstein DS, Lizee A, Hessler C, Brueggeman L, Chen SL, Hadley D, Green A, Khankhanian P, Baranzini SE (2017) Systematic integration of biomedical knowledge prioritizes drugs for repurposing. *Elife* 6: e26726

Holtzapple E, Telmer CA, Miskov-Zivanov N (2020) FLUTE: fast and reliable knowledge retrieval from biomedical literature. *Database* 2020: baaa056

Hornbeck PV, Kornhauser JM, Tkachev S, Zhang B, Skrzypek E, Murray B, Latham V, Sullivan M (2012) PhosphoSitePlus: a comprehensive resource for investigating the structure and function of experimentally determined post-translational modifications in man and mouse. *Nucleic Acids Res* 40: D261–D270

Hoyt CT, Domingo-Fernández D, Aldisi R, Xu L, Kolpeja K, Spalek S, Wollert E, Bachman J, Gyori BM, Greene P *et al* (2019a) Re-curation and rational enrichment of knowledge graphs in biological expression language. *Database* 2019: baz068

Hoyt CT, Domingo-Fernández D, Mubeen S, Llaó JM, Konotopez A, Ebeling C, Birkenbihl C, Muslu Ö, English B, Müller S *et al* (2019b) Integration of structured biological data sources using biological expression language. *bioRxiv* https://doi.org/10.1101/631812 [PREPRINT]

Hucka M, Finney A, Sauro HM, Bolouri H, Doyle JC, Kitano H, Arkin AP, Bornstein BJ, Bray D, Cornish-Bowden A *et al* (2003) The systems biology markup language (SBML): a medium for representation and exchange of biochemical network models. *Bioinformatics* 19: 524–531

Ietswaart R, Gyori BM, Bachman JA, Sorger PK, Churchman LS (2021) GeneWalk identifies relevant gene functions for a biological context using network representation learning. *Genome Biol* 22: 55

Islamaj Dogan R, Kim S, Chatr-aryamontri A, Wei C-H, Comeau DC, Antunes R, Matos S, Chen Q, Elangovan A, Panyam NC *et al* (2019) Overview of the BioCreative VI precision medicine track: mining protein interactions and mutations for precision medicine. *Database* 2019: bay147

Jensen LJ, Kuhn M, Stark M, Chaffron S, Creevey C, Muller J, Doerks T, Julien P, Roth A, Simonovic M *et al* (2009) STRING 8–a global view on proteins and their functional interactions in 630 organisms. *Nucleic Acids Res* 37: D412–D416

Jia S, Xiang Y, Chen X (2019) Triple trustworthiness measurement for knowledge graph. *World Wide Web Conf* 19: 2865–2871

Keegan AD, Zamorano J, Keselman A, Heller NM (2018) IL-4 and IL-13 receptor signaling from 4PS to insulin receptor substrate 2: there and Back again, a historical view. *Front Immunol* 9: 1037

Kemper B, Matsuzaki T, Matsuoka Y, Tsuruoka Y, Kitano H, Ananiadou S, Tsujii J (2010) PathText: a text mining integrator for biological pathway visualizations. *Bioinformatics* 26: i374–i381

Kumar SM, Dai J, Li S, Yang R, Yu H, Nathanson KL, Liu S, Zhou H, Guo J, Xu X (2014) Human skin neural crest progenitor cells are susceptible to BRAFV600E-induced transformation. *Oncogene* 33: 832–841

Lee PL, Ohlson MB, Pfeffer SR (2015) Rab6 regulation of the kinesin family KIF1C motor domain contributes to Golgi tethering. *Elife* 4: e06029

Lopez CF, Muhlich JL, Bachman JA, Sorger PK (2013) Programming biological models in python using PySB. *Mol Syst Biol* 9: 646

Madan S, Szostak J, Komandur Elayavilli R, Tsai RT-H, Ali M, Qian L, Rastegar-Mojarad M, Hoeng J, Fluck J (2019) The extraction of complex relationships and their conversion to biological expression language (BEL) overview of the BioCreative VI (2017) BEL track. *Database* 2019: baz084

McDonald DD, Friedman SE, Paullada A, Bobrow R, Burstein MH (2016) Extending biology models with deep NLP over scientific articles. AAAI Workshop: Knowledge Extraction from Text

Meyers RM, Bryan JG, McFarland JM, Weir BA, Sizemore AE, Xu H, Dharia NV, Montgomery PG, Cowley GS, Pantel S *et al* (2017) Computational correction of copy number effect improves specificity of CRISPR–Cas9 essentiality screens in cancer cells. *Nat Genet* 49: 1779–1784

Mishra GR (2006) Human protein reference database—2006 update. *Nucleic Acids Res* 34: D411–D414

Moody TW, Di Florio A, Jensen RT (2012) PYK-2 is tyrosine phosphorylated after activation of pituitary adenylate cyclase activating polypeptide receptors in lung cancer cells. *J Mol Neurosci* 48: 660–666

Moret N, Liu C, Gyori BM, Bachman JA, Steppi A, Hug C, Taujale R, Huang L-C, Berginski ME, Gomez SM *et al* (2021) A resource for exploring the understudied human kinome for research and therapeutic opportunities. *bioRxiv* https://doi.org/10.1101/2020.04.02.022277 [PREPRINT]

Mori K, Giovannone B, Smith RJ (2005) Distinct Grb10 domain requirements for effects on glucose uptake and insulin signaling. *Mol Cell Endocrinol* 230: 39–50

Neil D, Briody J, Lacoste A, Sim A, Creed P, Saffari A (2018) Interpretable graph convolutional neural networks for inference on Noisy knowledge graphs. *ArXiv* https://doi.org/10.48550/arXiv.1812.00279 [PREPRINT]

Noriega-Atala E, Liang Z, Bachman J, Morrison C, Surdeanu M (2019) Understanding the polarity of events in the biomedical literature: deep learning vs. linguistically-informed methods. In *Proceedings of the*

 © 2023 The Authors

Workshop on Extracting Structured Knowledge from Scientific Publications, pp 21–30. Minneapolis, MN: Association for Computational Linguistics

Noriega-Atala E, Hein PD, Thumsi SS, Wong Z, Wang X, Hendryx SM, Morrison CT (2020) Extracting inter-sentence relations for associating biological context with events in biomedical texts. *IEEE/ACM Trans Comput Biol Bioinform* 17: 1895–1906

Novichkova S, Egorov S, Daraselia N (2003) MedScan, a natural language processing engine for MEDLINE abstracts. *Bioinformatics* 19: 1699–1706

Nunes KP, Rigsby CS, Webb RC (2010) RhoA/rho-kinase and vascular diseases: what is the link? *Cell Mol Life Sci* 67: 3823–3836

Ostaszewski M, Niarakis A, Mazein A, Kuperstein I, Phair R, Orta-Resendiz A, Singh V, Aghamiri SS, Acencio ML, Glaab E *et al* (2021) COVID19 disease map, a computational knowledge repository of virus-host interaction mechanisms. *Mol Syst Biol* 17: e10387

Oughtred R, Stark C, Breitkreutz B-J, Rust J, Boucher L, Chang C, Kolas N, O'Donnell L, Leung G, McAdam R *et al* (2019) The BioGRID interaction database: 2019 update. *Nucleic Acids Res* 47: D529–D541

Pan J, Meyers RM, Michel BC, Mashtalir N, Sizemore AE, Wells JN, Cassel SH, Vazquez F, Weir BA, Hahn WC *et al* (2018) Interrogation of mammalian protein complex structure, function, and membership using genome-scale fitness screens. *Cell Syst* 6: 555–568

Park S-Y, Schinkmann KA, Avraham S (2006) RAFTK/Pyk2 mediates LPA-induced PC12 cell migration. *Cell Signal* 18: 1063–1071

Perfetto L, Briganti L, Calderone A, Perpetuini AC, Iannuccelli M, Langone F, Licata L, Marinkovic M, Mattioni A, Pavlidou T *et al* (2016) SIGNOR: a database of causal relationships between biological entities. *Nucleic Acids Res* 44: D548–D554

Rahman M, Billmann M, Costanzo M, Aregger M, Tong AHY, Chan K, Ward HN, Brown KR, Andrews BJ, Boone C *et al* (2021) A method for benchmarking genetic screens reveals a predominant mitochondrial bias. *Mol Syst Biol* 17: e10013

Rath S, Sharma R, Gupta R, Ast T, Chan C, Durham TJ, Goodman RP, Grabarek Z, Haas ME, Hung WHW *et al* (2021) MitoCarta3.0: an updated mitochondrial proteome now with sub-organelle localization and pathway annotations. *Nucleic Acids Res* 49: D1541–D1547

Rodchenkov I, Babur O, Luna A, Aksoy BA, Wong JV, Fong D, Franz M, Siper MC, Cheung M, Wrana M *et al* (2020) Pathway commons 2019 update: integration, analysis and exploration of pathway data. *Nucleic Acids Res* 48: D489–D497

Saez-Rodriguez J, Alexopoulos LG, Epperlein J, Samaga R, Lauffenburger DA, Klamt S, Sorger PK (2009) Discrete logic modelling as a means to link protein signalling networks with functional analysis of mammalian signal transduction. *Mol Syst Biol* 5: 331

Sayan BS, Yang AL, Conforti F, Tucci P, Piro MC, Browne GJ, Agostini M, Bernardini S, Knight RA, Mak TW *et al* (2010) Differential control of TAp73 and DeltaNp73 protein stability by the ring finger ubiquitin ligase PIR2. *Proc Natl Acad Sci USA* 107: 12877–12882

Schaefer CF, Anthony K, Krupa S, Buchoff J, Day M, Hannay T, Buetow KH (2009) PID: the pathway interaction database. *Nucleic Acids Res* 37: D674–D679

Sharp R, Pyarelal A, Gyori B, Alcock K, Laparra E, Valenzuela-Escárcega MA, Nagesh A, Yadav V, Bachman J, Tang Z *et al* (2019) Eidos, INDRA, & Delphi: from free text to executable causal models. In *Proceedings of the 2019 Conference of the North American Chapter of the Association for Computational Linguistics (Demonstrations)*, pp 42–47. Minneapolis, MN: Association for Computational Linguistics

Shimada K, Bachman JA, Muhlich JL, Mitchison TJ (2021) shinyDepMap, a tool to identify targetable cancer genes and their functional connections from cancer dependency map data. *Elife* 10: e57116

Sokolov A, Carlin DE, Paull EO, Baertsch R, Stuart JM (2016) Pathway-based genomics prediction using generalized elastic net. *PLoS Comput Biol* 12: e1004790

Steppi A, Gyori B, Bachman J (2020) Adeft: acromine-based disambiguation of entities from text with applications to the biomedical literature. *J Open Source Softw* 5: 1708

Surks HK, Richards CT, Mendelsohn ME (2003) Myosin phosphatase-rho interacting protein. A new member of the myosin phosphatase complex that directly binds RhoA. *J Biol Chem* 278: 51484–51493

Szklarczyk D, Gable AL, Nastou KC, Lyon D, Kirsch R, Pyysalo S, Doncheva NT, Legeay M, Fang T, Bork P *et al* (2021) The STRING database in 2021: customizable protein–protein networks, and functional characterization of user-uploaded gene/measurement sets. *Nucleic Acids Res* 49: D605–D612

The UniProt Consortium (2019) UniProt: a worldwide hub of protein knowledge. *Nucleic Acids Res* 47: D506–D515

Torii M, Arighi CN, Li G, Wang Q, Wu CH, Vijay-Shanker K (2015) RLIMS-P 2.0: a generalizable rule-based information extraction system for literature Mining of Protein Phosphorylation Information. *IEEE/ACM Trans Comput Biol Bioinform* 12: 17–29

Tsherniak A, Vazquez F, Montgomery PG, Weir BA, Kryukov G, Cowley GS, Gill S, Harrington WF, Pantel S, Krill-Burger JM *et al* (2017) Defining a cancer dependency map. *Cell* 170: 564–576

Tuncbag N, Gosline SJC, Kedaigle A, Soltis AR, Gitter A, Fraenkel E (2016) Network-based interpretation of diverse high-throughput datasets through the omics integrator software package. *PLoS Comput Biol* 12: e1004879

Türei D, Korcsmáros T, Saez-Rodriguez J (2016) OmniPath: guidelines and gateway for literature-curated signaling pathway resources. *Nat Methods* 13: 966–967

Valenzuela-Escárcega MA, Babur Ö, Hahn-Powell G, Bell D, Hicks T, Noriega-Atala E, Wang X, Surdeanu M, Demir E, Morrison CT (2018) Large-scale automated machine reading discovers new cancer-driving mechanisms. *Database* 2018: bay098

Van Landeghem S, Ginter F, Van de Peer Y, Salakoski T (2011) EVEX: a PubMed-scale resource for homology-based generalization of text mining predictions. In *Proceedings of BioNLP 2011 Workshop*, pp 28–37. Portland, OR: Association for Computational Linguistics

Van Landeghem S, Björne J, Wei C-H, Hakala K, Pyysalo S, Ananiadou S, Kao H-Y, Lu Z, Salakoski T, Van de Peer Y *et al* (2013) Large-scale event extraction from literature with multi-level gene normalization. *PLoS ONE* 8: e55814

Vermehren-Schmaedick A, Krueger W, Jacob T, Ramunno-Johnson D, Balkowiec A, Lidke KA, Vu TQ (2014) Heterogeneous intracellular trafficking dynamics of brain-derived neurotrophic factor complexes in the neuronal Soma revealed by single quantum dot tracking. *PLoS ONE* 9: e95113

Wang X, Ratnam J, Zou B, England PM, Basbaum AI (2009a) TrkB signaling is required for both the induction and maintenance of tissue and nerve injury-induced persistent pain. *J Neurosci* 29: 5508–5515

Wang Y, Zheng XR, Riddick N, Bryden M, Baur W, Zhang X, Surks HK (2009b) ROCK isoform regulation of myosin phosphatase and contractility in vascular smooth muscle cells. *Circ Res* 104: 531–540

Wellbrock C, Rana S, Paterson H, Pickersgill H, Brummelkamp T, Marais R (2008) Oncogenic BRAF regulates melanoma proliferation through the lineage specific factor MITF. *PLoS ONE* 3: e2734

Westergaard D, Stærfeldt H-H, Tønsberg C, Jensen LJ, Brunak S (2018) A comprehensive and quantitative comparison of text-mining in 15 million full-text articles versus their corresponding abstracts. *PLoS Comput Biol* 14: e1005962

© 2023 The Authors

Wick KR, Werner ED, Langlais P, Ramos FJ, Dong LQ, Shoelson SE, Liu F (2003) Grb10 inhibits insulin-stimulated insulin receptor substrate (IRS)-phosphatidylinositol 3-kinase/Akt signaling pathway by disrupting the association of IRS-1/IRS-2 with the insulin receptor. *J Biol Chem* 278: 8460–8467

Wilcox RR (1979) Estimating the parameters of the Beta-binomial distribution. *Educ Psychol Meas* 39: 527–535

Wong JV, Franz M, Siper MC, Fong D, Durupinar F, Dallago C, Luna A, Giorgi J, Rodchenkov I, Babur Ö *et al* (2021) Author-sourced capture of pathway knowledge in computable form using biofactoid. *Elife* 10: e68292

Wooten DJ, Gebru M, Wang H-G, Albert R (2021) Data-driven math model of FLT3-ITD acute myeloid leukemia reveals potential therapeutic targets. *J Pers Med* 11: 193

Wu H, Zeinab RA, Flores ER, Leng RP (2011) Pirh2, a ubiquitin E3 ligase, inhibits p73 transcriptional activity by promoting its ubiquitination. *Mol Cancer Res* 9: 1780–1790

Yuryev A, Mulyukov Z, Kotelnikova E, Maslov S, Egorov S, Nikitin A, Daraselia N, Mazo I (2006) Automatic pathway building in biological association networks. *BMC Bioinformatics* 7: 171

Zhang H (2004) The optimality of naive Bayes. In *Proceedings of the Seventeenth International Florida Artificial Intelligence Research Society Conference (FLAIRS 2004)*, pp 562–567. Miami Beach, FL: AAAI Press

Zucker J, Paneri K, Mohammad-Taheri S, Bhargava S, Kolambkar P, Bakker C, Teuton J, Hoyt CT, Oxford K, Ness R (2021) Leveraging structured biological knowledge for counterfactual inference: a case study of viral pathogenesis. *IEEE Trans Big Data* 7: 25–37

License: This is an open access article under the terms of the Creative Commons Attribution License, which permits use, distribution and reproduction in any medium, provided the original work is properly cited.

 © 2023 The Authors

Expanded View Figures

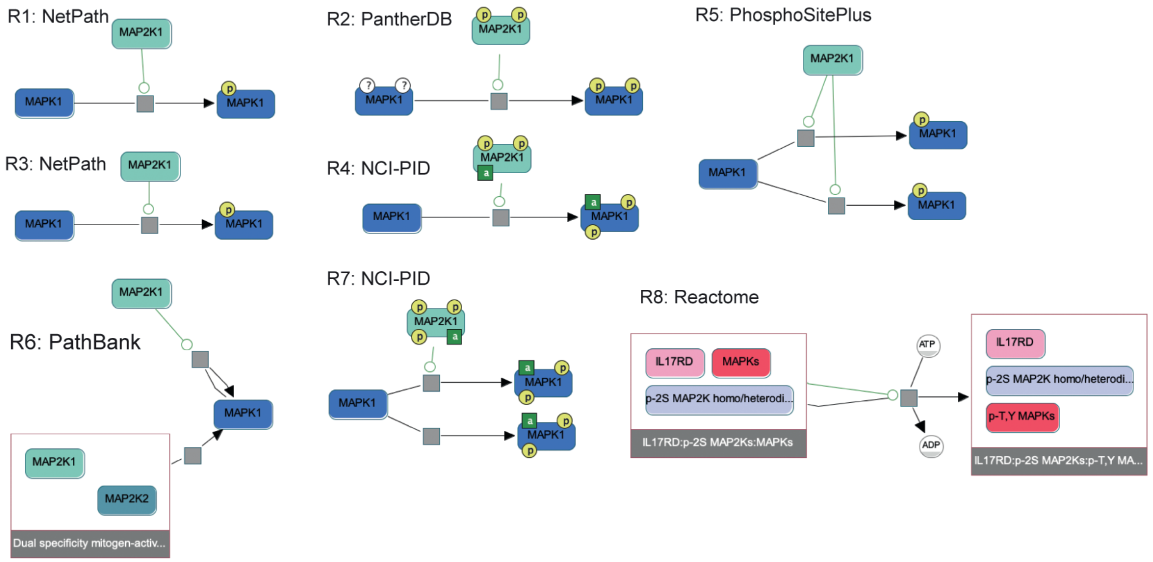

Figure EV1. Differences in curation practices across databases integrated by Pathway Commons.

A subgraph of the “paths-from-to” query between MAP2K1 and MAPK1 obtained from Pathway Commons and visualized using the ChiBE software (Babur *et al*, 2009). Each biochemical reaction (R1–R8) depicts a different curation of the same reaction in which MAP2K1 phosphorylates MAPK1. The original source database (e.g., NetPath) is shown next to each reaction. Inconsistencies include (i) the reaction structure itself, with some specifying a single step phosphorylation of two sites (e.g., R4) while others specify single-site phosphorylation (e.g., R1), or the explicit representation of ADP and ATP as part of the reaction (R8); (ii) the phosphorylation status of MAP2K1, with no phosphorylation status given in R1, R3, R5, R6, and R8, two phosphorylation sites indicated in R2, R4, and three phosphorylation sites in R7; (iii) the initial state of MAPK1, with R2 explicitly indicating unphosphorylated status, while other reactions do not make this explicit; (iv) the final state of MAPK1, with some reactions representing MAPK1 phosphorylation on an unspecified site (R1 and R3), and others providing specific phosphorylation sites (e.g., R2); (v) the specification of active states, with R4 being the only reaction representing MAP2K1 explicitly as active, while R4 and R7 are the only reactions specifying that MAPK1 is active after phosphorylation; and (vi) the presence of other co-factors such as IL17RD (R8) as part of the reaction.

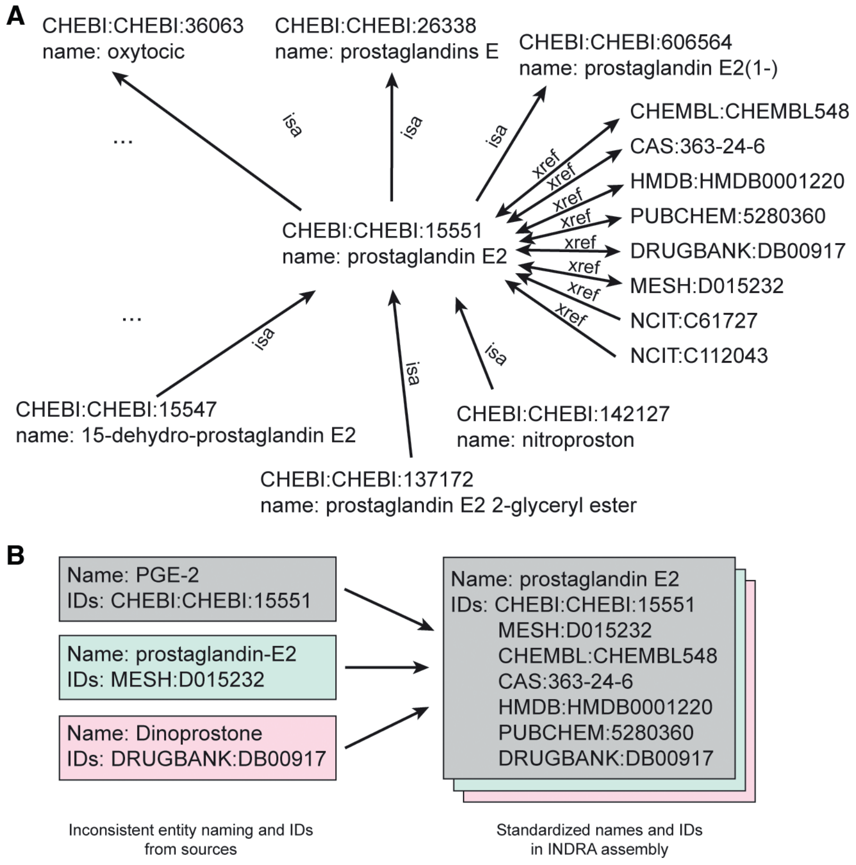

Figure EV2. Ontology graph guiding INDRA knowledge assembly.

A A subgraph of the INDRA ontology graph showing the neighborhood of the node representing "prostaglandin E2" in the ChEBI database (CHEBI: 15551). Edges represent "isa" relationships to more general terms (and from more specific terms), and "xref" edges represent identifier equivalence to nodes representing entries in other databases including MeSH, DrugBank, ChEMBL, CAS, PubChem, and NCIT. Each ontology graph node also provides a name that can be used for standardization and display purposes.

B Example of three entities with inconsistent names and identifiers which, when standardized by INDRA using the ontology graph, are normalized to consistent entities with identical names and sets of identifiers.

 © 2023 The Authors

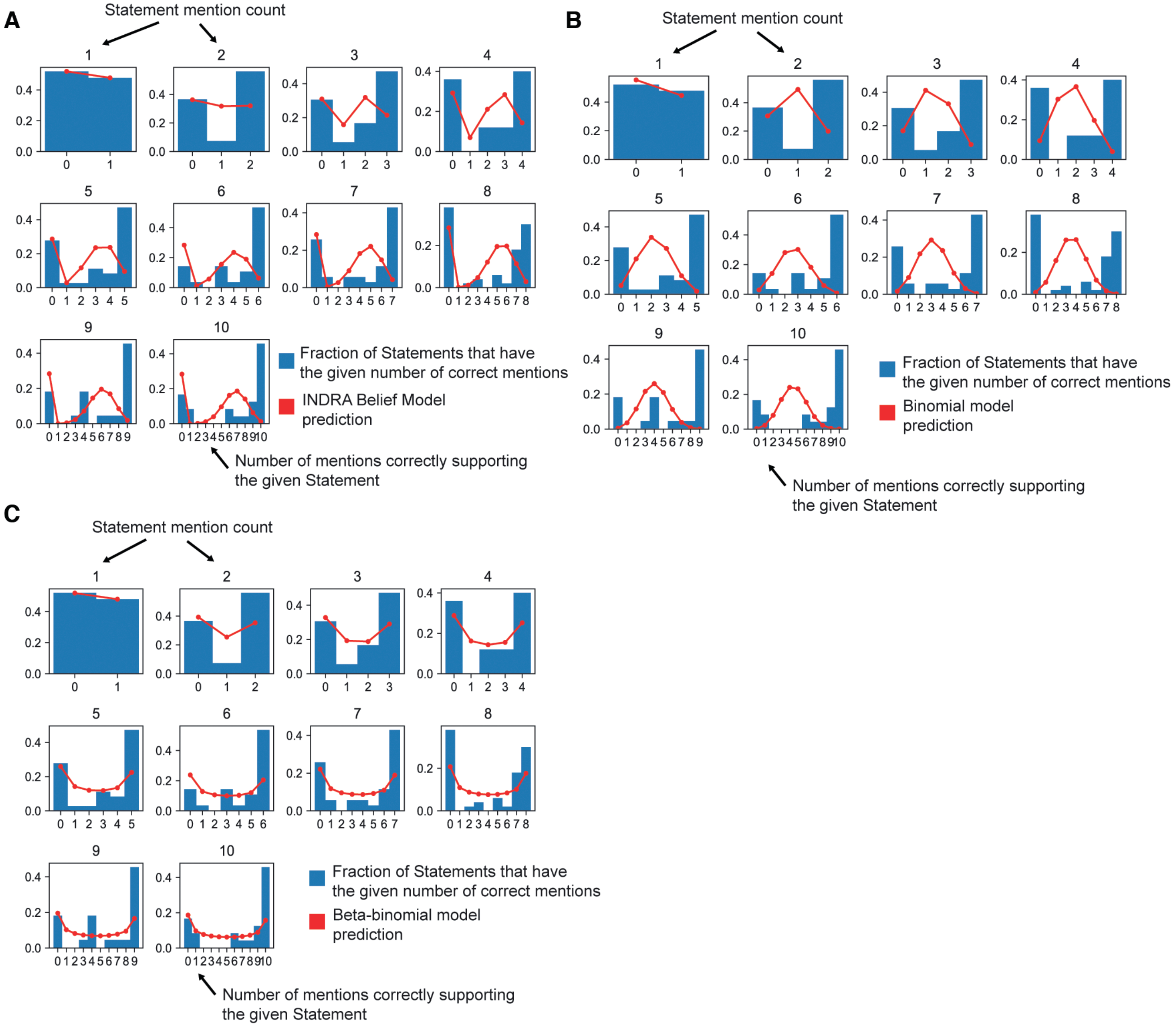

Figure EV3. Observed and predicted distributions of mentions correctly extracted by Reach for Statements supported by up to 10 Reach mentions.

A Frequencies of correct mentions predicted by the INDRA Belief Model. The blue bars in each subplot show the frequencies of statements with *k* correctly extracted mentions for *n* total mentions for the Statement (considering mentions from the Reach reader only). The red line in each subplot shows the frequencies of correct mentions expected by the INDRA Belief Model. The INDRA Belief Model expects a substantial proportion of Statements to have an intermediate number of correctly extracted mentions, whereas the empirical data suggests that Statements are more likely to be associated with mentions that are either all correct or incorrect.

B Frequencies of correct mentions expected by the Binomial model. Blue bars are identical to (A).

C Frequencies of correct mentions expected by the Beta-binomial model. Blue bars are identical to (A) and (B). The Beta-binomial model differs from the INDRA Belief Model and Binomial models in that it predicts relatively greater proportions of Statements with mentions that are either all correct or incorrect.

© 2023 The Authors

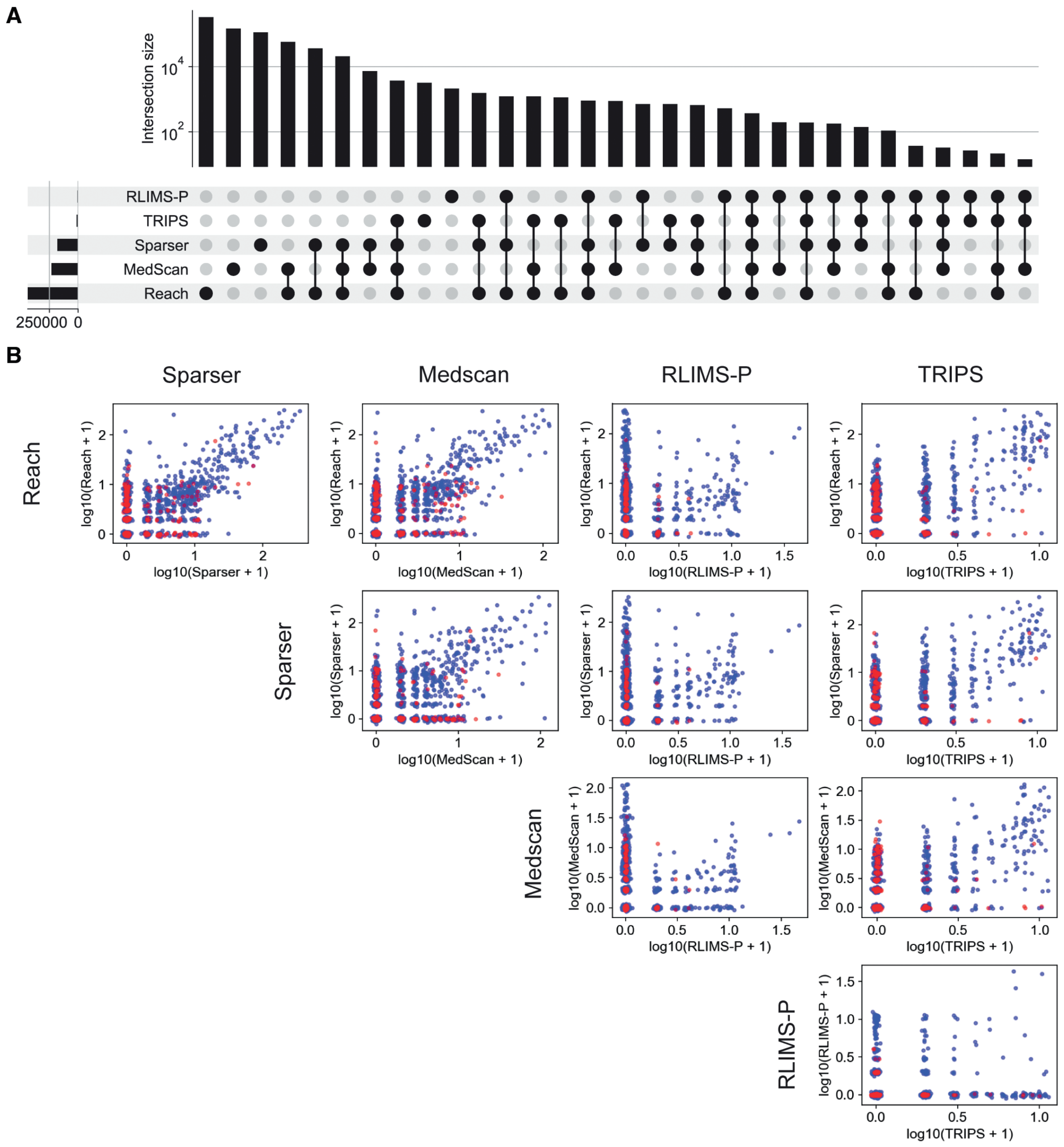

Figure EV4. Reader overlap and Statement correctness.

A Upset plot (equivalent to a Venn diagram with more than three sets) of Statement support for five machine reading systems integrated by INDRA. Data are identical to Fig 5A but intersection sizes are plotted on a log scale and all 32 possible reader combinations are shown.

B Multi-reader mention counts and Statement correctness. Each subplot shows the relationship between mention counts from a combination of two readers for manually curated Statements. Blue points represent Statements that were curated as correct; red points were curated as incorrect. A small amount of random jitter has been added to each point to indicate the density of points with fewer mention counts.

 © 2023 The Authors
