# Supplementary material for: Automated assembly of molecular mechanisms at scale from text mining and curated databases
Source: Mol Syst Biol. 2023 Mar 20;19(5):e11325. doi: 10.15252/msb.202211325 (PMC10167483; doi:10.15252/msb.202211325)
Supplement: Supplementary file 5 [file MSB-19-e11325-s001.pdf]

molecular systems biology

Automated assembly of molecular mechanisms at scale from text mining and curated databases

John Bachman, Benjamin Gyori, and Peter Sorger
DOI: 10.15252/msb.202211325

Corresponding author(s): Peter Sorger (peter_k_sorger@hms.harvard.edu) , Benjamin Gyori (benjamin_gyori@hms.harvard.edu)

Review Timeline:

Submission Date:	31st Aug 22
Editorial Decision:	24th Oct 22
Revision Received:	22nd Jan 23
Editorial Decision:	24th Feb 23
Revision Received:	24th Feb 23
Accepted:	27th Feb 23

Editor: Maria Polychronidou

Transaction Report:

(Note: With the exception of the correction of typographical or spelling errors that could be a source of ambiguity, letters and reports are not edited. Depending on transfer agreements, referee reports obtained elsewhere may or may not be included in this compilation. Referee reports are anonymous unless the Referee chooses to sign their reports.)

Thank you again for submitting your work to Molecular Systems Biology. I apologise for the delay in getting back to you with a decision, which was due to some delays in receiving the referee reports. We have now heard back from the three reviewers who agreed to evaluate your study. As you will see below, the reviewers think that the study is relevant. However, they raise a series of concerns, which we would ask you to address in a revision.

The recommendations of the reviewers are quite clear and I therefore see no need to repeat any of them here. All issues raised by the referees would need to be satisfactorily addressed. Please let me know in case you would like to discuss in further detail any of the issues raised, I would be happy to schedule a call.

Reviewer #1:

This paper describes the INDRA system for assembling statements about molecular interactions using information extracted from the primary literature and from biological databases. Confidence (belief) scores are assigned to individual statements based on the amount and source of the supporting evidence. Several applications are described including identifying protein-protein interactions that are not captured by the BioGrid database and identifying potential mechanisms to explain gene relationships in the Cancer Dependency Map.

Overall, this is an excellent paper. The authors are very thoughtful about the ambiguities and errors that arise from biomedical text mining and data integration. They go to great lengths to address some of these challenges and, as a result, they end up with a cleaner, less redundant set of mechanistic statements that more accurately capture the underlying biology. For example, they use context to disambiguate terms with multiple possible meanings, they correct common errors in the sequence positions of PTMs, and they use hierarchical relationships (e.g., between genes and families) to combine equivalent statements that vary in their level of detail. Another unique feature of INDRA is the use of multiple text mining systems. This increases the total amount of information extracted and the confidence in individual statements. The paper is very well written.

The paper could be improved by addressing the following points:

1. It would be helpful to briefly describe what relation types are extracted by the five reader systems.

2. Normalization:
-The paper describes how INDRA maps text-mined entities between different identifier types, but how is the initial mapping of the "raw" text description of the entity to any database identifier done? Does INDRA rely on the built-in normalization methods of the individual text-mining tools for this?
-The paper states that INDRA statements can distinguish between different forms of a gene/protein, such as oncogenic vs. wild-type BRAF. Is this information available from all the text-mining tools used? How is this normalization done?
-Finally, the paper states that 38% of statements are filtered out because of failure to normalize one or more of the entities. Are most of these statements errors? If not, can any generalizations be made about the types of cases that are hard to normalize?

3. In the refinement step, is there a concern that a "refined" statement that is an error might take the place of a correct, more general statement? This seems like it could be a valid concern because more general statements are often supported by more evidence than highly specific refined statements.

4. In many cases, the evidence used to curate the information in the databases used by INDRA are likely to be the same papers that the text-mined statements are coming from. Thus, the database and text-mined evidence are not completely independent. Is it possible to estimate to what extent this is true?

5. Comparison to BioGrid:

-While this is an interesting application of INDRA, I am curious why BioGrid was not included as a source database in creating the the Benchmark Corpus.
-The authors note that the likelihood of a statement being corroborated by BioGrid is correlated with belief score. As the authors say, this could be taken as validation of the belief score. However, another possible explanation is BioGrid is more likely to capture an interaction that is mentioned in many papers and the belief score is also partially based on the number of times a statement is mentioned. Did the authors consider this possibility?
-Finally, I am wondering if part of the reason for INDRA detecting ~100K PPIs that are not in BioGrid might be how INDRA and BioGrid define a PPI. For example, enzyme-substrate interactions (such as a kinase-substrate interaction) are sometimes considered PPIs, but the interaction is so transient that it is often not detected by assays designed to detect PPIs (e.g., co-IP). If INDRA has a more expansive definition of a PPI than BioGrid that could account for some of the interactions that are in INDRA but not in BioGrid.

6. How does INDRA handle statements involving more than two entities (such as a complex of more than two proteins)? Specifically, how does INDRA handle information that doesn't fit into a nice, tripartite statement like "A binds B". This is hinted at in Figure 7G, but a better explanation would be helpful.

7. A list of some of the statements that are incorrect in multiple readers would be interesting and helpful to developers of text mining systems.

8. When looking for explanations for DepMap dependencies in the INDRA network, it appears that only direct (one-hop) causal interactions in the INDRA network were considered. (Except for parent-link interactions which are technically multi-step). Is that correct? Did the authors consider multi-step paths (other than parent-link interactions) in the network as explanations?

Minor comments:
1. Supplementary figures are numbered S1, S2, S4, S5 (there is no S3)
2. It would be helpful to mention in the caption of Fig 5A that the complete set of 32 combos is shown in S5A.

Reviewer #2:

Mechanistic information about biological pathways is available at a large scale in publications and databases. Integrating the available information into mechanisms for a comprehensive understanding as well as a basis for further analyses introduces various challenges. The vast amount of available information makes manual integration unfeasible whereas the redundancy, different level of detail and inconsistency complicate automated integration of information from different sources. While NLP tools are capable of extracting mechanistic information from publications, they also introduce an error source that adds on to the inherent error or uncertainty. Both need to be considered to determine the reliability of mechanistic information extracted from literature.

In this manuscript, the authors extend their own software by improving the possibility to take publications as an input. This is achieved by combining (several) reading systems for extracting and assembling information. The combination of information from databases and literature at a large scale is achieved by using an intermediate formal representation of statements and establishing relationships between them, thus addressing redundant and incoherent information. The reliability of extracted information is modeled by taking into account the amount of supporting evidence. Different models for reliability analysis are compared. The authors apply their pipeline to generate a corpus of extracted mechanisms and use it to validate their approach on two applications. They demonstrate the potential for complementing the BioGRID database by suggesting new interactions and refining known PPIs as well as adding evidence from primary resources. Furthermore, they showcase its use for interpreting co-dependencies in a large gene expression data set and supply evidence for the added value of information from text mining over database entries.

In my opinion this is an important and highly relevant study. Yet, there are a few things which would strengthen the manuscript further.

Major
1. As stated in the Introduction, "the creators of Pathway Commons [...] have estimated that their resource covers only 1-3% of the available literature". I'm wondering whether the proposed approach can exceed that and how much of the information encoded in a manuscript can be automatically extracted. To evaluate this, the authors could carefully curate a few manuscripts and assess which fraction of the manually extracted information is picked up by their tool.

2. The extraction from databases and text mining (using several parsers) were already introduced by the authors for PTMs: "Assembling a phosphoproteomic knowledge base using ProtMapper to normalize phosphosite information from databases and text mining. John A. Bachman, Benjamin M. Gyori, Peter K. Sorger bioRxiv 822668; https://doi.org/10.1101/822668". It would be good to mention this and to clarify the novelty of the presented results.

Minor
3. Since the belief model makes up a significant part of the results, it should be motivated in the Introduction, contextualized and contrasted with the Status Quo explicitly.

4. Abstract and Introduction mainly indicate that this would be an INDRA application paper. That makes it less intuitive, why the INDRA methodology and formalism (evidence, statements, ...) are explained in so much detail, especially in Figures 1, 2, 3.

5. References to Methods should go to specific sections.

6. p4 "at literature scale" unclear. "Scale" is used several times throughout the paper without a precise definition.

7. Fig. 1A context in the Results text (p.6) is different from the description text. Does not give an intuition for understanding the "series of interconnected issues" mentioned on p.6.

8. Analogy of fragment assembly and sequencing reads does not hold for different levels of specificity. Fig. 3A and B provide a much better intuition for fragment relationships.
Same case on p.11: Analogy seems superfluous because the concepts are explained clearly without it.

9. p.7 "Our preliminary studies identified multiple technical and conceptual problems ...": What studies is this referring to? These issues have been laid out in the previous INDRA publication, so would be appropriate to reference that and also to use in Introduction.

10. Please clarify if Figure 1 is specific for the pipeline presented here or part of the standard INDRA architecture. If the latter, might be more suitable in the Methods section.

11. p.8 "this used the previously described extraction logic (Gyori et al., 2017) but extended to multiple additional sources including SIGNOR (Perfetto et al., 2016)" please explain. Are you referring to ordering logic as in Fig. 3? Is this extension part of the results presented here?

12. Fig 2A : Please highlight the parts which were already in place and the new parts.

13. p.8 "After collecting information from each source, a series of normalization and filtering procedures were applied (green and red boxes, Figure 2A)": Which steps from Fig. 2 are normalization? Ideally reference the Fig. 2A step that is referred to in the following Result sections and use the terms consistently between Figure, Figure description, Results text (e.g. p.9 "sequence normalization" not in Figure 2).

14. p.9 The order of the pipeline steps in text and figure seem to differ, in particular "Map sites of PTMs" and "Filter to grounded only". Please clarify and ideally homogenize.

15. p.10 last paragraph: Linking database entries to text is a great result and makes sense to introduce at this point. Yet, the text jumps between explaining the pipeline, explaining an application and going back to introducing the pipeline. Going back and forth makes it more difficult for the reader to follow the argumentation.p.13/Fig 4A: Please clarify "strength" of a study and Fig. 4A "relative influence".

16. p.13: Is reliability estimation a standard procedure in a pipeline like the one presented here? If not, where has it been done before? What is the standard model, if there is one? What motivates the use of the structured probability models? This extensive part of the results is not expected from the Introduction.

17. p.14: Choice of different approaches to reliability analysis is motivated here, but reliability estimation in general is not explicitly motivated in Introduction or introduced as a standard part of such a pipeline. However, necessity is already justified by integrating different readers and motivated by technical error, could just build on that.

18. p.13/14 "Curation involved determining whether a given mention correctly supported a specific Statement based on human understanding of the sentence containing the mention and the overall context of the publication (see Methods)": Is this missing from Methods?

19. Table 2: Table header (1-10) refers to number of mentions?

20. p.14 "Each of the three models was independently fitted to data from the Curated Corpus using Markov chain Monte Carlo optimization (see Methods)". This seems to be missing in the methods section. Furthermore, I would like to mention that Markov chain Monte Carlo methods are not optimization but sampling approaches.
Figure 7F: "[...] explanation patterns shown in panel D" should be panel C

21. p.26/27 last paragraph: Do additional statements show a similar distribution of beliefs as corpus from main analysis?

22. p.33 last line and p.34 4th line reference Table 3 but mean Table 6

Reviewer #3:

Summary

This paper describes the creation of workflow for computational reading of biomedical literature. This workflow was based on six separate computational readers, and included many important technical steps including removal of hypothesis statements, entity normalization/linking, sequence normalization, and deduplication. This paper includes two quite innovative sections - an approach to hierarchically organizing relationships based on whether one statement refines another, and the construction of a model to assess the reliability of statements based on mention counts from each reader. Finally, the paper describes several approaches to evaluate the performance of their method based on comparisons to manual curation, curated resources, and DepMap data.

Major comments

* Improve data availability: Availability of the Benchmark Corpus is important for both reproducibility of the manuscript analysis and for downstream reuse by others. Currently the Benchmark Corpus is distributed as a .pkl file, and reading that file appears to be nontrivial. This reviewer spent ~30 minutes attempting various methods to read the file, including using the pklload function (error on missing config.json file) and pickle.load (errors on installation of the indra module). It would be much more useful for the authors to distribute the Benchmark Corpus in some plain text format and through some recognized data repository (e.g., zenodo, figshare). The ability for readers to easily inspect the Benchmark Corpus is particularly important given the extremely high AUPRC values in Table 6.

Minor comments

* The manuscript is quite long, to the point that the key messages may get lost to less focused readers. The authors are in the best position to decide what, if anything, could be removed or moved to supplemental materials while still preserving the overall intent. (This reviewer suggests Figure 1 could be a candidate.)
* A clearer description of the data model behind a Statement would be useful. Both Box 1 and Figure 3A seem to have this intent, but they differ in enough ways that make it unclear to the reader (e.g., "Entities" vs "Agents").
* (Pages 8-10) Each normalization and filtering step is important, and they could be described in much more detail. If those methods are described in prior work, they could be cited. If not, more details should be added to the Methods or Supplementary Methods.
* Figure 4D: If space permits, changing the ambiguous "Belief" label in the legend to "INDRA Belief" or "INDRA Belief Model" would be clearer.
* The Discussion could benefit from a bit more contextualization of the results in the broader space of knowledge graph construction. With AUPRCs of > 0.9, has the problem of automatically building these KGs at massive scale a solved issue then? Are there caveats in the results that may temper enthusiasm? (For example, could the selection of the negative examples in the Curate Corpus based on incorrect statements from the readers be somehow leading to an overestimate of the performance? Or the fact that the positive examples were based on results from individual readers?)

Detailed Response to Reviewer Comments
MSB-2022-11325

Reviewer #1:

This paper describes the INDRA system for assembling statements about molecular interactions using information extracted from the primary literature and from biological databases. Confidence (belief) scores are assigned to individual statements based on the amount and source of the supporting evidence. Several applications are described including identifying protein-protein interactions that are not captured by the BioGrid database and identifying potential mechanisms to explain gene relationships in the Cancer Dependency Map.

Overall, this is an excellent paper. The authors are very thoughtful about the ambiguities and errors that arise from biomedical text mining and data integration. They go to great lengths to address some of these challenges and, as a result, they end up with a cleaner, less redundant set of mechanistic statements that more accurately capture the underlying biology. For example, they use context to disambiguate terms with multiple possible meanings, they correct common errors in the sequence positions of PTMs, and they use hierarchical relationships (e.g., between genes and families) to combine equivalent statements that vary in their level of detail. Another unique feature of INDRA is the use of multiple text mining systems. This increases the total amount of information extracted and the confidence in individual statements. The paper is very well written.

The paper could be improved by addressing the following points:

1. It would be helpful to briefly describe what relation types are extracted by the five reader systems.

This is an excellent suggestion. We have added a new Expanded View Table EV1 that shows how many of each INDRA Statement type was extracted by each reading system from the Benchmark Corpus document set.

More generally, the reading systems used in this study were developed with a primary focus on extracting physical interactions and causal relations at the molecular level. The Reach, Sparser, MedScan and TRIPS systems can extract a wide variety of relationship types including binding/complex formation, catalytic activation and inhibition, various forms of post-translational modifications and their reverse, transcriptional regulation, etc. In contrast, RLIMS-P is limited to extracting phosphorylation relations, and the ISI/AMR system extracts only complex formation. It is worth noting that not all relationships extracted by reading systems are currently ingested by INDRA. For example, MedScan produces a “CellExpression--surface” relation type which represents protein expression and doesn’t map onto existing INDRA Statement types. In the future, it would

be possible to extend the range of INDRA Statement types to incorporate a wider range of biological processes and potential mechanisms that can be extracted from text.

2. Normalization:

-The paper describes how INDRA maps text-mined entities between different identifier types, but how is the initial mapping of the "raw" text description of the entity to any database identifier done? Does INDRA rely on the built-in normalization methods of the individual text-mining tools for this?

In response to this question we have extended the Methods section on "Text mining of article corpus" to clarify how named entity recognition and normalization were performed.

To summarize, all of the reading systems in this study have built-in approaches to named entity recognition, a process that aims to tag words that represent biological entities to differentiate them from general English words. This process is tightly integrated with each system's parsing algorithms. In addition to the recognition of named entities, five of the six reading systems (Reach, Sparser, TRIPS, MedScan, and RLIMS-P) also perform named entity *normalization*, a process by which an identifier is assigned to a string recognized as a named entity. In the case of the ISI/AMR system, INDRA relies on our Gilda software (Gyori et al., 2022) to perform named entity normalization based on the strings tagged as named entities by the system. In addition, as we describe in the manuscript, INDRA uses grounding mapping as well as context-dependent disambiguation to override the results of named entity normalization provided by reading systems in cases when an error is detected.

-The paper states that INDRA statements can distinguish between different forms of a gene/protein, such as oncogenic vs. wild-type BRAF. Is this information available from all the text-mining tools used? How is this normalization done?

INDRA Statements can represent multiple types of states on protein Agents: post-translational modifications, mutations, cellular location, activity, and bound conditions (see (Gyori et al., 2017)). This is sufficient to represent information on oncogenic variants, for example. However, reading systems differ in their ability and approaches to extracting the necessary information to specify this type of context; not all systems can extract all forms of *Agent* states. For example, with respect to gene mutations, the RLIMS-P, TRIPS and Reach systems can extract mutation states of Agents while the ISI/AMR and Sparser systems cannot. Individual reading systems also have their own pattern-matching logic by which mutations (typically point mutations) are extracted. These typically rely on a combination of regular expressions for known amino-acid residue names (e.g., Val, V, Valine) and numbers appearing in canonical patterns such as "V600E". In the case of Reach, extraction rules for detecting mutations can be examined at:
https://github.com/clulab/reach/blob/master/main/src/main/resources/org/clulab/reach/biogramm

ar/entities/mutants.yml. More information on this is also available in (Valenzuela-Escárcega et al., 2018).

-Finally, the paper states that 38% of statements are filtered out because of failure to normalize one or more of the entities. Are most of these statements errors? If not, can any generalizations be made about the types of cases that are hard to normalize?

The question of Agents that are not normalized (i.e., entities that don't have any identifiers assigned to them; we also refer to these as "ungrounded") by reading systems is an interesting issue that we have previously studied in some detail. We have found that Statements containing unnormalized Agents do not necessarily arise from errors in extraction, as explained in more detail below. However, given that the identity of these entities is unknown, they don't meaningfully contribute to the applications we present in the current study; therefore we filter them out.

In our previous study (Bachman et al., 2018) we performed a systematic analysis of entities that were unnormalized by the Reach reader. When we examined the distribution of unnormalized entities by frequency, we found the following patterns of issues:
Protein families and complexes: a large proportion of unnormalized entities represented protein families and/or multi-protein complexes that could not be grounded in conventional protein/gene centric databases such as UniProt and HGNC. A typical example is the well-known "AP-1" transcription factor. **Prefixes and suffixes**: we also found that another common issue was the use of prefixes and suffixes with gene names that prevented the matching of entity texts to database entries. These included gene fusions such as ("<gene>-GST", "eGFP-<gene>"), experimental perturbations ("<gene>-KO", "si<gene>"), species indicators ("mmu-<gene>"), and a wide variety of other gene modifiers. **Incorrect entity boundaries**: Among the remaining issues, we have found that the named entity recognition step in the reading systems we use can identify incorrect boundaries for an entity, leading to a failure to match the captured entity text to a database (for example, a reader extracts only "PI" in "PI 3-kinase"). This can lead to concepts that are too generic to be meaningfully grounded, like "receptor" or "antigen". On the other hand, inclusion of too much text surrounding an entity can also lead to mismatches. **Entity types not covered by named entity resources**: Other cases involve entities that represent concepts entirely outside the scope of the knowledge bases used for normalization: for example "DC" (dendritic cell) or "CD4+" (a T-cell subtype) – concepts absent from gene/protein databases. Further into the long tail of unnormalized entities we find that readers are sometimes stymied by stray or non-standard punctuation, author spelling errors, etc.

The FamPlex resource (Bachman et al., 2018) we created based on the above insights tackles many of the issues detailed above and has since been integrated with Reach and other reading systems. In light of this, it is interesting to examine what the most common source of

unnormalized entities is in the context of the current study. Therefore, in response to the reviewer's question, we have compiled statistics on the most frequently occurring texts corresponding to Agents lacking identifiers in the 6,429,134 Statements in the pipeline prior to the "*Filter to grounded only*" step in INDRA (see **Figure 2A**). We found that the most commonly occurring unnormalized Agent texts were generic, high-level terms such as "expression", "activity", "cytokine", "growth" and "signaling", consistent with our previous finding that ungrounded entities often correspond to generic concepts lacking context (we added a sentence to the manuscript about this observation). While in some applications, Statements over Agents representing these terms could be valuable—in fact, our future work involves generalizing INDRA to representing high-level terms such as "tumor growth", and "overall survival"—they would not meaningfully contribute to the applications presented in this work.

3. In the refinement step, is there a concern that a "refined" statement that is an error might take the place of a correct, more general statement? This seems like it could be a valid concern because more general statements are often supported by more evidence than highly specific refined statements.

The reviewer is correct: statements that are more specific due to the presence of more extracted context (for example, a *Phosphorylation* statement with only a substrate vs. a related statement with a mutated enzyme, substrate, position, and residue) can be subject to additional errors because there are more things that can go wrong in extraction. However, INDRA includes a principled solution to this. Namely, *Evidences* are passed from more specific to more general Statements (not the other way around), and therefore more general Statements will inherently have higher belief. By applying a belief cutoff it is possible to eliminate a highly-specific *Statement* with lower belief and so the *remaining* most specific Statement will be more likely correct.

To make this clearer, we added the following text to the Results in the section "*Two approaches to modeling the reliability of Statements from multiple readers*": "*Because mentions from more specific Statements flow to more general ones but not the reverse, the belief estimates for the most specific Statements are determined only by their directly supporting evidence. This leads to an overall inverse relationship between specificity and belief that allows Statements to be filtered to the most specific statement lying above a certain threshold of belief, thereby excluding potentially unreliable and highly specific Statements in which extracted details may reflect technical errors rather than meaningful additional context.*"

4. In many cases, the evidence used to curate the information in the databases used by INDRA are likely to be the same papers that the text-mined statements are coming from. Thus, the database and text-mined evidence are not completely independent. Is it possible to estimate to what extent this is true?

This is an interesting question that can be addressed because INDRA tracks the article source (by PMID) of every database interaction and text-mined mention supporting a Statement. We used this data from the Benchmark Corpus to determine the overlap of the article PMIDs from database sources vs. text mined sources and obtained the following results:

- 15,729 PMIDs for database interactions only
- 220,210 PMIDs for text mined interactions only
- 23,292 PMIDs for both database and text mined interactions (10% of total text mined PMIDs; 60% of total database PMIDs)

These results show that for the Benchmark Corpus, text mining draws from a much larger number of unique articles than the databases included in the Benchmark Corpus, and that articles covered by both types of sources account for a large proportion of the curated database content (60%) but a much smaller proportion of the articles drawn on by text mining (10%).

Of course, we would not expect the articles for databases vs. text mining to be disjoint/independent, since they are drawing on the same overall pool of published literature on biological mechanisms. Nor would independence necessarily be desirable: as discussed in the paper, even in cases in which text mining systems re-extract interactions that have already been included in databases, the text evidence and article references they supply add valuable additional context. The fact that text mining systems capture information from many of the same articles that have already been targeted for curation by human curators supports the idea that automated systems are focusing on important and relevant information. At the same time, the fact that text mining can draw on a much larger and more diverse pool of articles than databases confirms that text mining can greatly scale up mechanistic curation relative to purely manual efforts.

5. Comparison to BioGrid:

-While this is an interesting application of INDRA, I am curious why BioGrid was not included as a source database in creating the the Benchmark Corpus.

INDRA can, in fact, take BioGRID as input and extract Complex Statements corresponding to PPIs represented in BioGRID (see module documentation at https://indra.readthedocs.io/en/latest/modules/sources/biogrid/index.html). However, in order to benchmark INDRA with respect to BioGRID as a structured resource (Figure 6), we excluded BioGRID from assembly so as to avoid any “leak” of information, for instance at the level of beliefs. Given that the DepMap analysis (Figure 7) also relies on comparisons to BioGRID, we did not include it as part of the INDRA-assembled network in this case either.

-The authors note that the likelihood of a statement being corroborated by BioGrid is correlated with belief score. As the authors say, this could be taken as validation of the belief score. However,

another possible explanation is BioGrid is more likely to capture an interaction that is mentioned in many papers and the belief score is also partially based on the number of times a statement is mentioned. Did the authors consider this possibility?

We did consider this important point. Redundancy at the level of distinct papers/sentences (due to their diversity) is key for belief estimation. This is then of course also correlated with canonicalness, i.e., how often something was observed, which again is correlated with the strength and universality (occurring in many different contexts) of a given interaction. Such robust interactions would also be more likely to be curated in BioGRID. However, systematic reading errors can also produce *incorrect* Statements with many (incorrectly processed) supporting mentions, and hence high belief scores. The comparison with BioGRID shows that this is not a dominating factor.

-Finally, I am wondering if part of the reason for INDRA detecting ~100K PPIs that are not in BioGrid might be how INDRA and BioGrid define a PPI. For example, enzyme-substrate interactions (such as a kinase-substrate interaction) are sometimes considered PPIs, but the interaction is so transient that it is often not detected by assays designed to detect PPIs (e.g., co-IP). If INDRA has a more expansive definition of a PPI than BioGrid that could account for some of the interactions that are in INDRA but not in BioGrid.

In the case of the BioGRID analysis, we considered only INDRA Statements of type “Complex”, which are specifically constructed from mentions in text with trigger words denoting complex formation. We did not interpret Statements about post-translational modifications or regulation of amount or activity as PPIs. In principle, many of these alternative types of Statements that are picked up by INDRA could be interpreted as PPIs and used to enrich databases like BioGRID, but in practice it is difficult to determine from text mining alone which of these represent direct interactions as opposed to indirect regulatory influences.

6. How does INDRA handle statements involving more than two entities (such as a complex of more than two proteins)? Specifically, how does INDRA handle information that doesn't fit into a nice, tripartite statement like "A binds B". This is hinted at in Figure 7G, but a better explanation would be helpful.

The INDRA *Complex* Statement can contain an arbitrary number of Agent members. Complexes having more than two members are extracted by text mining systems in practice, and are also curated in some pathway databases (e.g., Reactome). At the level of INDRA Statements, and in the Benchmark Corpus, these multi-member complexes can be represented directly. How such interactions are then handled in downstream analysis derived from INDRA Statements is an application-specific choice. For the purposes of network assembly—as in the case of the DepMap analysis in Figure 7—these multi-member complexes are “binarized” such that an edge is

introduced between each pair of members within the complex. In the case of this network analysis, we chose to only expand complexes up to 3 members and throw away ones with more members, for two principal reasons. First, text-mined *Complex* Statements containing many members are often incorrect, erroneously including both binding and non-binding proteins that are co-mentioned in the same sentence. Second, when represented in a network, a complex with *n* members expands to $n*(n-1)/2$ binary edges among its members, which can lead to many false positive causal explanations (especially because many large complexes are spurious, as noted).

7. A list of some of the statements that are incorrect in multiple readers would be interesting and helpful to developers of text mining systems.

This is a valuable suggestion. We examined a number of these Statements and added the following paragraph to the manuscript:

> To characterize systematic issues affecting multiple readers, we also examined sentences associated with Statements that were incorrectly extracted by more than one reader. Recurring errors included misgrounding due to overlapping aliases (e.g., grounding – by four readers - of "TPP1" to gene TPP1 rather than gene ACD for which "TPP1" is an alias), incorrect extraction of negative results (e.g., "*our preliminary attempts have not identified direct phosphorylation of PPARγ by MST2*", extracted by three readers as a *Phosphorylation* statement in which MST2 modified PPARγ), unrelated subclauses being causally linked (e.g., "*quiescent cells attenuate eIF2α phosphorylation and induction of the ER stress proapoptotic gene GADD153*" incorrectly extracted by three readers as a phosphorylation of GADD153 by eIF2α), incomplete named entity recognition (e.g., "*Shc associates with epidermal growth factor (EGF) receptor*", incorrectly extracted by two readers as binding between Shc and EGF, not EGFR), and extraction of protein-DNA binding as protein-protein binding (phrases similar to "*c-Jun binds to AP-1 sites to regulate gene expression*" incorrectly extracted by four readers as binding between c-Jun and the AP-1 complex, which includes c-Jun as a component). In many of these cases, human readers are able to recognize subtleties in the language that are difficult for machines to parse correctly.

8. When looking for explanations for DepMap dependencies in the INDRA network, it appears that only direct (one-hop) causal interactions in the INDRA network were considered. (Except for parent-link interactions which are technically multi-step). Is that correct? Did the authors consider multi-step paths (other than parent-link interactions) in the network as explanations?

The reviewer is correct - the approach that we took focused on one-step causal interactions. We agree that considering multi-step paths is a promising future research direction. However, the approach by which multi-step paths are constructed requires non-trivial methodological advances. For example, one has to ensure that the paths are causally sound, i.e., that they actually represent

a valid sequence of biochemical events. Different modeling formalisms and network types could be considered for this task with different causal properties. For example, paths found to explain DepMap codependencies on an undirected, unlabeled graph whose edges are derived from INDRA Statements will have different properties from paths on a labeled, directed graph that takes into account INDRA Statement type and directionality. To clarify this point, we added the following section to the Results:

> For the purpose of this analysis, we did not consider multi-step causal paths between genes in the network to be explanatory. This is due to the challenge of ensuring that sequences of edges in the network represent causally linked biochemical events rather than unrelated associations, which would lead to false-positive explanations. Capturing and representing information about causal transitivity in biological networks is the subject of ongoing research (Fig 4A, "causal transitivity" in lower-right quadrant).

Minor comments:

1. Supplementary figures are numbered S1, S2, S4, S5 (there is no S3)

In the original manuscript, our numbering of supplementary figures followed the numbering of the main figures. For instance, Figure S1 corresponded to Figure 1, Figure S4 to Figure 4, and so on. In the revised manuscript, supplementary figures are now using the required "extended view" nomenclature and have been renumbered as Figure EV1, EV2, EV3 and EV4, respectively.

2. It would be helpful to mention in the caption of Fig 5A that the complete set of 32 combos is shown in S5A.

We amended the caption for Fig 5A to refer to EV4A (what used to be S5A), as suggested by the reviewer.

Reviewer #2:

Mechanistic information about biological pathways is available at a large scale in publications and databases. Integrating the available information into mechanisms for a comprehensive understanding as well as a basis for further analyses introduces various challenges. The vast amount of available information makes manual integration unfeasible whereas the redundancy, different level of detail and inconsistency complicate automated integration of information from different sources. While NLP tools are capable of extracting mechanistic information from publications, they also introduce an error source that adds on to the inherent error or uncertainty. Both need to be considered to determine the reliability of mechanistic information extracted from literature.

In this manuscript, the authors extend their own software by improving the possibility to take publications as an input. This is achieved by combining (several) reading systems for extracting

and assembling information. The combination of information from databases and literature at a large scale is achieved by using an intermediate formal representation of statements and establishing relationships between them, thus addressing redundant and incoherent information. The reliability of extracted information is modeled by taking into account the amount of supporting evidence. Different models for reliability analysis are compared. The authors apply their pipeline to generate a corpus of extracted mechanisms and use it to validate their approach on two applications. They demonstrate the potential for complementing the BioGRID database by suggesting new interactions and refining known PPIs as well as adding evidence from primary resources. Furthermore, they showcase its use for interpreting co-dependencies in a large gene expression data set and supply evidence for the added value of information from text mining over database entries.

In my opinion this is an important and highly relevant study. Yet, there are a few things which would strengthen the manuscript further.

Major

1. As stated in the Introduction, "the creators of Pathway Commons [...] have estimated that their resource covers only 1-3% of the available literature". I'm wondering whether the proposed approach can exceed that and how much of the information encoded in a manuscript can be automatically extracted. To evaluate this, the authors could carefully curate a few manuscripts and assess which fraction of the manually extracted information is picked up by their tool.

The 1-3% estimate from Valenzuela-Escarcega et al. (2018) is meant to capture the fact that only a small subset of publications and a small subset of information relevant for pathway mechanisms from those publications have been curated by experts into databases so far. Our results show that INDRA's assembly approach–which relies on combining mentions supporting a given interaction from *multiple publications*–is able to yield as-yet uncurated mechanisms with high confidence. Moreover, INDRA is much more comprehensive in curating all of the papers in which a particular mechanism has been identified, thereby providing a means to estimate confidence in the mechanism as well as relevant contextual information. In this sense, INDRA substantially increases coverage of the literature with respect to both known and new mechanisms.

However, the above considerations are different from what the reviewer also asks – namely the fraction of information in a paper understandable by humans that can be extracted automatically from any specific *single* publication, whether by a specific reading system or by INDRA assembling the results of multiple reading systems processing the same article. In the context of the DARPA Big Mechanism program (Cohen, 2015), a team of expert bio-curators from The MITRE Corporation performed an independent evaluation of this task (Peterson et al., 2022). Three MITRE scientists

curated a set of 10 papers for key mechanistic findings, and then reached consensus on which interactions should be in a “reference set”. Remarkably, only 57% of the interactions in the reference set were identified independently by all three scientists, demonstrating that even expert human curators can disagree on what mechanistic information should be captured from a specific manuscript. The output of multiple systems developed in the program were then evaluated for their ability to reproduce entries of the reference set and to calculate the reference set overlap, which is a measure similar to recall. The evaluators found that INDRA, which at that time used only the Reach, TRIPS and Sparser reading systems, was able to identify 76% of the phosphorylation and binding interactions in the reference set. This constituted the highest reference set overlap among participants in the evaluation. These results serve as an adequate and independent (i.e., performed by experts other than the authors) measure of the question raised by the reviewer, and we have not performed additional curation as part of the current revision (the 57% inter-curator agreement for humans raises the question whether a single group can adequately evaluate itself on this task). It is useful to note in this context that INDRA’s approach to belief estimation makes it possible to vary the tradeoff between precision and recall, providing a way to extract information tuned for different downstream use cases.

2. The extraction from databases and text mining (using several parsers) were already introduced by the authors for PTMs: "Assembling a phosphoproteomic knowledge base using ProtMapper to normalize phosphosite information from databases and text mining. John A. Bachman, Benjamin M. Gyori, Peter K. Sorger bioRxiv 822668; https://doi.org/10.1101/822668". It would be good to mention this and to clarify the novelty of the presented results.

We would like to point out that the reviewer is referencing a preprint that we do not currently have in review. In this preprint, INDRA was used to run several reading systems to obtain information about phosphorylation sites and the kinases that modify them. The focus of the study was to demonstrate and attempt to overcome widespread inconsistency in residue positions for post-translational modifications in both the primary literature and in databases. It proposes the ProtMapper tool using which we show how it is possible to solve this specific problem.

Because INDRA was used to obtain the corpus of interactions that motivated the requirement for ProtMapper it conceptually precedes the ProtMapper preprint. At the same time we would also like to note that the ProtMapper preprint does not address any of the issues around knowledge assembly or estimation of statement reliability that are the core of this manuscript and there is therefore no impact on the novelty of our findings. We have since uploaded a revision to our earlier preprint available at https://www.biorxiv.org/content/10.1101/822668v4 (Bachman et al., 2022) that makes this relationship more clear.

Based on this, the appropriate place to refer to the ProtMapper is in the section describing the normalization of protein sequence positions and we cite the updated preprint Bachman et al. (2022) there as a source of the methodology used to perform that subtask.

Minor

3. Since the belief model makes up a significant part of the results, it should be motivated in the Introduction, contextualized and contrasted with the Status Quo explicitly.

This is a good suggestion. To address it we have added the following paragraph to the introduction:

> A key requirement for the broader use of text mining in biological data analysis is overcoming the relatively low technical precision of current systems. One way to mitigate the effect of text mining errors is to filter out low-confidence extractions based on reliability estimates. General reliability estimates can be derived a priori from the published precision scores for specific text mining systems (see, e.g., Valenzuela-Escárcega et al, 2018; Torii et al, 2015), but these figures do not account for the fact that error rates can differ substantially for different types of information or sentence structures. An alternative approach is to cross-reference text mined information against previously curated databases (Holtzapple et al, 2020) which yields high-confidence interactions at the expense of the breadth provided by text mining. For single reading systems, redundancy among extractions (i.e. extracting the same information repeatedly from different spans of text) has been shown to associate positively with reliability (Valenzuela-Escárcega et al, 2018) but this has not as-yet been quantitatively characterized or used to derive reliability scores. In principle, the integration of multiple distinct reading systems with different types and rates of error could provide the information needed to estimate interaction reliability but this has not been previously explored.

4. Abstract and Introduction mainly indicate that this would be an INDRA application paper. That makes it less intuitive, why the INDRA methodology and formalism (evidence, statements, ...) are explained in so much detail, especially in Figures 1, 2, 3.

The novel methodology implemented in the INDRA system we use in the current work is quite different – and much more advanced – than the one we described in Gyori et. al, 2017. Thus, we found it necessary to describe the operation of the current version of INDRA in the first few figures of the paper. Among other things, the INDRA assembly methodology described in the current work is entirely novel and has no antecedent in our earlier work. It is also worth noting that even in the case of pre-existing input modules, the manner in which these are invoked is different here compared to Gyori et. al, 2017. For example, while TRIPS/DRUM was used to process expert-written natural language sentences in Gyori et. al, 2017, here it is used to process scientific articles

directly. Similarly, while the input module for BioPAX / Pathway Commons was introduced in Gyori et. al, 2017, it was only used for small, targeted queries, not bulk ingestion of the entire database.

To make this distinction more clear, in the revised manuscript, Figure 1A shows the INDRA architecture as of Gyori et. al, 2017 and Figure 1C shows the current architecture in other words, the two are now more clearly separated to avoid confusion. Other panels of Figures 1, 2 and 3 focus on novel aspects of INDRA that were not available as of Gyori et. al, 2017.

5. References to Methods should go to specific sections.

In the revised manuscript we now refer to specific sections of the Methods when referencing them from the main text. We also extended the Methods section in the revision to include further details relevant for our results.

6. p4 "at literature scale" unclear. "Scale" is used several times throughout the paper without a precise definition.

We modified the sentence in p4 to clarify “literature scale” as “*Nevertheless, at the current state of the art, machine reading can extract simple relations (e.g., post-translational modifications and binding and regulatory events) at literature scale (i.e., from a substantial fraction of the body of* $3 \cdot 10^7$ *biomedical publications currently available)*”.

We believe that paragraph 4 of the Introduction: “Overall, what is still needed are computational tools for the large-scale assembly …” describes what we mean by large-scale automated assembly in relation to the scale of pathway database curation efforts and modeling approaches and serves as the basis for further references to scale in the manuscript.

7. Fig. 1A context in the Results text (p.6) is different from the description text. Does not give an intuition for understanding the "series of interconnected issues" mentioned on p.6.

We have revised Figure 1 and the corresponding text as part of this revision to make these points more clear. In the revised manuscript, Figure 1A shows the INDRA architecture as introduced in Gyori et al., 2017 for the task of conversion of curated natural language text to machine readable mechanisms. Figure 1B then provides an illustrative example of the additional challenges involved in the automated assembly of large knowledge bases from curated databases and machine reading systems.

The corresponding sentence now reads:

> *Automated assembly of large knowledge bases from curated databases and machine reading systems raises a series of interconnected issues not arising in the conversion of curated natural language text to machine readable mechanisms (**Fig 1A** shows the INDRA architecture for this simpler task as introduced in (Gyori et al, 2017)). In particular, each*

*source of information yields many mechanistic fragments that capture only a subset of the underlying process, often at different levels of abstraction. For example, […] (**Fig 1B**).*

8. Analogy of fragment assembly and sequencing reads does not hold for different levels of specificity. Fig. 3A and B provide a much better intuition for fragment relationships.

Same case on p.11: Analogy seems superfluous because the concepts are explained clearly without it.

While the reviewer is correct in the strict sense, we have found this analogy to be very appealing for general audiences and we would therefore like to retain it. In particular, we want to make the point to non-experts that knowledge assembly is a distinct and potentially complex task that differs from the extraction of individual pieces of knowledge. Thus, there is at least a partial analogy between the assembly of different levels of specificity INDRA Statements (what the reviewer refers to as "fragments" here) and sequencing reads. Namely, a situation in which one sequencing read aligns with but subsumes another sequencing read (one read is fully contained in the other), is analogous to two INDRA Statements that are matching but one contains additional mechanistic detail compared to the other such as the Statements "MAP2K1 phosphorylates MAPK1" vs "MAPK21 bound to BRAF phosphorylates MAPK1 on T185" in Figure 3B. Overall, we think the short sentence where this analogy is made ("Although the analogy in this case is not perfect, something similar is required in genome assembly – if a shorter sequence is fully contained in a longer sequence, the shorter one is redundant.") is a useful reference back to Figure 1B and overall provides interesting context without detracting from the argument.

9. p.7 "Our preliminary studies identified multiple technical and conceptual problems ...": What studies is this referring to? These issues have been laid out in the previous INDRA publication, so would be appropriate to reference that and also to use in Introduction.

This is a good point, and we have modified the sentence to read "*When attempting to scale the process of assembly from curated natural language to scientific publications, we identified multiple technical and conceptual problems …*". In the previous INDRA publication (Gyori et al, 2017) these problems are only informally mentioned in the Discussion as possible future challenges but were not systematically studied and no relevant data was presented there. Therefore a reference to (Gyori et al, 2017) would not seem to be appropriate in this sentence.

10. Please clarify if Figure 1 is specific for the pipeline presented here or part of the standard INDRA architecture. If the latter, might be more suitable in the Methods section.

Figure 1 shows the generic challenges associated with knowledge assembly and the conceptual overview of the INDRA architecture. It is not specific to the Benchmark Corpus presented in our study, rather, it contributes to the basic introduction of the INDRA system and the approach taken

to address the assembly challenge. The pipeline specific to assembling the Benchmark Corpus is shown in Figure 2A. We believe that Figure 1 helps explain the general concept behind the specific instance of a pipeline we present in Figure 2 and makes it clear that pipelines can be customized through reusable software modules. We have therefore elected to keep Figure 1 and its panels in the main text.

However, to address this point, we have improved Figure 1 in multiple ways: we made Figure 1A reflect more closely our prior work on INDRA (Gyori et al., 2017) with Figure 1B presenting the assembly challenge through a conceptual analogy to genome assembly (justified above) and Figure 1C the significantly extended and generalized INDRA assembly process the current work presents to address the assembly challenge.

11. p.8 "this used the previously described extraction logic (Gyori et al., 2017) but extended to multiple additional sources including SIGNOR (Perfetto et al., 2016)" please explain. Are you referring to ordering logic as in Fig. 3? Is this extension part of the results presented here?

Here, "extraction logic" refers specifically to the process by which INDRA processes content from structured sources such as Pathway Commons (BioPAX) and BEL. These two specific input modules were presented in (Gyori et al., 2017). The current work encompasses many extensions to INDRA so that a wider range of structured sources can be used, including SIGNOR.

This "extraction logic" is unrelated to the equivalence and ordering approach over Statements presented in Figures 2 and 3. To make this more clear, we updated the sentence in the revised manuscript to read "*…this used previously described extraction logic (a means of converting structured information of different types into INDRA Statements) (Gyori et al, 2017) but extended to multiple additional sources including SIGNOR (Perfetto et al, 2016)*".

12. Fig 2A : Please highlight the parts which were already in place and the new parts.

This is not an easy request to fulfill, since the INDRA code base has experienced multiple years of active development between our 2017 publication and the current work. The transition from reading simple declarative text to actual manuscripts is a dramatic one. We have nonetheless added gray shading to nodes in Figure 2A representing input modules in INDRA that already existed as of Gyori et al. (2017). It is important to note, however, that even these modules were used to process qualitatively different content in Gyori et al. (2017) as compared to this study. Namely, in Gyori et al. (2017), the Reach and TRIPS reading systems were used exclusively to process simplified (declarative) English language sentences written specifically to define models. In contrast, in this study, Reach and TRIPS (and other reading systems) are used to process scientific abstracts or full text articles. Similarly, Gyori et al. (2017) discusses processing BioPAX and BEL content, however, these input modules were only queried in a targeted way to obtain small-scale

results. In contrast, in the current study, BioPAX and BEL content from the Pathway Commons database and the BEL Large Corpus, respectively, are ingested in bulk.

13. p.8 "After collecting information from each source, a series of normalization and filtering procedures were applied (green and red boxes, Figure 2A)": Which steps from Fig. 2 are normalization? Ideally reference the Fig. 2A step that is referred to in the following Result sections and use the terms consistently between Figure, Figure description, Results text (e.g. p.9 "sequence normalization" not in Figure 2). 14. p.9 The order of the pipeline steps in text and figure seem to differ, in particular "Map sites of PTMs" and "Filter to grounded only". Please clarify and ideally homogenize.

We thank the reviewer for identifying these issues with Figure 2A and the corresponding text. In the revised manuscript we addressed all these issues as follows.

- We changed the figure panel's coloring to use green for normalization steps and red for filter steps. Based on this, the sentence referred to by the reviewer now reads "*normalization and filtering procedures were applied (green and red boxes, respectively, in Figure 2A*)" making the text clearer and consistent with the figure.

We also improved on a number of inconsistent usages of nomenclature in the figure:

- Changed "Map sites of PTMs" to "Normalize sequence positions"
- Added an explicit node to represent the filtering step following sequence normalization: "Filter out non-canonical sequence positions".
- Changed "Map grounding / filter ungrounded" (here "filter ungrounded" was not an appropriate label) to "Map grounding / disambiguate".
- Changed "Ontology-based ID normalization" to "Normalize identifiers".

Finally, we rearranged the text corresponding to Figure 2A to be consistent with the order of nodes in the figure. In particular, we now describe "Filter to grounded only" in the correct order, after grounding mapping and identifier normalization.

15. p.10 last paragraph: Linking database entries to text is a great result and makes sense to introduce at this point. Yet, the text jumps between explaining the pipeline, explaining an application and going back to introducing the pipeline. Going back and forth makes it more difficult for the reader to follow the argumentation.

We apologize if this was difficult to follow. Our approach to describing the results in this portion of the paper is to introduce steps of the INDRA assembly pipeline while presenting intermediate results relevant at a given point of the pipeline, illustrated by specific examples from the Benchmark Corpus. We feel that Fig 2F serves to illustrate the results of INDRA's approach to combining equivalent Statements by highlighting how database-derived and text mining-derived

Statements are aligned through a specific example. Overall, we feel that this is the most appropriate position to present this example, and therefore decided to keep the order as is. However, we have made numerous small changes to the language to make the section easier to follow (particularly given all the changes to Figure 2A described above).

p.13/Fig 4A: Please clarify "strength" of a study and Fig. 4A "relative influence".

To improve clarity, in the revised manuscript we changed "*strength of a particular study*" to "*strength of evidence supporting the findings of a particular study*". To better describe the right quadrants of Fig. 4A (which includes the "relative influence" phrase), we added the following sentence to the revised manuscript: "*These additional challenges of integrated models include dealing with contradictions between Statements, assessing the relative influence or relevance of multiple Statements in a given context, as well as issues surrounding causal transitivity across multiple Statements combined*."

16. p.13: Is reliability estimation a standard procedure in a pipeline like the one presented here? If not, where has it been done before? What is the standard model, if there is one? What motivates the use of the structured probability models? This extensive part of the results is not expected from the Introduction.

As mentioned in our response to point 3. above, we have extended the introduction to better motivate reliability estimation. To summarize, in our review of prior work on this topic, we found that text mining systems are routinely evaluated for their precision at the level of individual extractions. However, this is meaningfully different from the requirement for reliability estimation at the level of INDRA Statements where one Statement can have support from multiple mentions from one or more different reading systems (and in some cases curated databases). We are not aware of prior work that addresses this problem.

17. p.14: Choice of different approaches to reliability analysis is motivated here, but reliability estimation in general is not explicitly motivated in Introduction or introduced as a standard part of such a pipeline. However, necessity is already justified by integrating different readers and motivated by technical error, could just build on that.

As mentioned in our response to point 3. above, we have extended the introduction to better motivate reliability estimation here.

18. p.13/14 "Curation involved determining whether a given mention correctly supported a specific Statement based on human understanding of the sentence containing the mention and the overall context of the publication (see Methods)": Is this missing from Methods?

We thank the reviewer for flagging this omission. We added a detailed "Statement Curation" section to the Methods describing the process of curation and the sets of curated Statements used

for different analyses. We would also like to note that as part of this revision, we further extended the curation dataset and standardized its usage across the text and analyses to ensure consistency. This resulted in updates to Tables 2, 3, and 6, while the conclusions have not changed.

19. Table 2: Table header (1-10) refers to number of mentions?

We extended the caption for Table 2 to explain the meaning of columns: “Each column shows the number of mentions (between 1 and 10) supporting a given Statement in the curation dataset.”

20. p.14 "Each of the three models was independently fitted to data from the Curated Corpus using Markov chain Monte Carlo optimization (see Methods)". This seems to be missing in the methods section. Furthermore, I would like to mention that Markov chain Monte Carlo methods are not optimization but sampling approaches.

Thank you for pointing this out—we added a section to the methods containing the following description:

> *Parameter estimation for the belief models was performed by affine-invariant Markov Chain Monte Carlo (MCMC) as implemented by the emcee software package (Foreman-Mackey et al, 2013). The likelihood function for each model was derived from the functions B(T) as described above. MCMC was performed with 100 walkers running for 100 burn-in steps followed by 100 sampling steps. Python code implementing the MCMC runs is in the GitHub repository for the paper in modules bioexp/curation/process_curations.py and bioexp/curation/model_fits.py.*

Figure 7F: "[...] explanation patterns shown in panel D" should be panel C

We fixed the reference to panel C in the revised manuscript.

21. p.26/27 last paragraph: Do additional statements show a similar distribution of beliefs as corpus from main analysis?

To test this, we applied the best-performing Random Forest model - as used in Figures 6 and 7 - on (i) the set of Statements involving BRAF in the Benchmark Corpus and (ii) the extended Statement set based on additional literature processing. Since both (i) and (ii) also contain Statements supported by entries in curated databases, we made the assumption that their belief is 1, and we therefore applied the belief model only to those Statements supported by mentions from reading systems. The proportion of Statements that are supported by curated databases is higher in the Benchmark Corpus (around 20%) compared to the extended Statement set (around 10%). This is expected since the additional source of mentions in the extended Statement set comes from text mining. For the remaining Statements which are only supported by text mining, the distribution of beliefs is similar across the two sets of Statements. We provide histograms of the two distributions below:

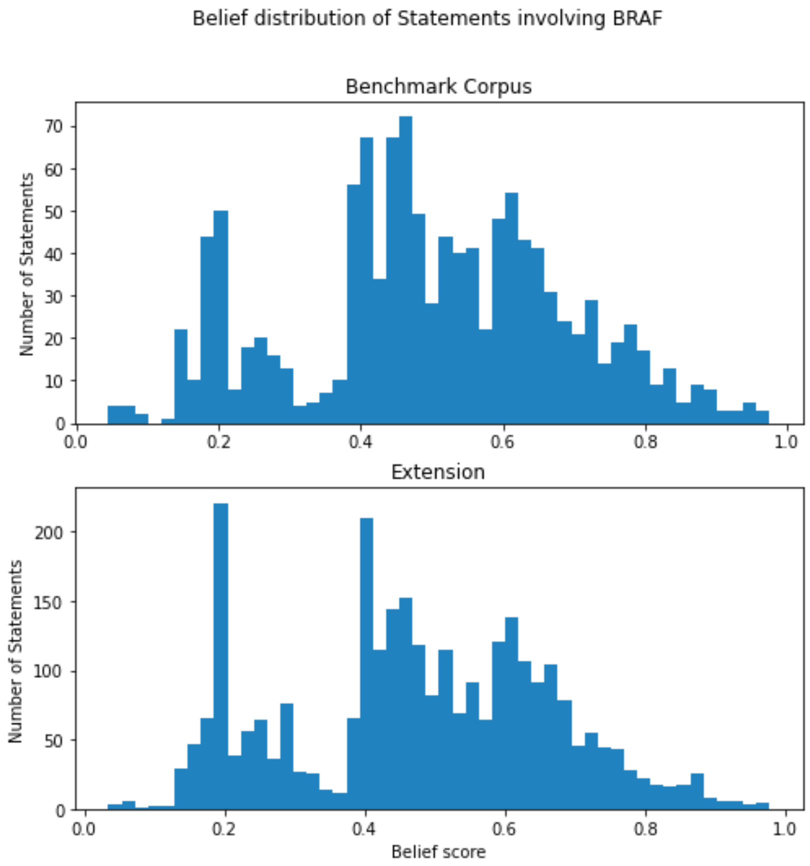

22. p.33 last line and p.34 4th line reference Table 3 but mean Table 6

Thank you—we fixed these references to refer to Table 6 in the revised manuscript.

Reviewer #3:

Summary

This paper describes the creation of workflow for computational reading of biomedical literature. This workflow was based on six separate computational readers, and included many important technical steps including removal of hypothesis statements, entity normalization/linking, sequence normalization, and deduplication. This paper includes two quite innovative sections - an approach to hierarchically organizing relationships based on whether one statement refines another, and the construction of a model to assess the reliability of statements based on mention counts from each reader. Finally, the paper describes several approaches to evaluate the performance of their method based on comparisons to manual curation, curated resources, and DepMap data.

Major comments

* Improve data availability: Availability of the Benchmark Corpus is important for both reproducibility of the manuscript analysis and for downstream reuse by others. Currently the Benchmark Corpus is distributed as a .pkl file, and reading that file appears to be nontrivial. This reviewer spent ~30 minutes attempting various methods to read the file, including using the pklload function (error on missing config.json file) and pickle.load (errors on installation of the indra module). It would be much more useful for the authors to distribute the Benchmark Corpus in some plain text format and through some recognized data repository (e.g., zenodo, figshare). The ability for readers to easily inspect the Benchmark Corpus is particularly important given the extremely high AUPRC values in Table 6.

We thank the reviewer for pointing this out. INDRA needs to be installed (pip install indra) to be able to load the Benchmark Corpus pickle file. As suggested by the reviewer, we made the Benchmark Corpus as well as the aggregated curations used for belief estimation available on Zenodo (10.5281/zenodo.7559353). In addition to Python pickle files, we made both the corpus and the curations available as JSON which are human-readable and can be loaded in non-Python environments. Finally, we improved the documentation on the repository accompanying the manuscript at https://github.com/sorgerlab/indra_assembly_paper.

Minor comments

* The manuscript is quite long, to the point that the key messages may get lost to less focused readers. The authors are in the best position to decide what, if anything, could be removed or moved to supplemental materials while still preserving the overall intent. (This reviewer suggests Figure 1 could be a candidate.)

In the revision we have worked to improve flow and minimize reader exhaustion. However, given all of the questions about Figure 1 raised by Reviewer 1 we believe that (as revised) it plays an important role in grounding the approach conceptually and making the case for the general concept of the INDRA assembly pipeline. We have done our best to integrate Figure 1 better with the corresponding text.

* A clearer description of the data model behind a Statement would be useful. Both Box 1 and Figure 3A seem to have this intent, but they differ in enough ways that make it unclear to the reader (e.g., "Entities" vs "Agents").

We added a new “INDRA Statement representation” section in Methods to summarize the INDRA Statement and Agent representation. We also added a new Table EV1 to summarize what types of Statements are obtained from each reading system in the Benchmark Corpus.

* (Pages 8-10) Each normalization and filtering step is important, and they could be described in much more detail. If those methods are described in prior work, they could be cited. If not, more details should be added to the Methods or Supplementary Methods.

To address this, we added a new Methods section called "Normalization and filtering of INDRA Statements" where each normalization and filtering step is described in more detail, citing prior work where appropriate, and also referring to INDRA's software module documentation.

* Figure 4D: If space permits, changing the ambiguous "Belief" label in the legend to "INDRA Belief" or "INDRA Belief Model" would be clearer.

Thank you, this is a good idea – we have therefore changed the label to read "INDRA Belief" in Figure 4D, as suggested by the reviewer.

* The Discussion could benefit from a bit more contextualization of the results in the broader space of knowledge graph construction. With AUPRCs of > 0.9, has the problem of automatically building these KGs at massive scale a solved issue then? Are there caveats in the results that may temper enthusiasm? (For example, could the selection of the negative examples in the Curate Corpus based on incorrect statements from the readers be somehow leading to an overestimate of the performance? Or the fact that the positive examples were based on results from individual readers?)

Regarding the AUPRCs, we added two additional sections to the Results clarifying that the values obtained are expected to be high due to the stratified sampling used to generate the statement curation dataset. When introducing the curated dataset, we now note: "*Statements were sampled in a stratified manner by mention count in order to establish the relationship between mention count and reliability; high mention-count Statements are therefore overrepresented relative to their baseline frequency in the Benchmark Corpus (see "Statement Curation" section of Methods).*"

Then when introducing the AUPRC values: "*Model comparisons were based on the area under the precision-recall curve (AUPRC), which is a more robust metric than the area under the receiver-operator curve (AUROC) for class-imbalanced data (~73% of Statements in our curated corpus were correct). In interpreting the AUPRC values, it is important to recall that the curated corpus is, by construction, biased towards Statements with higher mention counts and therefore greater reader overlap: for example, Statements supported by only a single reader constitute 81% of the Benchmark corpus (Table 4) but only 35% of the curated corpus (see "Statement Curation" section of Methods). Reported AUPRCs should therefore be interpreted primarily as a measure of the relative performance of different models across Statements supported by different combinations of readers*."

In the discussion we also note several additional limitations, including representation ("[Our method] does not currently represent genetic interactions, gene-disease relationships, biomarkers, or other types of statistical associations.") and the significant additional work to account for additional types of unreliability involving individual interactions and assembled knowledge graphs, including polarity conflicts, contradictions, and the strengths and weaknesses of the underlying scientific studies.

Overall, we believe that additional work will be required to overcome persistent problems with reader accuracy, even in a multi-reader approach, address remaining issues with grounding, extending INDRA to additional classes of mechanisms, capturing biological context in a principled manner, and building comprehensive KGs that can be used for causal inference, not just statistical analysis.

References

Bachman, J. A., Gyori, B. M., & Sorger, P. K. (2018). FamPlex: A resource for entity recognition and relationship resolution of human protein families and complexes in biomedical text mining. *BMC Bioinformatics*, *19*(1), 248. https://doi.org/10.1186/s12859-018-2211-5

Bachman, J. A., Sorger, P. K., & Gyori, B. M. (2022). *Assembling a corpus of phosphoproteomic annotations using ProtMapper to normalize site information from databases and text mining* [Preprint]. bioRxiv. https://doi.org/10.1101/822668

Cohen, P. R. (2015). DARPA's Big Mechanism program. *Physical Biology*, *12*(4), 045008. https://doi.org/10.1088/1478-3975/12/4/045008

Gyori, B. M., Bachman, J. A., Subramanian, K., Muhlich, J. L., Galescu, L., & Sorger, P. K. (2017). From word models to executable models of signaling networks using automated assembly. *Molecular Systems Biology*, *13*(11), 954. https://doi.org/10.15252/msb.20177651

Gyori, B. M., Hoyt, C. T., & Steppi, A. (2022). Gilda: Biomedical entity text normalization with machine-learned disambiguation as a service. *Bioinformatics Advances*, *2*(1), vbac034. https://doi.org/10.1093/bioadv/vbac034

Peterson, M., Korves, T., Garay, C., Kozierok, R., & Hirschman, L. (2022). *Final Report on MITRE Evaluations for the DARPA Big Mechanism Program*. https://doi.org/10.48550/ARXIV.2211.03943

Valenzuela-Escárcega, M. A., Babur, Ö., Hahn-Powell, G., Bell, D., Hicks, T., Noriega-Atala, E., Wang, X., Surdeanu, M., Demir, E., & Morrison, C. T. (2018). Large-scale automated machine reading discovers new cancer-driving mechanisms. *Database: The Journal of Biological Databases and Curation*, *2018*. https://doi.org/10.1093/database/bay098

Thank you for sending us your revised manuscript. We have now heard back from the three reviewers who were asked to evaluate your revised study. As you will see below, the reviewers are satisfied with the performed revisions and support publication.

Before we formally accept the study for publication, we would ask you to address some remaining editorial issues listed below.

Reviewer #1:

I think that the authors have satisfactorily addressed my comments and the comments of the other reviewers. The manuscript is ready for publication.

Reviewer #2:

The authors addressed all my previous comments and I congratulate them to a very nice article.

Reviewer #3:

The revised manuscript has addressed all of my previous concerns. I believe this work represents a valuable scientific contribution and is suitable for publication.

All editorial and formatting issues were resolved by the authors.

2nd Revision - Editorial Decision 27th Feb 2023

Thank you again for sending us your revised manuscript. We are now satisfied with the modifications made and I am pleased to inform you that your paper has been accepted for publication.

EMBO Press Author Checklist

Corresponding Author Name: Peter K Sorger; Benjamin M. Gyori (co-corresponding)
Journal Submitted to: Molecular Systems Biology
Manuscript Number: MSB-2022-11325

USEFUL LINKS FOR COMPLETING THIS FORM
The EMBO Journal - Author Guidelines
EMBO Reports - Author Guidelines
Molecular Systems Biology - Author Guidelines
EMBO Molecular Medicine - Author Guidelines

Reporting Checklist for Life Science Articles (updated January
This checklist is adapted from Materials Design Analysis Reporting (MDAR) Checklist for Authors. MDAR establishes a minimum set of requirements in transparent reporting in the life sciences (see Statement of Task: 10.31222/osf.io/9sm4x). Please follow the journal's guidelines in preparing your
Please note that a copy of this checklist will be published alongside your article.

Abridged guidelines for figures
1. Data
The data shown in figures should satisfy the following conditions:
- the data were obtained and processed according to the field's best practice and are presented to reflect the results of the experiments in an accurate and unbiased manner.
- ideally, figure panels should include only measurements that are directly comparable to each other and obtained with the same assay.
- plots include clearly labeled error bars for independent experiments and sample sizes. Unless justified, error bars should not be shown for technical
- if n<5, the individual data points from each experiment should be plotted. Any statistical test employed should be justified.
- Source Data should be included to report the data underlying figures according to the guidelines set out in the authorship guidelines on Data

2. Captions
Each figure caption should contain the following information, for each panel where they are relevant:
- a specification of the experimental system investigated (eg cell line, species name).
- the assay(s) and method(s) used to carry out the reported observations and measurements.
- an explicit mention of the biological and chemical entity(ies) that are being measured.
- an explicit mention of the biological and chemical entity(ies) that are altered/varied/perturbed in a controlled manner.
- the exact sample size (n) for each experimental group/condition, given as a number, not a range;
- a description of the sample collection allowing the reader to understand whether the samples represent technical or biological replicates (including how many animals, litters, cultures, etc.).
- a statement of how many times the experiment shown was independently replicated in the laboratory.
- definitions of statistical methods and measures:
  - common tests, such as t-test (please specify whether paired vs. unpaired), simple $\chi 2$ tests, Wilcoxon and Mann-Whitney tests, can be unambiguously identified by name only, but more complex techniques should be described in the methods section;
  - are tests one-sided or two-sided?
  - are there adjustments for multiple comparisons?
  - exact statistical test results, e.g., P values = x but not P values < x;
  - definition of 'center values' as median or average;
  - definition of error bars as s.d. or s.e.m.

Please complete ALL of the questions below.
Select "Not Applicable" only when the requested information is not relevant for your study.

Materials

Newly Created Materials	Information included in the manuscript?	In which section is the information available? (Reagents and Tools Table, Materials and Methods, Figures, Data Availability Section)
New materials and reagents need to be available; do any restrictions apply?	Not Applicable	

Antibodies	Information included in the manuscript?	In which section is the information available? (Reagents and Tools Table, Materials and Methods, Figures, Data Availability Section)
For **antibodies** provide the following information:<br>- Commercial antibodies: RRID (if possible) or supplier name, catalogue number and or/clone number<br>- Non-commercial: RRID or citation	Not Applicable	

DNA and RNA sequences	Information included in the manuscript?	In which section is the information available? (Reagents and Tools Table, Materials and Methods, Figures, Data Availability Section)
**Short novel DNA or RNA including primers, probes**: provide the sequences.	Not Applicable	

Cell materials	Information included in the manuscript?	In which section is the information available? (Reagents and Tools Table, Materials and Methods, Figures, Data Availability Section)
**Cell lines:** Provide species information, strain. Provide accession number in repository **OR** supplier name, catalog number, clone number, and/**OR** RRID.	Not Applicable	
**Primary cultures:** Provide species, strain, sex of origin, genetic modification status.	Not Applicable	
Report if the cell lines were recently **authenticated** (e.g., by STR profiling) and tested for mycoplasma contamination.	Not Applicable	

Experimental animals	Information included in the manuscript?	In which section is the information available? (Reagents and Tools Table, Materials and Methods, Figures, Data Availability Section)
**Laboratory animals or Model organisms:** Provide species, strain, sex, age, genetic modification status. Provide accession number in repository **OR** supplier name, catalog number, clone number, **OR** RRID.	Not Applicable	
**Animal observed in or captured from the field:** Provide species, sex, and age where possible.	Not Applicable	
Please detail **housing and husbandry conditions**.	Not Applicable	

Plants and microbes	Information included in the manuscript?	In which section is the information available? (Reagents and Tools Table, Materials and Methods, Figures, Data Availability Section)
**Plants:** provide species and strain, ecotype and cultivar where relevant, unique accession number if available, and source (including location for collected wild specimens).	Not Applicable	
**Microbes:** provide species and strain, unique accession number if available, and source.	Not Applicable	

Human research participants	Information included in the manuscript?	In which section is the information available? (Reagents and Tools Table, Materials and Methods, Figures, Data Availability Section)
If collected and within the bounds of privacy constraints report on age, sex and gender or ethnicity for all study participants.	Not Applicable	

Core facilities	Information included in the manuscript?	In which section is the information available? (Reagents and Tools Table, Materials and Methods, Figures, Data Availability Section)
If your work benefited from core facilities, was their service mentioned in the acknowledgments section?	Not Applicable	

Design

Study protocol	Information included in the manuscript?	In which section is the information available? (Reagents and Tools Table, Materials and Methods, Figures, Data Availability Section)
If study protocol has been **pre-registered, provide DOI in the manuscript**. For clinical trials, provide the trial registration number **OR** cite DOI.	Not Applicable	
Report the **clinical trial registration number** (at ClinicalTrials.gov or equivalent), where applicable.	Not Applicable	

Laboratory protocol	Information included in the manuscript?	In which section is the information available? (Reagents and Tools Table, Materials and Methods, Figures, Data Availability Section)
Provide DOI OR other citation details if **external detailed step-by-step protocols** are available.	Not Applicable	

Experimental study design and statistics	Information included in the manuscript?	In which section is the information available? (Reagents and Tools Table, Materials and Methods, Figures, Data Availability Section)
Include a statement about **sample size** estimate even if no statistical methods were used.	Not Applicable	
Were any steps taken to minimize the effects of subjective bias when allocating animals/samples to treatment (e.g. **randomization procedure**)? If yes, have they been described?	Not Applicable	
Include a statement about **blinding** even if no blinding was done.	Not Applicable	
Describe **inclusion/exclusion criteria** if samples or animals were excluded from the analysis. Were the criteria pre-established?<br><br>If sample or data points were omitted from analysis, report if this was due to attrition or intentional exclusion and provide justification.	Not Applicable	
For every figure, are **statistical tests** justified as appropriate? Do the data meet the assumptions of the tests (e.g., normal distribution)? Describe any methods used to assess it. Is there an estimate of variation within each group of data? Is the variance similar between the groups that are being statistically compared?	Yes	Statistical tests were used in Figure 3D and E to study the distribution of mentions per Statement, with details provided in the Results section accompanying the figure. Statistical tests were also used to combine multiple DepMap experimental conditions and to perform multiple hypothesis testing. Details are provided in the Results and Materials and Methods sections.

Sample definition and in-laboratory replication	Information included in the manuscript?	In which section is the information available? (Reagents and Tools Table, Materials and Methods, Figures, Data Availability Section)
In the figure legends: state number of times the experiment was **replicated** in laboratory.	Not Applicable	
In the figure legends: define whether data describe **technical or biological replicates**.	Not Applicable	

Ethics

Ethics	Information included in the manuscript?	In which section is the information available? (Reagents and Tools Table, Materials and Methods, Figures, Data Availability Section)
Studies involving **human participants**: State details of **authority granting ethics approval** (IRB or equivalent committee(s), provide reference number for approval.	Not Applicable	
Studies involving **human participants**: Include a statement confirming that **informed consent** was obtained from all subjects and that the experiments conformed to the principles set out in the WMA Declaration of Helsinki and the Department of Health and Human Services Belmont Report.	Not Applicable	
Studies involving **human participants:** For publication of **patient photos**, include a statement confirming that consent to publish was obtained.	Not Applicable	
Studies involving experimental **animals**: State details of **authority granting ethics approval** (IRB or equivalent committee(s), provide reference number for approval. Include a statement of compliance with ethical regulations.	Not Applicable	
Studies involving **specimen and field samples:** State if relevant **permits** obtained, provide details of authority approving study; if none were required, explain why.	Not Applicable	

Dual Use Research of Concern (DURC)	Information included in the manuscript?	In which section is the information available? (Reagents and Tools Table, Materials and Methods, Figures, Data Availability Section)
Could your study fall under dual use research restrictions? Please check biosecurity documents and list of **select agents and toxins** (CDC): https://www.selectagents.gov/sat/list.htm	Not Applicable	
If you used a select agent, is the security level of the lab appropriate and reported in the manuscript?	Not Applicable	
If a study is subject to dual use research of concern regulations, is the name of the **authority granting approval and reference number** for the regulatory approval provided in the manuscript?	Not Applicable	

Reporting

The MDAR framework recommends adoption of discipline-specific guidelines, established and endorsed through community initiatives. Journals have their own policy about requiring specific guidelines and recommendations to complement MDAR.

Adherence to community standards	Information included in the manuscript?	In which section is the information available? (Reagents and Tools Table, Materials and Methods, Figures, Data Availability Section)
State if relevant guidelines or checklists (e.g., **ICMJE, MIBBI, ARRIVE, PRISMA**) have been followed or provided.	Not Applicable	
For **tumor marker prognostic studies,** we recommend that you follow the **REMARK** reporting guidelines (see link list at top right). See author guidelines, under 'Reporting Guidelines'. Please confirm you have followed these guidelines.	Not Applicable	
For **phase II and III randomized controlled trials**, please refer to the **CONSORT** flow diagram (see link list at top right) and submit the CONSORT checklist (see link list at top right) with your submission. See author guidelines, under 'Reporting Guidelines'. Please confirm you have submitted this list.	Not Applicable	

Data Availability

Data availability	Information included in the manuscript?	In which section is the information available? (Reagents and Tools Table, Materials and Methods, Figures, Data Availability Section)
Have **primary datasets** been deposited according to the journal's guidelines (see 'Data Deposition' section) and the respective accession numbers provided in the Data Availability Section?	Yes	In section "Availability of data and material"
Were **human clinical and genomic datasets** deposited in a public access-controlled repository in accordance to ethical obligations to the patients and to the applicable consent agreement?	Not Applicable	
Are **computational models** that are central and integral to a study available without restrictions in a machine-readable form? Were the relevant accession numbers or links provided?	Not Applicable	
If publicly available data were reused, provide the respective **data citations in the reference list**.	Yes	References provided in the manuscript
